# Beyond one-to-one mappings: Modelling distributed lesion-symptom relationships with multilayer networks

**DOI:** 10.1016/j.nicl.2026.104052

**Published:** 2026-08-26

**Authors:** Roxane Nave, Sam Ng, Sylvie Moritz-Gasser, Lorelei Berger, Emmanuel Mandonnet, Hugues Duffau, Guillaume Herbet

**Affiliations:** a Institute of Functional Genomics, University of Montpellier, INSERM, CNRS, 141 rue de la Cardonille, 34091 Montpellier, France; b Department of Neurosurgery, Gui de Chauliac Hospital, Montpellier University Medical Center, 80 Av Augustin Fliche, 34295 Montpellier, France; c Praxiling laboratory, UMR 5267, CNRS, Paul Valéry – Montpellier 3 University, rue de Mende, 34090 Montpellier, France; d Department of neurosurgery, Lariboisière Hospital, Paris, France; e Frontlab, Paris Brain Institute, CNRS UMR 7225, INSERM U1127, Paris, France; f Université de Paris Cité, Paris, France; g University of Montpellier, Department of Medicine, Campus ADV, 641, avenue du Doyen Gaston Guiraud, 34090 Montpellier, France; h Institut Universitaire de France, Paris, France

**Keywords:** Lesion–symptom mapping, Multilayer network analysis, Structural disconnection, Cognitive deficits, Low-grade glioma

## Abstract

Lesion–symptom mapping is widely used to identify causal relationships between brain structures and behaviour, and has played a central role in neuropsychologically informed network models of cognition. However, even recent approaches remain constrained by a one-to-one mapping framework, which oversimplifies the complex relationships between network-level damage and cognitive deficits. In addition, the non-orthogonality of cortical and white matter damage makes it difficult to disentangle their distinct contributions. Here, we used graph-based multilayer network analysis to address these limitations and evaluate clinical relevance. Using neuroanatomical and longitudinal neuropsychological data from 252 patients who underwent awake neurosurgery for low-grade glioma, we constructed interactive, three-layer networks for each hemisphere. Layer 1 comprised neuropsychological tasks (NT), layer 2 structural disconnections (SD), and layer 3 cortical damage (CD). Nodes represented tasks, white matter tracts, and cortical parcels, respectively, whereas within-layer edges captured correlations in performance or co-occurring damage patterns. Multilayer community detection identified domain- and hemisphere-specific brain–behaviour motifs linking executive, language, and spatial functions to distinct combinations of cortical and white matter disruption, a pattern confirmed by two spatial embedding approaches. Centrality analyses revealed a continuum of mapping relationships, ranging from one-to-one to one-to-many associations, indicating that tasks such as verbal fluency are better explained by multiple disconnection mechanisms. Additional analyses uncovered many-to-one and many-to-many relationships and highlighted tracts and cortical regions with domain-general relevance. Together, these findings support a neurobiologically grounded, network-oriented account of how structural brain damage gives rise to cognitive deficits, with implications for clinical care.

## Highlights

• Multilayer networks model cortical damage, disconnection, and cognition jointly.
• Disconnections predicted deficits more strongly than cortical damage.
• Brain–behaviour relationships extended beyond simple one-to-one mappings.
• Left and right hemispheres showed distinct lesion–behaviour motifs.
• Many-to-many mappings predominated across tasks and damaged structures.
• Specific tracts emerged as domain-general hubs across deficits.

## 1 Introduction

The remarkable diversity and flexibility of human cognition are rooted in the brain's intricate anatomo-functional architecture. Rather than operating through isolated modules, the brain works as a multiscale, adaptive network, comprising distributed yet functionally specialized systems, whose dynamic integration supports learning, resilience, and behavioural adaptation (Bassett and Gazzaniga, 2011; Bullmore and Sporns, 2009; Herbet and Duffau, 2020; Park and Friston, 2013; Shine and Poldrack, 2018). Advances in systems neuroscience and connectomics have further reinforced this view, depicting the brain as a complex, hierarchically organized system—broadly analogous in its organizing principles to other biological and engineered systems (Bullmore and Sporns, 2009; Telesford et al., 2011).

Within this framework, a central challenge in cognitive and computational neuroscience is to understand how interactions among brain structures, network topology, and neural computations give rise to cognitive functions. Equally important is the development of methods capable of capturing these complex relationships (Genon et al., 2022). This challenge is particularly critical in clinical populations, where focal or progressive lesions disrupt the brain's architecture in patterned yet heterogeneous ways. Lesion–symptom mapping remains a foundational approach for inferring structure–function relationships in patients with brain damage, and recent multivariate or machine learning-based techniques have enabled more refined, data-driven analyses (Bates et al., 2003; Mirman et al., 2015; Pustina et al., 2018; Zhang et al., 2014). Yet many of these approaches still rest—implicitly or explicitly—on simplifying assumptions, most notably that cognitive functions map discretely onto individual brain structures. Mounting evidence instead indicates that cognition emerges from dynamic interactions among distributed, multifunctional systems (Park and Friston, 2013; Pessoa, 2014). Traditional one-to-one mapping frameworks thus capture only a narrow aspect of brain–function relationships, overlooking core organizational principles of complex biological systems such as pluripotentiality, degeneracy, and multi-relationality.

A second key limitation of current lesion-symptom mapping approaches concerns the unresolved issue of non-orthogonality between cortical and white matter damage. Disentangling their distinct contributions to neuropsychological deficits remains challenging, given the intrinsic interdependence between cortical regions and the fiber pathways connecting them. While tract–focused lesion–symptom mapping approaches offer valuable insights into disconnection-related effects, most studies continue to assess lesion location and structural disconnection separately rather than integrating both dimensions within a *unified* framework (Herbet et al., 2015; Moore et al., 2024). In addition, there is evidence suggesting that these two forms of structural damage do not contribute equally to behavioural impairment. In large prospective stroke cohorts, Corbetta et al. demonstrated that behavioural deficits cluster into a small number of broad dimensions driven primarily by subcortical anatomy and white matter disconnection rather than by cortical lesion topography, directly challenging syndrome-based accounts grounded in focal cortical damage (Corbetta et al., 2015). Building on this, Salvalaggio et al. showed that structural disconnection estimated from normative tractography adds predictive value over focal lesion location across multiple behavioural domains, in ways that cannot be captured by lesion maps alone (Salvalaggio et al., 2020). Crucially, evidence from the glioma surgery literature converges on the same conclusion through a different and particularly compelling lens: cortical damage in low-grade glioma patients can remain behaviorally silent, whereas white matter disconnection is a robust and lasting predictor of cognitive deficits (Cochereau et al., 2020).

A third challenge arises from the anatomically and pathophysiologically constrained distribution of brain lesions. Ischemic strokes commonly affect regions supplied by the middle cerebral artery (Corbetta et al., 2015), while tumours such as low-grade gliomas preferentially involve the anterior temporal lobe, insula, and prefrontal cortex (Herbet et al., 2016a). As a result, brain damage is often clustered within specific vascular or anatomical territories and rarely affects functional networks in isolation. They frequently span multiple, spatially contiguous cortical and white matter regions, complicating efforts to isolate the contribution of any single structure. This collinearity poses a fundamental challenge for causal inference and can obscure underlying structure–function relationships. Moreover, cognitive impairments may not arise from damage to a single region, but from disruptions across multiple interacting systems (Warren et al., 2014).

A fourth, and often overlooked limitation concerns the neuropsychological measures used in lesion–symptom analyses (Ferguson, 2022; Herbet et al., 2024). Many studies rely on performance from single behavioural tasks that are often outdated and poorly aligned with contemporary cognitive theory. These tasks are typically multi-determined, while being interpreted as measuring a single function. To address this, dimensionality reduction techniques such as Principal Component Analysis (PCA) have been applied to extract latent cognitive dimensions (Halai et al., 2017; Zhao et al., 2020), thereby improving the reliability of structure–function associations. However, even these approaches often retain a modular view of cognition, treating cognitive dimensionalities as independent components. This neglects the integrative nature of cognition, wherein interdependencies between domains are central to real-world behaviour. As a result, most lesion studies do not consider how brain damage alters the relationships between tasks or cognitive domains. Collectively, these limitations highlight the need for methods that capture the complex, network-level architecture of brain–behaviour relationships in lesion data.

**Fig. 1:**
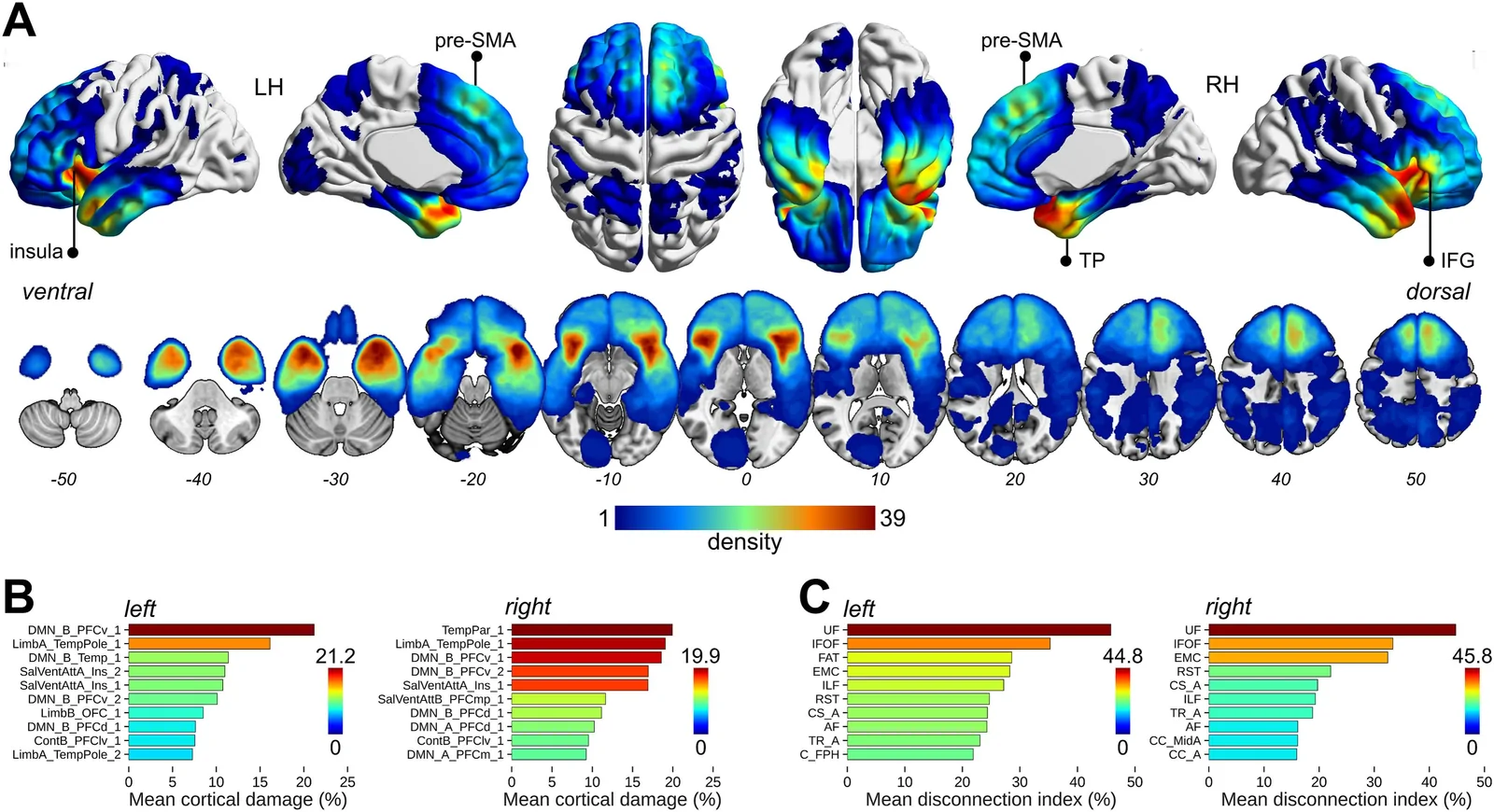
Spatial distribution of surgical resections and detailed lesion data. (A) Topographic distribution of surgical resections across the cohort (*n* = 252), with a color intensity (Jet palette) indicating resection density. Peak density was observed in the insula and the temporal pole, bilaterally. Abbreviations: LH = left hemisphere; RH = right hemisphere; SMA = Supplementary motor area; TP = temporal lobe; IFG = inferior frontal gyrus. (B) Distribution of the top ten most affected cortical regions. (C) Distributions of the top ten most affected white matter tracts. Color bars are thresholded such that the highest intensity corresponds to the greatest degree of damage. See Supplementary Table 1 for a complete glossary of abbreviations. For completeness, the full distributions of cortical and white matter damage, including individual patient data, are provided in Supplementary Fig. 1.

**Fig. 2:**
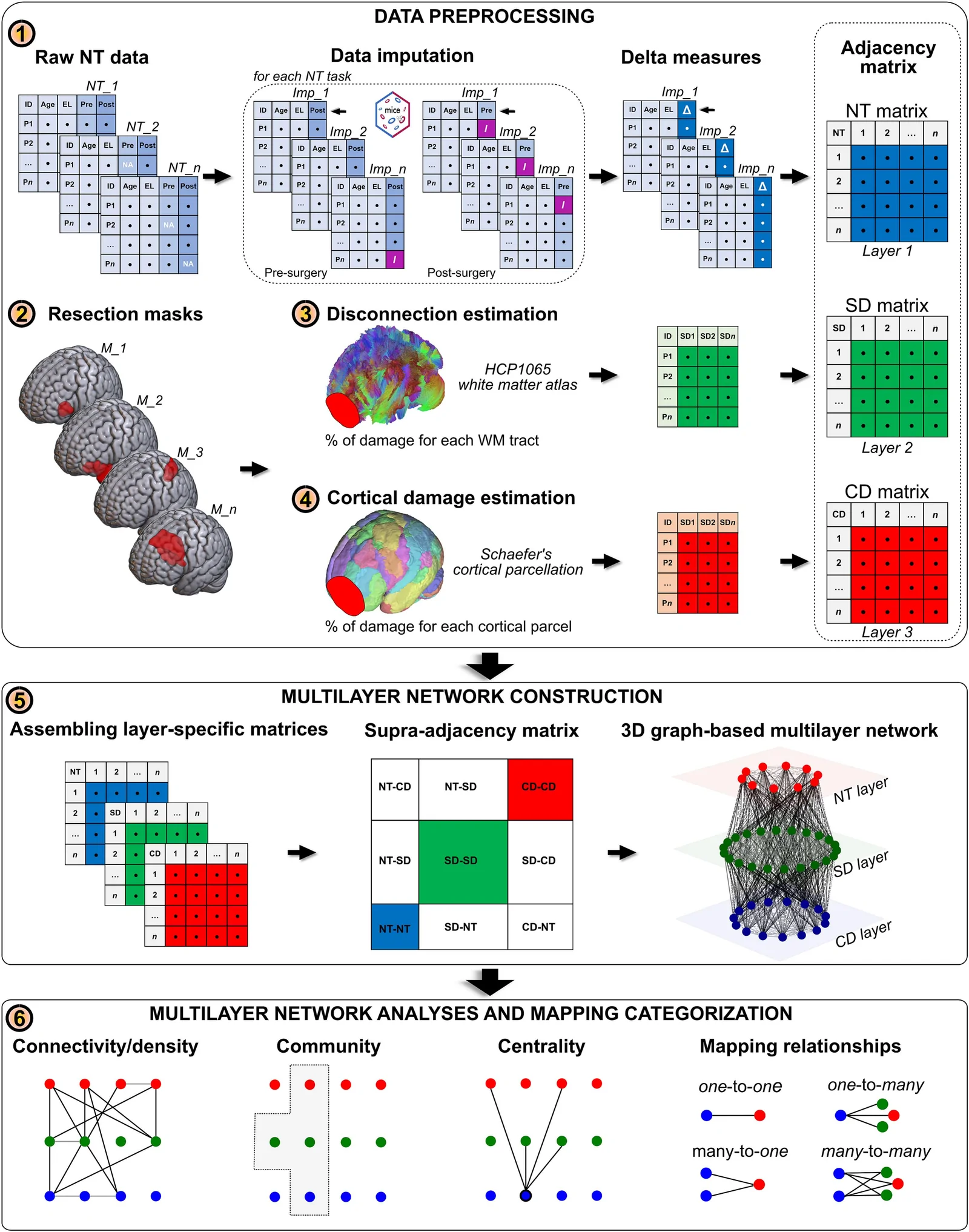
Simplified methodological workflow. (1) Raw neuropsychological data were collected before and three months after surgery. Missing values were addressed through independent imputation of the pre- and post-surgery datasets using the MICE Toolbox. A delta measure (the difference between post- and presurgical assessments) was computed to isolate the effect of neurosurgery on performance. Subsequently, the influence of sociodemographic factors was regressed out, the data were normalized and an adjacency matrix of neuropsychological performance (NT) was generated. (2) Three-month post-surgery 3D T1-weighted MRIs were co-registered to MNI-152 space using enantiomorphic normalization, and resection cavities were manually delineated with MRIcron software. (3) The resulting resection masks were used to quantify the extent of white matter tract damage, using both the HCP1065 white matter atlas and the Lesion Quantification Toolbox. (4) The same toolbox was applied to estimate resection loads based on the Schaefer cortical parcellation atlas (100 parcels; 17 networks). A Structural Disconnection (SD) adjacency matrix and a Cortical Damage (CD) matrix were generated. (5) Based on the layer-specific adjacency matrices (i.e., NT, SD, and CD), a supra-adjacency matrix was constructed and used to generate a multilayer graph using Python NetworkX libraries; (6) three complementary multilayer network analyses were conducted, including connectivity and density analyses, multilayer community detection, and NT-centered centrality analyses. As a final step, lesion-behaviour mapping relationships were categorized to characterize different patterns of anatomo-functional dependence. See *Appendix A* for a detailed description of the graph-based measures used in this study, including their formal definitions and their interpretation in terms of lesion–behaviour inferences.

To tackle these challenges, we introduce a network-based, non-reductionist framework for modelling lesion–behaviour relationships. Specifically, we apply graph-based multilayer network analysis to identify brain–behaviour motifs — distinct, recurring patterns that systematically link cortical lesions, white matter disconnection, and cognitive outcomes. We test this approach in a large and clinically homogeneous cohort of patients who underwent neurosurgery for low-grade glioma, a pathophysiological model that offers variability in lesion location, network disruption, and behavioural outcomes (Fig. 1; Supplementary Fig. 1). While graph theory has been widely applied to model brain connectomes (Bullmore and Bassett, 2011; Seguin et al., 2023) and is increasingly applied to cognitive and psychological data (Borsboom et al., 2021; Ferguson, 2022), its application to jointly model anatomical damage and behavioural profiles within a multilayer framework remains underexplored in clinical populations. By capturing the multidimensional relationships embedded in lesion data (Fig. 2), our approach aims to move beyond simple structure-based attribution to uncover higher-order network motifs that shape cognitive outcomes. More specifically, we asked whether this framework could reveal lesion–behaviour relationships extending beyond simple one-to-one mappings, whether behavioural impairment was more strongly associated with structural disconnection than with cortical damage, and whether distinct domain- and hemisphere-specific brain–behaviour motifs could be identified. We further expected that some neuropsychological measures would show distributed rather than focal anatomical associations, consistent with one-to-many, many-to-one, or many-to-many mapping patterns. Accordingly, rather than testing predefined structure–symptom hypotheses, we adopt a theory-guided, exploratory framework designed to reveal network-level structure–function relationships that cannot be captured using conventional lesion–symptom analyses.

## 2 Material and method

### 2.1 Participants

Some of the cognitive and clinical data included in this study were previously published (Ng et al., 2024); However, the analyses presented here are entirely original, with no overlaps in findings. The retrospective use of clinical data was approved by our Institutional Ethics Committee (NEUROLOGIE DC-2013-2027) and an independent Institutional (Review Board National French College of Neurosurgery, approval no. 00011687–2021/18).

The patient cohort comprised 252 individuals (115 females; age, 39.14 ± 10.83, educational attainment, 14.48 ± 3.00) diagnosed with Diffuse Low-Grade Glioma (DLGG; See Supplementary Table 1 for definition of all used acronyms or abbreviations), who underwent awake neurosurgery with intraoperative electrostimulation mapping for tumor resection at Montpellier University Medical Centre (Montpellier, France), between September 2012 and January 2023 (see Table 1). Inclusion criteria were: (i) confirmed DLGG diagnosis via post-surgical histomolecular analysis; (ii) surgical resection guided by electrostimulation mapping; sxand (iii) completion of both preoperative and 3-month postoperative neuropsychological assessments (patients with fewer than four subtests with longitudinal data were excluded). Exclusion criteria included previous brain surgery or radiotherapy, pre-existing neurological (e.g., stroke) or neurodevelopmental disorders, and non–French-speaking patients.

**Table 1:** Sociodemographic variables.

Hemi	n	Gender		Age		Education	
		Female	Male	mean	SD	mean	SD
Total	252	115	137	39.14	10.83	14.48	3.00
RH	126	59	67	40.00	10.74	15.00	3.01
LH	126	56	70	38.63	10.94	14.11	2.95

Note: LH = left hemisphere; RH = right hemisphere. The LH and the RH groups did not differ statistically in terms of Age (*t*_250_ = 1.003. *p* = 0.37. IC95% [−1.320. 4.060]) but did differ in terms of educational attainment (*t*_250_ = 2.37. *p* = 0.012. IC95% [0.151. 1.629]). The proportion of females and males did not differ between the groups (*χ*^2^ = 0.064. *p* = 0.16). Education attainment was computed as years of schooling. Note that the relatively high educational level of the sample may be partly related to the fact that some patients sought care at highly specialized and well-established clinical centers, a self-initiated process that may be associated with higher levels of education.

### 2.2 Surgical procedure

For completeness, we briefly outline the surgical procedure, although intraoperative data are not analyzed in the current study. All patients underwent tumor resection under an asleep–awake–asleep protocol, including intraoperative Direct Electrical Stimulation (DES) mapping. Following craniotomy and dura opening, DES was applied using a bipolar probe (NIMBUS stimulator, Newmedic; 5 mm inter-tip spacing) delivering biphasic currents (60 Hz, 1 ms pulse width, 1.50–3.50 mA amplitude). The surgical procedure was guided by real-time functional responses obtained through DES at both cortical and subcortical levels, adhering to principles of functionally guided tumor resection. Tumor removal ceased when stimulation elicited transient disruptions of motor, language, or cognitive functions at the margins of the resection cavity. Detailed descriptions of intraoperative tasks used for functional mapping and monitoring have been published elsewhere (Sarubbo et al., 2020; Tate et al., 2014).

### 2.3 Neuropsychological assessment and missing data handling

All patients completed a neuropsychological evaluation the day before surgery and again three months postoperatively. The assessment comprised a battery of 19 cognitive tests and subtests routinely used in clinical practice to measure episodic memory, executive functions, attention, language, and visuospatial skills (see Supplementary Table 2 for a detailed description of each task and the corresponding explanation of the cognitive and behavioural skills they assess). Due to factors such as patient fatigue and organizational constraints, longitudinal data were partially incomplete (in average, 14.38%, range 1.19–32.54 across measures), which is common in clinical datasets from neurological populations (Austin et al., 2021) (see Supplementary Table 3).

To address missing cognitive data, we applied Multiple Imputation by Chained Equations (MICE) using the *mice* package in R (version 4.1.3). Importantly, imputation was not intended to “recover” ground-truth individual scores, but rather to preserve the multivariate structure of the neuropsychological battery and to enable downstream analyses that require complete cognitive matrices while maintaining statistical power. MICE generates plausible estimates for missing entries through iterative conditional models, preserving variability and reducing potential bias compared to simpler imputation methods (Van Buuren, 2011). Following recommendations by van Buuren et al. (2011), we generated five imputed datasets (10 iterations) and used Predictive Mean Matching (PMM), which is suitable for cognitive outcomes with mixed discrete/continuous properties. Age and educational attainment were included as predictors in the imputation models, given their known influence on cognitive performance (see Supplementary Tables 4–5 and Supplementary Fig. 2 for diagnostics). No anatomical variables used in the subsequent structure-function analyses were included as predictors in the imputation model to avoid circularity.

Imputation was performed separately for preoperative and postoperative datasets, because missingness differed across timepoints (some patients missing only preoperative scores, others only postoperative scores, and a smaller subset missing both). Joint cross-wave imputation would borrow information between timepoints and therefore relies more strongly on a stable cross-time predictive structure. In our cohort, postoperative cognitive trajectories were highly heterogeneous, with improvement, decline, or stability varying across patients and tasks, plausibly influenced by surgical factors (e.g., location and extent of resection). We therefore imputed pre- and postoperative data independently so that each timepoint could be modeled under its own missingness structure without imposing strong cross-time assumptions.

For each imputed dataset and neuropsychological subtest, differences between preoperative and postoperative scores (i.e. delta scores) were computed to quantify surgery-related cognitive changes (see Supplementary Table 6 for details). Delta scores were then adjusted for age and education using linear regression and normalized. For visualization and downstream analyses, we used the mean across the five imputations to obtain a single cognitive matrix per hemisphere.

To make the influence of imputation transparent, the distributions of scores across tasks, hemispheres and imputations are reported in Supplementary Table 7 and 8, including complete-case data. In these tables, we additionally report pre-post pairwise comparisons to identify measures showing group-level postoperative changes. The distributions of delta scores across imputations, stratified by task and hemisphere, are shown in Supplementary Fig. 3.

### 2.4 Neuroimaging data acquisition and preprocessing

Three-dimensional T1-weighted MRI scans were systematically acquired at the three-month postoperative follow-up, coinciding with neuropsychological assessments. All MRI images were spatially normalized to the Montreal Neurological Institute (MNI152) standard template using enantiomorphic normalization from the Clinical Toolbox for SPM12 (https://www.nitrc.org/projects/clinicaltbx), implemented in MATLAB (Release 2022a; The MathWorks Inc., Natick, MA, USA). Prior to normalization, resection cavity maps were manually delineated on postoperative 3D T1-weighted images using MRIcron software (http://www.mccauslandcenter.sc.edu/mricro/mricron). These lesion masks were normalized by applying the transformation matrix obtained during image normalization, resulting in three-dimensional volumes of interest with a 1-mm isotropic resolution. The normalized volumes were subsequently used in all analyses and visualizations.

### 2.5 Estimation of grey matter parcel resection loads

Grey matter resection loads were estimated using the Lesion Quantification Toolkit (LQT) (Griffis et al., 2021), based on Schaefer et al.'s resting-state fMRI-derived cortical atlas, which parcellates the cortex into 135 regions across 17 functional networks (Schaefer et al., 2018). For each patient, the percentage of resected tissue was computed for each cortical parcel. To ensure reliability and interpretability in subsequent modelling, only parcels meeting the following criteria were retained: at least 20% of patients had non-zero damage in the parcel with a minimum of 5% of the parcel damaged (a complete list of cortical areas included is provided in Supplementary Table 6).

### 2.6 Estimation of structural disconnections

White matter tract damage was estimated using the Lesion Quantification Toolkit (LQT)^33^, which applies population-based tractography to quantify disconnection severity at the tract level. Postoperative resection cavity maps were embedded into the HCP-1065 tractography atlas (http://brain.labsolver.org/diffusion-mri-templates/tractography; April 2020 version) and served as regions of interest for estimating structural disconnections. To ensure robustness and avoid arbitrary tract selection, only white matter tracts meeting the same criteria that for parcel selection were retained (Supplementary Table 9).

### 2.7 Data pooling and supra-adjacency matrix construction

Following the collection of neuropsychological scores, cortical damage metrics, and white matter disconnection measures, data pooling was conducted for each hemisphere. This hemisphere-specific approach was motivated by the partial asymmetry of the neuropsychological battery across surgical groups: language and verbal tasks were systematically prioritized for left-hemisphere patients, whereas visuospatial attention and neglect-related measures were more specifically relevant to right-hemisphere patients, alongside a core set of tasks common to both groups. Pooling both hemispheres within a single network would therefore have combined non-equivalent behavioural spaces and lesion–behaviour relationships.

Each modality was first represented as a patient x variable matrix: neuropsychological scores, tract disconnection severity, or cortical parcel lesion load. Pairwise Spearman correlations (*ρ*) were then computed across patients for all pairs of variables yielding weighted adjacency matrices. Within-layer correlations thus represent associations among variables of the same domain, while between-layer correlations reflect associations between variables from different domains. These correlations were combined to form the supra-adjacency matrix for each hemisphere, which served as the basis for subsequent network-based analyses.

### 2.8 Multilayer network visualization

To intuitively represent the complex relationships among cortical damage, structural disconnections, and neuropsychological performance, multilayer network visualizations were generated. Each network layer corresponded to a distinct data domain: *Layer 1* (NT) – nodes represented neuropsychological tasks, and edges encoded performance correlations between tasks; *Layer 2 (SD)* - nodes correspond to white matter tracts, and edges represented correlations in co-occurring disconnection patterns; *Layer 3* (CD) - nodes represented cortical parcels, and edges captured correlations in co-occurring damage between cortical regions. These visualizations enabled the simultaneous exploration of within- and across-layer interactions, offering insight into how structural lesions relate to behavioural impairments.

Network construction and visualization were implemented using the *NetworkX* library (https://networkx.org/) in Python, a flexible library for analyzing and visualizing complex and multilayer network structures. Two multilayer networks, one per hemisphere, were generated from the average supra-adjacency matrices. To focus on meaningful associations and reduce visual noise, only edges with a correlation coefficient | *ρ* | ≥ 0.175 (corresponding to *p* ≤ 0.05; uncorrected; *n* = 126 patients per hemisphere) were included for visualization.

This multilayer framework was designed to integrate complementary anatomical and behavioural domains rather than to construct a multiplex network with one-to-one correspondence across layers, which was not feasible in the present cohort given the absence of patient-specific diffusion tractography and functional connectivity data for all patients.

### 2.9 Network analysis

To quantify the structural properties of the multilayer networks, we conducted a series of graph-theoretical analyses using the *NetworkX* package. Unless otherwise specified, graph analyses were conducted on weighted supra-adjacency matrices derived from Spearman correlation coefficients. Binarized graphs were used only for density estimation and threshold-based classification of brain–behaviour mapping patterns.

#### 2.9.1 Connectivity and density analyses

To investigate connectivity patterns within and across domains, we quantified correlation-based connectivity across the three network layers. Analyses were performed separately for each hemisphere, considering each single layer (NT, SD, CD), each pairwise cross-layer combination (NT-SD, NT-CD, SD-CD), and the full multilayer network (NT-SD-CD). Edge weights corresponded to pairwise Spearman correlation coefficients (*ρ*) computed across patients between node values. For each hemisphere and edge set, mean connectivity was defined as the average edgewise correlation: within-layer means were computed from the upper triangle of the corresponding submatrix (excluding the diagonal), whereas between-layer means were computed from the full rectangular block connecting the two layers. Descriptive summary statistics were reported.

We then tested two predefined contrasts using non-parametric permutation testing (10,000 resamples; two-sided). First, motivated by lesion–symptom work suggesting that cognitive outcomes may be more tightly coupled to white matter disconnection than to cortical damage, we tested within each hemisphere whether mean NT–SD connectivity exceeded mean NT–CD connectivity, using the difference in mean edgewise correlation as the test statistic. Second, to assess hemispheric lateralization of NT-related coupling, we compared left versus right mean connectivity for NT, NT–SD, and NT–CD, again using differences in mean edgewise correlation as test statistics. At each resample, edge weights were randomly reassigned between the two conditions being compared while preserving sample sizes to generate an empirical null distribution, and two-sided *p*-values were computed as the proportion of permuted mean differences with absolute value greater than or equal to the observed value. We report both raw permutation *p*-values and Benjamini–Hochberg FDR–corrected *p*-values, with FDR applied separately to within-hemisphere and between-hemisphere comparisons.

To further characterize the global structural organization of the multilayer network, we computed network density, defined as the proportion of suprathreshold connections relative to the total number of possible connections in a given graph. Density provides an estimate of how tightly coupled a set of nodes is, and allows us to compare the relative interdependence of domains within the multilayer network and to contrast coupling patterns between hemispheres.

Densities were calculated for each single layer (NT, SD, CD), each pairwise combination of layers (NT–SD, NT–CD, SD–CD), and the full multilayer network, separately for the left and the right hemispheres. Starting from the edge-weight supra-adjacency matrix, we binarized connections by applying an absolute threshold such that an edge was considered present when |R| > threshold (diagonal excluded). The primary threshold corresponded to | *ρ* | > 0.175 (*p* = 0.05, uncorrected), and robustness was evaluated across a range of thresholds (|*ρ* | > 0.147, 0.175, 0.229, 0.250, 0.290; nominal *p* = 0.1, 0.05, 0.01, 0.005, 0.001, respectively). Importantly, these thresholds were used to define binary graphs for density estimation and density-based comparisons, not for edge-wise statistical inference.

Because layers differed in node count, densities were estimated using a node-matched subsampling procedure. For each hemisphere and threshold, the analysis was repeated 1000 times using matched node samples. For within-layer and full-network density, the sample size was set to the global minimum across the three layers; for between-layer density, it was set to the pairwise minimum of the two layers being compared. Densities were summarized as mean ± SD across iterations.

Finally, density differences between layers, layer-pairs, and hemispheres were assessed using Monte Carlo permutation tests (10,000 iterations; two-sided). For each comparison, the test statistic was the difference in density (difference in proportions of suprathreshold edges) between the two graph types being contrasted. Null distributions were generated by random reassignment of binary edge indicators between conditions while preserving the number of possible edges per condition, and *p*-values were computed as the proportion of permuted differences whose absolute value was greater than or equal to the observed difference. Because density was used as a global network-level characterization and robustness analysis across thresholds, FDR correction was applied separately to within-hemisphere and between-hemisphere comparisons, across all density comparisons and thresholds considered.

#### 2.9.2 Multilayer community detection

We used a data-driven approach to community detection to uncover meaningful groupings of nodes based on network structure. Unlike clustering algorithms such as *k*-means or hierarchical clustering, which require predefined cluster numbers, community detection allows both the number and composition of communities to emerge from the data. Specifically, we used the Louvain algorithm, a method that partitions the network by maximizing modularity, a measure of the extent to which nodes are more densely connected within communities rather than between communities. The algorithm proceeds iteratively in two phases: first, nodes are reassigned to neighboring communities when such reassignment increases modularity; second, the resulting communities are aggregated into super-nodes, and the procedure is repeated until no further modularity improvement is possible. This approach yields a data-driven partition of the multilayer network into densely interconnected communities.

The main analyses were conducted on weighted networks using the default resolution parameter (γ = 1.0). To evaluate the influence of resolution on community structure, we repeated the Louvain analysis across γ values ranging from 0.5 to 2.0 in steps of 0.25. For each γ value, the algorithm was run 100 times with different random initializations. We quantified the number of detected communities, modularity, and partition stability using mean pairwise normalized mutual information (NMI) across runs.

As an additional robustness analysis, we repeated Louvain community detection after block-wise normalization of retained edge weights within the supra-adjacency matrix, in order to reduce potential effects of differences in average connectivity magnitude across layers and layer-pairs.

#### 2.9.3 Geometric cross-validation of Louvain community structure using UMAP and t-SNE

To cross-validate the results of graph-based community detection analyses, we applied two complementary nonlinear dimensionality reduction techniques: Uniform Manifold Approximation and Projection (UMAP) and t-distributed Stochastic Neighbor Embedding (t-SNE). These methods project high-dimensional multilayer network features into a two-dimensional space, providing an interpretable geometric representation of node similarity and structure-functional organization across layers. UMAP constructs a low-dimensional embedding that preserves both local and global structure, making it suitable for capturing broader network patterns while maintaining fine-grained relationships between nodes. UMAP was implemented with Python's *umap-learn* library with the following parameters: random_state = 80, n_neighbors = 5, min_dist = 0.5. In contrast, t-SNE prioritizes local neighborhood preservation and is effective for representing subtle community boundaries in continuous feature space. t-SNE was implemented with Python's *scikit-learn* library, with the following parameters: n_components = 2, random_state = 80, perplexity = 5, early_exaggeration = 12. Converging results across these methods served as a form of methodological triangulation, reinforcing the robustness and interpretability of the identified network community structure.

To evaluate whether the community structure identified by the Louvain algorithm is reflected in the geometry of the multilayer feature space, we examined the spatial arrangement of Louvain communities in the resulting UMAP and t-SNE embeddings. We further performed extensive parameter sweeps (t-SNE perplexity 5–50 in steps of 5; UMAP n_neighbors 5–30 in steps of 5 × min_dist 0.1/0.5) to assess the robustness of this correspondence. For each embedding configuration, silhouette coefficients were computed using the Louvain community assignments as labels. These coefficients were used not as clustering metrics, but as descriptive indices quantifying whether the spatial organization of Louvain communities was preserved across different UMAP/t-SNE parameter settings. This procedure allowed us to determine both whether the embeddings visually reflected the Louvain community structure and whether this correspondence remained stable across a broad range of hyperparameters.

#### 2.9.4 Multilayer centrality of neuropsychological task nodes

To further characterize node-level importance within the multilayer networks, we computed NT-centered centrality using two complementary weighted network measures: weighed degree centrality (strength) and weighted closeness centrality. Weighted degree centrality captures the overall strength of a node's direct connections by summing the weight of its edges, whereas weighted closeness centrality reflects how efficiently a node can communicate with all others in the network, taking account both the presence and strength of connections along shortest paths. For weighted closeness, edge lengths were defined as d_*ij*_ = 1 – r_*ij*_, such that stronger correlations corresponded to shorter path lengths.

Given our particular interest in the interactions between individual neuropsychological task (NT) nodes and lesion-derived layers, we computed weighted degree and closeness centrality values for each NT node with respect to both the structural disconnection (SD) and cortical damage (CD) layers, as well as their combination. This procedure allowed us to assess whether NT nodes were more strongly coupled with white matter disconnections, cortical damage, or their combined effects.

From a clinical perspective, NT-centered degree centrality quantifies how many damaged brain structures are directly linked to a given neuropsychological deficit, whereas NT-centered closeness centrality indexes sensitivity of that deficit to distributed, cumulative, and indirect lesion effects. Consequently, centrality measures provide a window into the network architecture underlying brain-behaviour relationships. Specifically, they allow us to assess whether a given neuropsychological deficit is associated with a limited number of damaged brain regions or tracts, suggesting a more focal, one-to-one mapping, or whether it is influenced by damage across multiple anatomical structures, reflecting a more distributed, one-to-many pattern of functional dependence.

Centrality values were computed for each NT node, hemisphere, and layer configuration, and summarized across NT nodes using distributional statistics. To test for differences between layer configurations and hemispheres, we used permutation-based inference. Results were additionally explored qualitatively using HTML-based interactive network visualizations, in which NT node size was scaled in proportion to the centrality metric, providing an intuitive means for the qualitative inspection of NT node importance within the multilayer network structure.

#### 2.9.5 Classification of brain–behaviour mapping patterns

To characterize the lesion–deficit relationships linking NT nodes to lesion-based features, we systematically classified the pattern of anatomical connections between NT nodes and nodes in the SD and CD layers. Connections were defined based on pairwise Spearman correlations computed from the supra-adjacency matrices, with a primary threshold set at *R* ≥ 0.175 (*p* ≤ 0.05, uncorrected), and supplementary analyses conducted across a range of thresholds to assess robustness and sensitivity. Each NT node was assigned to one of five mapping categories, separately for its relationships with SD and CD layers: (i) *One-to-one*: The NT node was connected to a single anatomical node (SD or CD), and that node was exclusively connected to that NT node; (ii) *One-to-many*: The NT node was connected to multiple anatomical nodes, each uniquely associated with that NT node; (iii) *Many-to-one*: The NT node was connected to a single anatomical node that was also linked to other NT nodes; (iv) *Many-to-many*: The NT node was connected to multiple anatomical nodes, with at least one node shared with other NT nodes: (iv) *None*: The NT node showed no connections exceeding the defined threshold.

This classification procedure was repeated for multiple correlation thresholds (*R* = 0.147, 0.175, 0.229, 0.250, 0.290, corresponding to uncorrected *p*-values of 0.1, 0.05, 0.01, 0.005, and 0.001, respectively). Thresholding was applied symmetrically for both hemispheres, enabling the examination of lateralization effects and the stability of mapping patterns under varying statistical constraints.

To identify the anatomical structures most frequently associated with behavioural deficits, we also quantified the number of NT nodes connected to each SD and CD node. This allowed for the identification of anatomical “hubs” disproportionately targeted across cognitive tasks, potentially reflecting domain-general functional relevance. All classification outputs were generated separately for the left and right hemispheres.

## 3 Results

### 3.1 Multilayer network construction and visualization

**Fig. 3:**
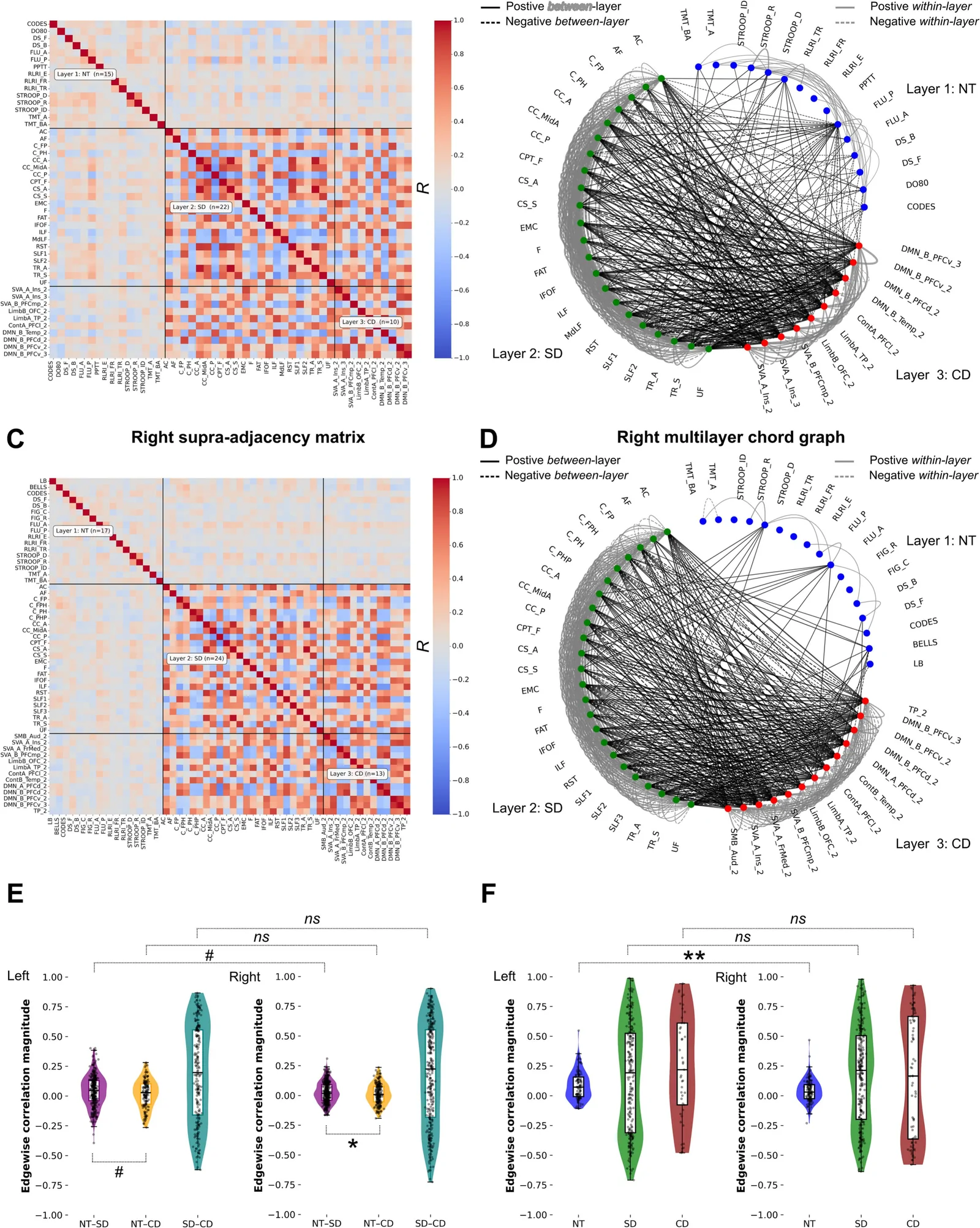
Multilayer graph-based representation of the relationship between cortical damage, structural disconnections and neuropsychological performances in the left and the right hemisphere. (A) Supra-adjacency matrix for the left hemisphere showing the correlation structure across the three layers: neuropsychological task performance (NT), structural disconnections (SD), and cortical damage (CD). The heatmap depicts correlation coefficients (R), with red indicating positive correlations and blue indicating negative correlations. The diagonal blocks represent within-layer correlations, while off-diagonal blocks depict between-layer associations. (B) Multilayer chord diagram for the left hemisphere. Nodes are color-coded by layer (blue: NT, green: SD, red: CD). Edges represent correlations exceeding a threshold (|R| > 0.175), with solid lines indicating positive correlations and dashed lines indicating negative correlations. Black lines represent between-layer associations while grey lines represent within-layer associations. (C) Supra-adjacency matrix for the right hemisphere, structured identically to (A), showing within-layer and between-layer correlations. (D) Multilayer chord diagram for the right hemisphere. (E) Raincloud plots showing the distributions of between-layer edgewise correlation magnitudes for the NT-SD, NT-CD and SD-CD layer pairs in the left and right hemisphere. # means marginal effect; * indicates statistically significant effect at *p* < 0.05 (10,000 permutation test). (F) Raincloud plots (Violin, boxplot and individual datapoints) showing the distributions of within-layer edgewise correlation magnitudes for NT, SD and CD in the left and right hemispheres.

To explore the relationships between neuropsychological task performance (NT), structural disconnections (SD), and cortical damage (CD), we constructed multilayer network models separately for each hemisphere (left hemisphere: Fig. 3A–B and Supplementary Fig. 4 A-B; right hemisphere: Fig. 3C–3D and Supplementary Fig. 4C—D). These multilayer models capture within- and between-layer interactions, providing a comprehensive representation of lesion–behaviour relationships. Networks were visualized using supra-adjacency matrices, multilayer graphs, multilayer chord diagrams, and two-dimensional between-layer projections to clarify the structural relationships among the three layers (NT, SD, CD). Interactive versions of these networks are available online to dynamically explore the network architecture (https://github.com/jdax34/scripts_article/tree/main/Article), and hierarchical clustering of individual layers is presented in Supplementary Fig. 5.

### 3.2 Single-layer and cross-layer connectivity patterns

We computed connectivity measures separately for each network layer (NT, SD, CD), for each layer pair (NT–SD, NT–CD, SD–CD), and for the combined multilayer networks, in both hemispheres (detailed statistics are provided in Supplementary Tables 10 and 11). Overall, the NT layer consistently showed lower connectivity than SD and CD layers within each hemisphere (Fig. 3B, left hemisphere; Fig. 3D, right hemisphere). The SD–CD pair exhibited higher connectivity values compared to NT–SD and NT–CD pairs in both hemispheres (Supplementary Fig. 4 A-B, left hemisphere; Supplementary Fig. 2C—D, right hemisphere).

To determine whether cognitive performance was more strongly related to structural disconnections than to cortical damage, we compared NT–SD and NT–CD connectivity. To determine whether cognitive performance was more strongly related to structural disconnections than to cortical damage, we compared NT–SD and NT–CD connectivity. Permutation test indicated a trend toward stronger NT–SD than NT–CD connectivity in both hemispheres (left: mean difference = 0.023, *p* = 0.065, pFDR = 0.066; right: mean difference = 0.0146, *p* = 0.048, pFDR = 0.066; Fig. 3E), although these effects did not survive FDR correction.

Between hemispheres, NT connectivity was higher in the left than in the right hemisphere (mean difference = 0.0417, *p* = 0.003, *p*_FDR_ = 0.009), indicating stronger covariance among NT measures on the left (Fig. 3F). NT–SD connectivity also tended to be higher on the left (mean difference = 0.0142, *p* = 0.08; *p*_FDR_ = 0.13, Fig. 3E), whereas NT-CD connectivity did not differ between hemispheres (mean difference = 0.0056, *p* = 0.56, *p*_FDR_ = 0.56).

### 3.3 Network density and cross-layer interdependence

We calculated network density, defined as the proportion of actual connections relative to all possible connections in a network, separately for each layer (NT, SD, CD), pairwise layer combinations, and the full multilayer network (Supplementary Tables 12 and 13). Nodes were considered connected if their correlation exceeded a threshold of |R| ≥ 0.175 (*p* ≤ 0.05, uncorrected). The SD and CD layers exhibited high densities across hemispheres (SD: left = 84.6%, right = 81.9%; CD: left = 84.4%, right = 96.8%), reflecting stereotyped patterns of structural damage across patients. By contrast, the NT layer displayed markedly lower densities (left = 27.6%, right = 11.4%), indicating greater independence among neuropsychological tasks, particularly in the right hemisphere.

Interlayer density analysis, measuring the interconnectedness between layers, revealed strong associations between SD and CD layers in both hemispheres (left = 76.8%, right = 77.2%). However, the relationships between structural damage and neuropsychological performance were substantially weaker: NT–SD and NT–CD connections were relatively sparse in the left hemisphere (17.3% and 14.7%) and even lower in the right hemisphere (4.9% and 4.5%). This asymmetry highlights notably weaker structure–performance relationships in the right hemisphere.

To ensure these findings were not driven by unequal node counts, we performed node-controlled subsampling and repeated the analysis across multiple ∣R∣ thresholds to mitigate any bias from threshold choice. Permutation tests indicated a robust, threshold-independent hierarchy (SD ≈ CD ≫ NT), with the SD-SCD connections consistently denser than NT-involving connections, and hemispheric differences most observed for NT-SD and NT-CD, while SD-CD showed non hemispheric asymmetry (Supplementary Fig. 6 and Supplementary Table 14 for full descriptive statistics and *p*-values).

### 3.4 Multilayer community detection of lesion-behaviour relationships

**Fig. 4:**
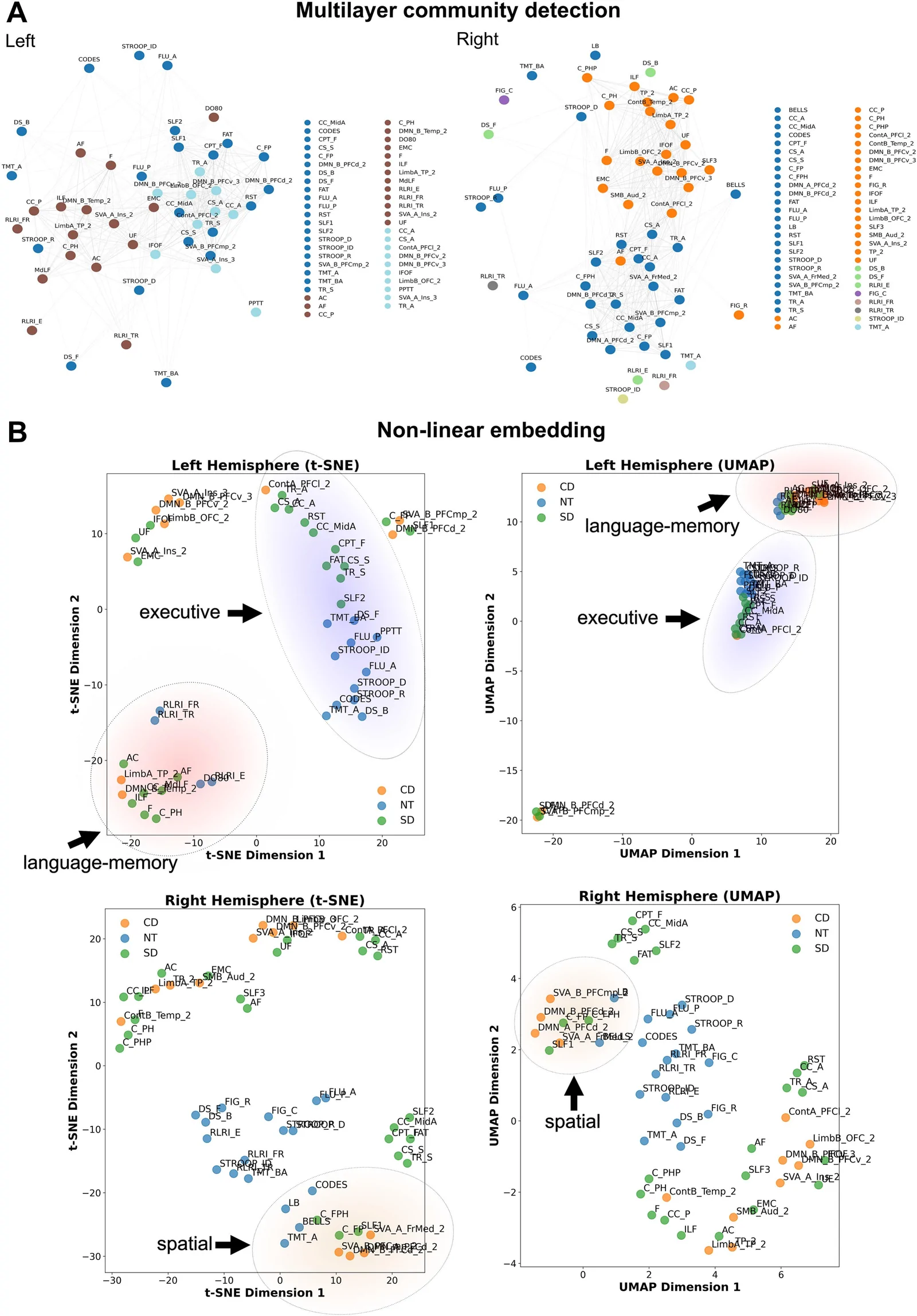
Multilayer community detection and 2D nonlinear-embedding. (A) Multilayer Community Detection: The left and the right column display a network graph in which nodes are color-coded by their community membership (three distinct communities), for the left and the right hemisphere respectively. (B) The left and right columns present t-SNE and UMAP projections, respectively. In these representations, nodes from the three layers cluster together, reflecting structured lesion–symptom relationships. In the left hemisphere, two principal clusters are observed, mainly associated with language–memory and executive domains. In the right hemisphere, a single meaningful cluster integrating nodes from all three layers is identified, mainly associated with the spatial domain.

To explore whether NT, SD and CD cluster into meaningful structure-function relationships, we next applied multilayer community detection—a graph-based clustering method that identifies densely interconnected nodes across multiple network layers. In the left hemisphere, three distinct communities emerged, each including nodes from all three layers (Fig. 4A, left panel; Supplementary Table 15). Community 1 integrated executive and working memory tasks (e.g., digit span, TMT, Stroop, fluency) with frontal, fronto-parietal, and superior fronto-striatal/thalamic tracts (e.g., FAT, SLF2, CS_S, TR_S) and dorsal frontal cortical regions (e.g., DMN_B_PFCd_2, SVA_B_PFCmp_2). Community 2 combined language and memory tasks (e.g., DO80, RLRI), ventral white matter tracts (e.g., ILF, MdLF, UF), and temporal-insular cortical nodes (e.g., DMN_B_Temp_2, LimbA_TP_2). Community 3 grouped semantic tasks (e.g., PPTT), ventral or anterior fronto-striatal/thalamic connections (e.g., IFOF, CS_A, TR_A), and ventral prefrontal cortical regions (e.g., DMN_B_PFCv_2, LimbB_OFC_2).

To verify that these community assignments were also reflected in the intrinsic geometry of the multilayer feature space, we generated low-dimensional embeddings using t-SNE and UMAP (Fig. 4B). In the left hemisphere, both techniques recovered two broad geometric clusters corresponding closely to Communities 1 and 2, with Community 3 occupying an intermediate position. Parameter-sweep analyses confirmed that these geometric relationships were robust: silhouette coefficients computed using Louvain community labels were consistently positive and relatively high across settings (t-SNE: 0.43–0.47; UMAP: 0.21–0.54; Supplementary Table 16).

In RH, multilayer community detection yielded eight communities, with one clearly integrated community spanning all three layers (Fig. 4A, right panel, Supplementary Table 12). This community included tasks associated with spatial processing and ideomotor speed (e.g., Bell test, line bisection, TMT_A, Stroop_R), mesial and dorsal fronto-thalamic/striatal white matter connections (e.g., SLF1, SLF2, C_FP, CS_S), and mesial/dorsal cortical nodes (e.g., SVA_A_FrMed_2, SVA_B_PFCmp_2, DMN_A_PFCd_2). T-SNE and UMAP analyses confirmed this integrated community, highlighting a cohesive cluster combining spatial and ideomotor tasks, medial white matter pathways, and mesial prefrontal cortical regions (Fig. 4B panel). Although the right-hemisphere partition contained more communities, including several very small ones, parameter sweeps nonetheless showed a broadly consistent geometric correspondence: silhouette values remained low but generally positive across settings (t-SNE: −0.01 to 0.19; UMAP: −0.11 to 0.14; Supplementary Table 16), indicating a weaker but stable separation of communities in low-dimensional space.

Finally, a robustness analysis using MNI confirmed the stability of the Louvain partitions, particularly in the right hemisphere (mean NMI = 0.91 ± 0.11) relative to the left hemisphere (mean NMI = 0.80 ± 0.17; Supplementary Table 17). Our sensitivity analysis further showed that, as expected, increasing γ resulted in a greater number of detected communities, reflecting a finer partitioning of the network. However, the overall community structure remained highly stable across resolution values, with partition stability remaining consistently high and increasing further for γ values above 1.0. Importantly, modularity was maximum at γ = 1.0 in both hemispheres (left hemisphere: Q = 0.362; right hemisphere: Q = 0.374), indicating that this parameter provided the best balance between community segregation and global network organization. These findings suggest that the reported community structure is robust across the tested range of γ values.

To assess whether the detected community structure was influenced by differences in connectivity magnitude across layers, we repeated the Louvain analysis after block-wise normalization of retained edge weights within the supra-adjacency matrix. The resulting partition was unchanged in the left hemisphere (NMI = 1.00 relative to the original solution) and remained highly similar in the right hemisphere (NMI = 0.898). Partition stability also remained high after normalization (left mean NMI = 0.815; right mean NMI = 0.937). Together, these findings indicate that the main community structure was not driven by raw differences in block-wise connectivity magnitude across layers.

### 3.5 NT-centered multilayer centrality analyses

**Fig. 5:**
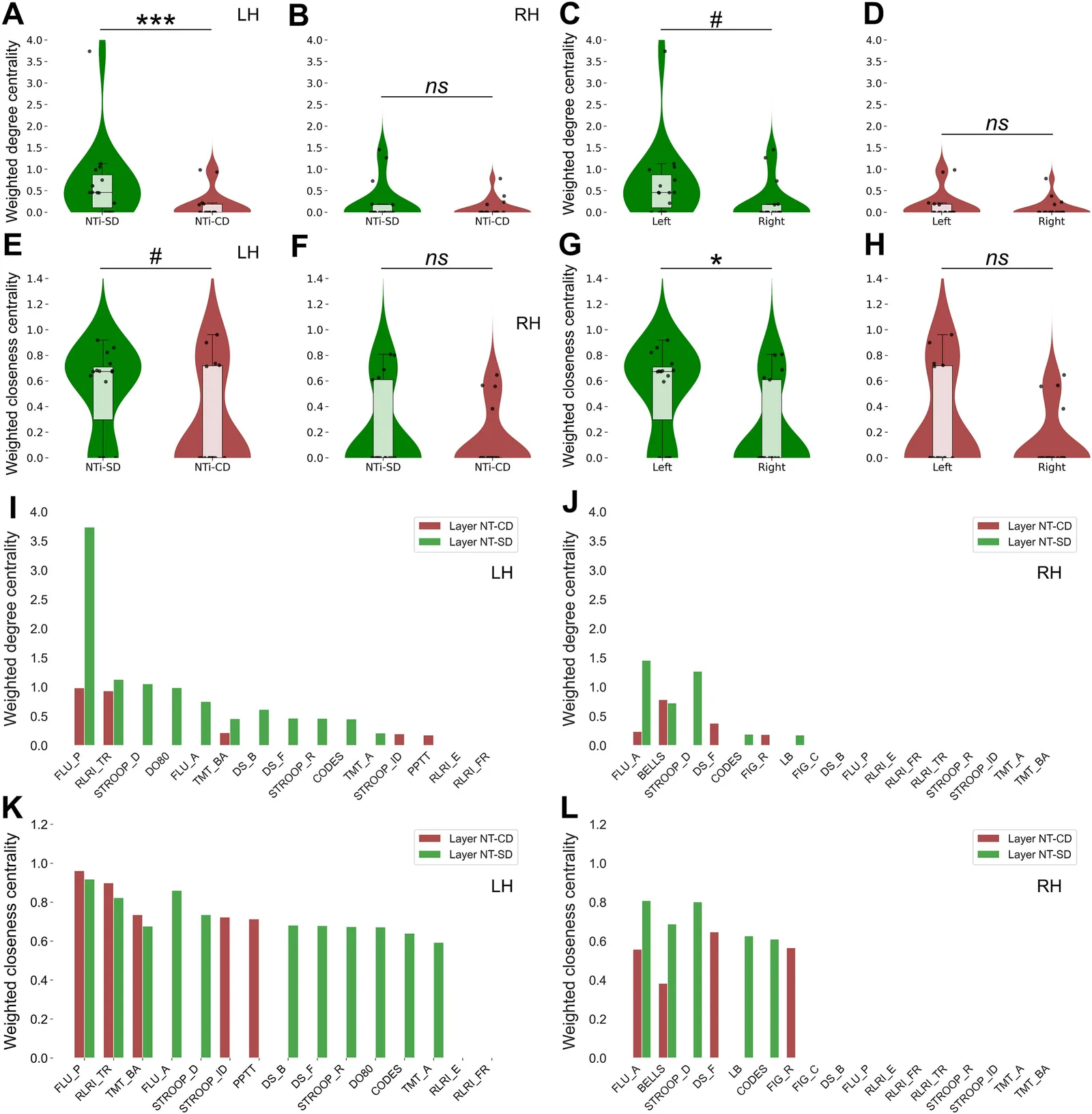
NT-centered multilayer centrality analyses reveal hemispheric and layer-dependent effects. (A-D) Weighted degree centrality of neuropsychological tasks (NT) defined relative to structural disconnection (NTi-SD) and cortical damage (NTi-CD), shown separately for the left (A) and the right (B) hemispheres, and for cross-hemispheric comparisons within each layer (C—D). (*E*-H) Weighted closeness centrality for NTi-SD and NTi-SD, shown by hemisphere (E-F) and in cross-hemispheric comparisons (G-H). (I-L) Task-wise distributions of weighted degree (I-J) and weighted closeness (K-L) centrality for NT nodes in the left (K-L) and right (J-L) hemispheres. Across panels, violin plots show the distribution of NT centrality values, with boxplots indicating median and interquartile range. Statistical comparisons were performed using permutation-based tests; ns, not significant; # *p* < 0.10; * < 0.05; ** *p* < 0.01; *** *p* < 0.001. All results displayed in the figure are fully described in the Results section.

Analyses of NT-centered network centrality revealed layer- and hemisphere-dependent effects that differed across metrics. For weighted degree centrality, NT nodes showed significantly higher centrality when defined relative to structural disconnection (NTi-SD) than to cortical damage (NTi-CD) in the left hemisphere (mean difference = 0.52, *p* = 0.002, p_FDR_ = 0.005; Fig. 5A), indicating that a stronger embedding of NT performance within white matter disconnection networks than cortical damage networks. This effect was not observed in the right hemisphere (mean difference = 0.13, *p* = 0.37, p_FDR_ = 0.37; Fig. 5B). Importantly, NTi-SD centrality showed a near-significant asymmetry, with relatively higher values in the left compared with the right hemisphere (mean difference = 0.46, *p* = 0.06; p_FDR_ = 0.13; Fig. 5C), whereas no hemispheric difference was observed for NTi-CD (mean difference = 0.074, *p* = 0.45; p_FDR_ = 0.45; Fig. 5D). Together, these findings suggest a preferential reliance of NT performance on structural disconnection networks in the left hemisphere, relative to the right.

For weighted closeness centrality, a broadly similar pattern was observed, albeit with weaker statistical support. In the left hemisphere, NT nodes showed a marginal increase in closeness when defined relative to CD compared with SD (left: mean difference = 0.26, *p* = 0.096, p_FDR_ = 0.15; right: mean difference = 0.08, *p* = 0.40, p_FDR_ = 0.40; Fig. 5E and F). The cross hemispheric comparison was significant for NTi-SD, with higher closeness values in the left than in the right hemisphere (mean difference = 0.32, *p* = 0.014, p_FDR_ = 0.02; Fig. 5G), whereas no hemispheric difference was identified for NTi-CD (mean difference = 0.14, *p* = 0.22, p_FDR_ = 0.22; Fig. 5H).

Qualitatively, centrality values (both degree and closeness) spanned a continuous range across NT nodes (Fig. 5I-L). Some tasks showed consistently high centrality - reflecting greater susceptibility to disruption from damage across multiple cortical and white structures -, including letter fluency and RLRI in the left hemisphere, and the Bell test and semantic fluency in the right hemisphere, whereas other tasks showed low or near-zero centrality values. A notably large number of right-hemisphere NT nodes displayed zero centrality, highlighting weaker or absent associations with structural damage and suggesting more localized or less structured lesion–behaviour relationships compared with the left hemisphere. Overall, this graded pattern indicates that some neuropsychological performances are embedded within distributed damage networks involving multiple neural systems, whereas others rely on more specific or circumscribed neural systems or show minimal systematic association with neural systems damaged by resection.

Finally, we examined whether integrating structural disconnection with cortical damage information increases the centrality of NT centrality, relative to considering cortical damage alone, by directly comparing NTi-CD with NTi-CD-SD. For degree centrality, NTi-CD-SD showed higher centrality than NTi-CD in the left (mean difference = −0.69, *p* = 0.0007, p_FDR_ = 0.004) and a trend in the same direction in the right hemisphere (mean difference = −0.22, *p* = 0.06, p_FDR_ = 0.10). The same pattern was observed for closeness centrality, with NTi-CD-SD exceeding NTi-CD in both hemispheres (left: mean difference = −0.36, *p* = 0.008; right: mean difference = −0.16, *p* = 0.014, p_FDR_ = 0.02). By contrast, the inverse contrasts (i.e., NTi–SD vs NTi–SD–CD) were marginal or non-significant, indicating that adding cortical damage information to structural disconnection networks provides limited additional benefit. Together, these results suggest that structural disconnections contribute substantially to NT-centered centrality, and that their integration with cortical damage enhances NT network embedding when starting from cortical damage alone. Detailed data and statistics are provided in Supplementary Table 18–21.

### 3.6 Classification of brain–behaviour mapping patterns

To better characterize the complexity of relationships linking neuropsychological tasks (NT nodes) to SD and cortical damage CD nodes, we classified each NT node into the five distinct mapping categories described previously. We conducted these analyses across multiple correlation thresholds to assess the stability and sensitivity of mapping classifications. Additionally, we determined how frequently individual SD and CD nodes were targeted by NT nodes, identifying anatomical nodes most consistently associated with declined performance.

**Fig. 6:**
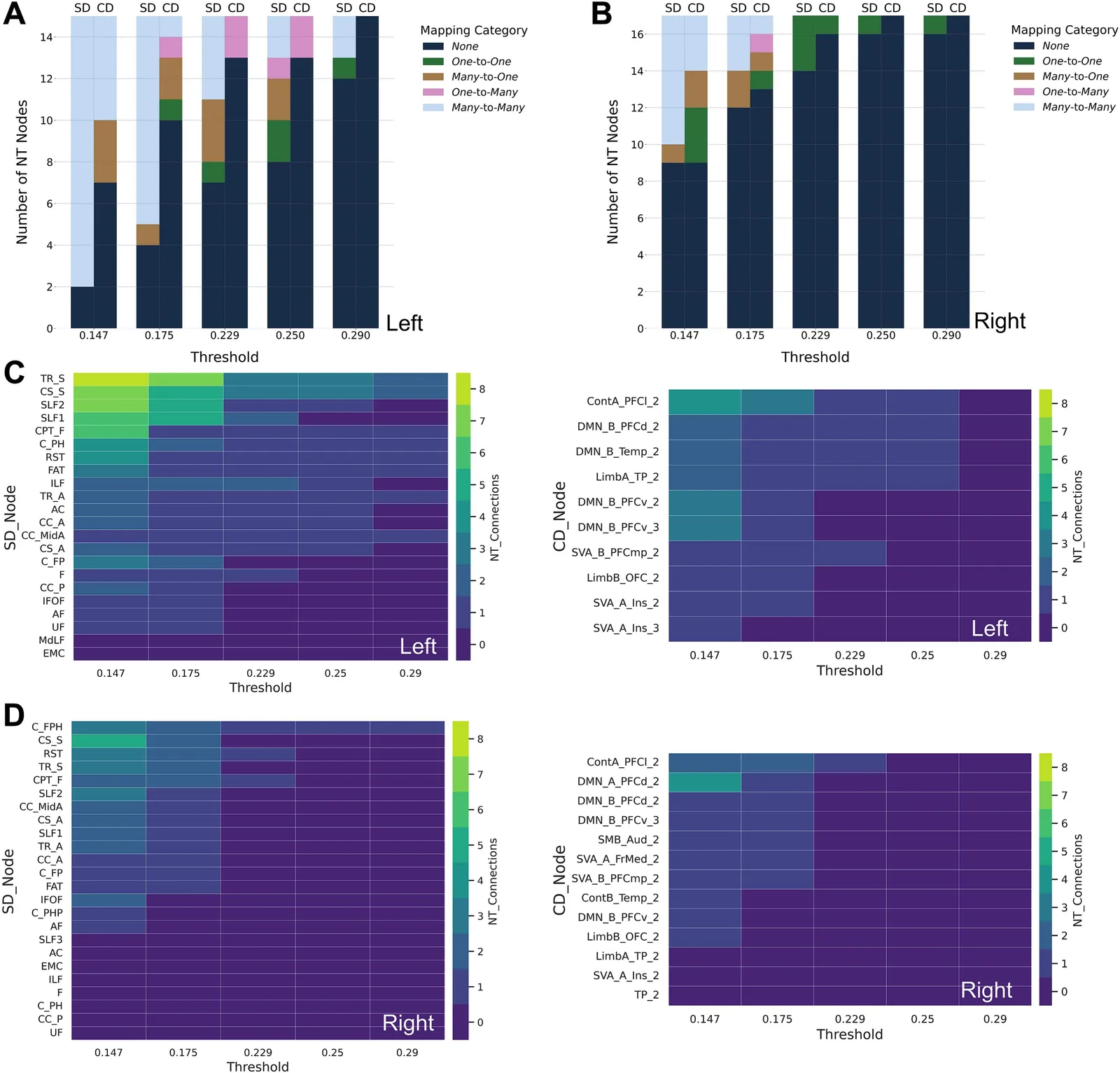
Mapping relationships across layers and thresholds. (A, B) Stacked bar plots illustrating the distribution of mapping categories between neuropsychological test (NT) nodes and structural disconnection (SD) and cortical damage (CD) layers at various correlation thresholds (R-values: 0.147, 0.175, 0.229, 0.250, 0.290; corresponding to *p*-values of 0.1, 0.05, 0.01, 0.005, and 0.001, respectively) for the left (A) and right (B) hemispheres. Mapping categories are defined as follows: (i) one-to-one, a single NT node connects exclusively to one target node, and this target connects only to that NT node; (ii) one-to-many, one NT node connects to multiple targets, all of which are exclusive to that NT node; (iii) many-to-one, multiple NT nodes connect to a shared target node; (iv) many-to-many, one NT node connects to multiple targets, with at least one target shared with other NT nodes; and (v) none, indicating no connections. Using Heatmaps, Panels (C, D) display the total number of NT connections for individual SD (left subpanels) and CD nodes (right subpanels), stratified by hemisphere (left hemisphere in C; right hemisphere in D) and correlation threshold. Cell color encodes the number of connections. Nodes are sorted in descending order by their total number of connections summed across all thresholds to facilitate comparison of the most connected nodes. Note that acronyms for cortical regions and white matter tracts are fully defined in Table and in the glossary.

At the standard threshold of *R* ≥ 0.175 (*p* ≤ 0.05), all mapping categories were present (Table 2), but many-to-many mappings predominated in both the left (Fig. 6A) and right hemispheres (Fig. 6B). Reducing this threshold increased the frequency of many-to-many relationships, reflecting broader, less-specific connections. Conversely, increasing the threshold preserved a more diverse distribution of mapping patterns, particularly within the left hemisphere and notably in the SD layer. Higher thresholds also led to increased instances of “none” mappings, reflecting fewer detectable associations overall.

**Table 2:** Mapping relationships between individual NT nodes and CD and NT layers (threshold ≥0.175, *p* ≤ 0.05).

Hemi	NT tasks	Count		Mapping type		Connections	
		SD	CD	SD	CD	SD	CD
Left	CODES	2	0	many-to-many	none	SLF1, SLF2	
Left	DO80	4	0	many-to-many	none	AF, C_PH, F, ILF	
Left	DS_F	2	0	many-to-many	none	CS_S, TR_S	
Left	DS_B	3	0	many-to-many	none	CS_S, SLF2, TR_S	
Left	FLU_A	4	0	many-to-many	none	C_FP, C_PH, SLF1, TR_S	
Left	FLU_P	12	4	many-to-many	many-to-many	C_FP, CC_A, CC_MidA, CPT_F, CS_A, CS_S, FAT, RST, SLF1, SLF2, TR_A, TR_S	SVA_B_PFCmp_2, ContA_PFCl_2, DMN_B_PFCd_2, DMN_B_PFCv_3
Left	PPTT	0	1	none	many-to-one		ContA_PFCl_2
Left	RLRI_E	0	0	none	none		
Left	RLRI_FR	0	0	none	none		
Left	RLRI_TR	5	4	many-to-many	one-to-many	AC, CC_P, IFOF, ILF, UF	SVA_A_Ins_2, LimbA_TP_2, DMN_B_Temp_2, DMN_B_PFCv_2
Left	STROOP_D	4	0	many-to-many	none	CS_S, SLF1, SLF2, TR_S	
Left	STROOP_R	2	0	many-to-many	none	CS_S, TR_S	
Left	STROOP_ID	0	1	none	one-to-one		LimbB_OFC_2
Left	TMT_A	1	0	many-to-one	none	SLF1	
Left	TMT_BA	2	1	many-to-many	many-to-one	SLF2, TR_S	ContA_PFCl_2
Right	LB	1	0	many-to-one	none	TR_S	
Right	BELLS	3	4	many-to-many	one-to-many	C_FP, C_FPH, SLF1	SVA_A_FrMed_2, SVA_B_PFCmp_2, DMN_A_PFCd_2, DMN_B_PFCd_2
Right	CODES	1	0	many-to-one	none	C_FPH	
Right	DS_F	0	2	none	many-to-many		ContA_PFCl_2, DMN_B_PFCv_3
Right	DS_B	0	0	none	none		
Right	FIG_C	0	0	none	none		
Right	FIG_R	0	1	none	one-to-one		SMB_Aud_2
Right	FLU_A	7	1	many-to-many	many-to-one	CC_A, CC_MidA, CPT_F, CS_A, CS_S, RST, TR_A	ContA_PFCl_2
Right	FLU_P	0	0	none	none		
Right	RLRI_E	0	0	none	none		
Right	RLRI_FR	0	0	none	none		
Right	RLRI_TR	0	0	none	none		
Right	STROOP_D	6	0	many-to-many	none	CPT_F, CS_S, FAT, RST, SLF2, TR_S	
Right	STROOP_R	0	0	none	none		
Right	STROOP_ID	0	0	none	none		
Right	TMT_A	0	0	none	none		
Right	TMT_BA	0	0	none	none		

Abbreviations: *Neuropsychological task acronyms*. BELLS, Bells cancellation test; CODES, Coding subtest (processing speed; e.g. WAIS Coding); DO80, Dénomination Orale – 80, test; DS_B, Digit Span Backward; DS_F, Digit Span Forward; FIG_C, Rey or Taylor Figure Copy; FIG_R, Rey-Osterrieth or Taylor Complex Figure Recall; FLU_P, Phonemic (Letter) verbal fluency; FLU_A, Semantic (Category) verbal fluency; LB, Line Bisection; PPTT, Pyramids and Palm Trees Test; RLRI_E, Rappel libre-rappel indicé task, Encoding; RLRI_FR, Rappel libre-rappel indicé task recall index, Free Recall; RLRI_TR, Rappel libre-rappel indicé task, Total Recall; STROOP_D, Stroop Test, Denomination; STROOP_ID, Interference (difference score); STROOP_R, Stroop Test, Reading; TMT_A, Trail Making Test, Part A; TMT_BA, Trail Making Test, Part B–A (set-shifting cost). *White matter tract acronyms*. AC, Anterior Commissure; AF, Arcuate Fasciculus; CC_A, Corpus Callosum, Anterior; CC_MidA, Corpus Callosum, Mid-Anterior; CC_P, Corpus Callosum, Posterior; C_FP, Fronto–Parietal Cingulum; C_FPH, Fronto–Parietal-Hippocampal Cingulum; C_PH, Parahippocampal Cingulum; C_PHP, Cingulum_Parahippocampal_Parietal; CPT_F, Frontal component of the Corticopontine Tract; CS_A, Corticostriatal Tract, Anterior; CS_S, Corticostriatal tract, Superior; EMC, Extreme Capsule; F, Fornix; FAT, Frontal Aslant Tract; IFOF, Inferior Fronto-Occipital Fasciculus; ILF, Inferior Longitudinal Fasciculus; MdLF, Middle Longitudinal Fasciculus; RST, Reticulo-Spinal Tract; SLF1, Superior Longitudinal Fasciculus I; SLF2, Superior Longitudinal Fasciculus II; SLF3, Superior Longitudinal Fasciculus III; TR_A, Thalamic Radiations, Anterior; TR_S, Thalamic Radiations, Superior; UF, Uncinate Fasciculus. *Functional network/node acronyms*: ContA_PFCl_2, Control Network, lateral Prefrontal Cortex node; ContB_Temp_2, Control Network, temporal node; DMN_A_PFCd_2, Default Mode Network (Anterior), dorsal Prefrontal Cortex node; DMN_B_PFCd_2, Default Mode Network (Posterior), dorsal Prefrontal Cortex node; DMN_B_PFCv_2, Default Mode Network (Posterior), ventral Prefrontal Cortex node; DMN_B_PFCv_3, Default Mode Network (Posterior), ventral Prefrontal Cortex node; DMN_B_Temp_2, Default Mode Network (Posterior), Temporal node; LimbA_TP_2, Limbic Network (Anterior), Temporal Pole node; LimbB_OFC_2, Limbic Network (Posterior), Orbitofrontal Cortex node; SMB_Aud_2, Somatomotor Network, Auditory-related node; SVA_A_FrMed_2, Salience/Ventral Attention Network (Anterior), Medial Frontal node; SVA_A_Ins_2, Salience/Ventral Attention Network (Anterior) II; SVA_A_Ins_3, Salience/Ventral Attention Network (Anterior) III; Insula node; SVA_B_PFCmp_2, Salience/Ventral Attention Network (Posterior), medial Prefrontal Cortex node; TP_2, Tempo-Parietal Network, node 2.

To illustrate these relationships, at the standard threshold, the forward digit span task (assessing verbal short-term memory) was associated with two white matter tracts — the superior branches of the cortico-striatal fasciculus and thalamic radiations. However, both tracts were also linked to several other tasks, such as reading and naming components of the Stroop task and verbal fluency. No significant associations with cortical damage nodes were observed for the digit span task, classifying it as many-to-many with the SD layer but “none” with the CD layer. By contrast, the PPTT, a measure of non-verbal semantic association, was exclusively linked to a single cortical node (the lateral prefrontal cortex within the Control A network), exemplifying a clear one-to-one mapping relationship specifically within the CD layer. Comprehensive results for these analyses are provided in Table 2 (*R* ≥ 0.175, *p* ≤ 0.05) and supplementary Table 22 (*R* ≥ 0.175, *p* ≤ 0.01).

Finally, certain anatomical nodes, particularly white matter tracts and, to a lesser extent, cortical regions, were disproportionately targeted by multiple NT nodes, suggesting a role in domain-general processing. In the left hemisphere, prominent examples included the superior thalamic radiations, superior cortico-striatal connections, superior longitudinal fasciculus branch II, and lateral prefrontal cortex (ContA_PFCl_2) (Fig. 6C). This pattern was considerably less marked in the right hemisphere (Fig. 6D). In brief, these results suggest that multidimensional mapping relationships are common (see Supplementary Tables 23–26).

## 4 Discussion

Understanding how structural brain damage gives rise to cognitive deficits remains a central challenge in cognitive neuroscience and neuropsychology. This issue is especially pressing given that lesion-based methods continue to play a critical role in validating findings from correlational functional neuroimaging studies in healthy individuals (Rorden and Karnath, 2004; Szczepanski and Knight, 2014). Here, we introduce a multilayer network approach for lesion-symptom mapping that jointly models cortical lesions, structural disconnections, and cognitive outcomes in a large cohort of patients with diffuse low-grade glioma. Multilayer network approaches are increasingly recognized in neuroscience as a means of transcending the limitations of single-modality analyses by capturing the interdependencies among diverse biological and behavioural systems (Bassett and Sporns, 2017; De Domenico, 2017; Muldoon and Bassett, 2016). In the context of lesion mapping, they offer a principled framework for modelling how different forms of structural brain damage, such as cortical lesions and white matter damages, interact to shape cognitive impairments, while addressing known limitations of conventional analytical methods. By applying this approach, we demonstrate that brain–behaviour relationships are fundamentally distributed, heterogeneous, and shaped by interactions across lesion modalities. These findings challenge classical one-to-one mapping assumptions and underscore the need for new integrative frameworks that more accurately reflect the complexity of lesion-induced cognitive dysfunction.

Across multiple layers of analysis, we found that behavioural impairments were more strongly associated with structural disconnections than with cortical damage, reinforcing the central role of white matter pathways in supporting cognitive function. Multilayer community detection revealed that neuropsychological tasks clustered with distinct combinations of white matter tracts and cortical regions, giving rise to hemisphere-specific structure–function communities. Centrality analyses further demonstrated that cognitive tasks varied in their degree of anatomical dependence, with some exhibiting high centrality across diverse tracts and regions—indicating broad, distributed vulnerability. Finally, our classification of mapping patterns showed that one-to-many and many-to-many relationships were predominant, suggesting that cognitive deficits typically arise from disruptions across multiple, interconnected anatomical substrates rather than from focal lesions. These findings challenge modular accounts of brain organization and support a network-oriented perspective on lesion–symptom relationships.

Consistent with prior accounts of disconnection-related cognitive deficits in both stroke and glioma populations (Cochereau et al., 2020; Corbetta et al., 2015; Herbet et al., 2016b; Talozzi et al., 2023), we found that white matter disconnections were more strongly associated with behavioural impairments than cortical damage. This association was particularly pronounced in the left hemisphere, where disconnection patterns exhibited higher centrality and stronger connectivity with neuropsychological performance. These findings add to growing evidence that white matter disconnection serves as a key mechanistic driver of cognitive deficits, reflecting its integrative role in large-scale neurocognitive networks (Filley and Fields, 2016; Herbet and Duffau, 2020; Thiebaut De Schotten and Forkel, 2022). Notably, the persistence of this effect in low-grade glioma, despite the brain's capacity for functional reorganization in this brain condition (Herbet et al., 2024), suggests that disconnection is less amenable to compensatory mechanisms than cortical damage, highlighting its critical role in shaping cognitive outcomes (Herbet et al., 2016a).

In contrast, the right hemisphere exhibited more sparse and less structured associations between lesion data and cognitive outcomes, as evidenced by lower interlayer connectivity and a greater number of neuropsychological task nodes with near-zero to zero centrality. This asymmetry is consistent with previous findings suggesting that right hemisphere damage often leads to subtler, more domain-general impairments that are difficult to detect using conventional neuropsychological assessments (Knight et al., 1995; Stuss and Alexander, 2007). This may, in part, reflect limitations of standard clinical batteries, which tend to emphasize functions associated with the left hemisphere (language, verbal memory, and executive control), thereby underrepresenting the cognitive contributions of the right hemisphere. Supporting this, recent meta-analytic work introduced a ‘morphospace’ framework for brain–cognition organization (Pacella et al., 2024), demonstrating that functions linked to the right hemisphere are less predictable and more sparsely represented in the neuroimaging literature. Together, these findings point to a broader undercharacterization of right hemisphere function in both research and clinical practice, highlighting the need for more comprehensive assessment tools capable of capturing the full spectrum of human cognition.

Multilayer community detection analyses revealed reproducible and interpretable lesion–symptom motifs. In the left hemisphere, three principal communities were identified, each linking cognitive tasks with anatomically and functionally coherent cortical and subcortical regions. These communities corresponded specifically to executive control, language/verbal memory, and semantic processing domains. In contrast, the right hemisphere exhibited only one clearly interpretable lesion–symptom community, primarily associated with spatial cognition. Additional communities in the right hemisphere appeared fragmented and did not coherently span cognitive and anatomical layers, limiting their interpretability. The robustness of these mappings was further supported by dimensionality-reduction techniques, including t-SNE and UMAP embeddings. Beyond reinforcing established hemispheric specializations, these findings highlight the difficulty in isolating lesion–symptom relationships within small-scale anatomical and functional clusters, suggesting instead broader-scale associations between cognitive domains and lesion patterns.

Centrality analyses provided further differentiation of cognitive tasks based on the extent and strength of their structural dependencies. Tasks such as verbal fluency and immediate recall exhibited high degree and closeness centrality, suggesting that their performance depends on multiple, densely interconnected anatomical systems. In contrast, several tasks, especially within the right hemisphere, displayed minimal or zero centrality values. This indicates either more restricted structure–function relationships or potential limitations in the sensitivity of our measurement approaches.

Our classification of brain–behaviour mappings demonstrated that most neuropsychological tasks were associated with multiple anatomical structures, predominantly reflecting *many-to-many* and *one-to-many* mappings. This observation supports the conceptualization of cognitive functions as inherently multidetermined and distributed across interconnected neural networks, rather than localized within discrete brain regions, and is consistent with previous clinical-anatomical models suggesting multiples modes of structure-function association rather than strict unifocality (e.g. the “brain modes^”^; (Godefroy et al., 1998; Toba et al., 2020)). Notably, tasks exhibiting clear one-to-one anatomical mappings were relatively rare, underscoring the limited presence of strictly modular cognitive-anatomical relationships. Furthermore, our analyses identified specific white matter tracts, notably the superior fronto-striatal pathways and thalamic radiations, as recurrently linked to multiple cognitive tasks, highlighting their potential role as domain-general integrative hub-like structures. While these pathways do not singularly account for observed cognitive deficits, their repeated involvement underscores their integrative importance in supporting cognition across diverse domains.

Several limitations should be considered when interpreting the current findings. First, our analyses were conducted in a homogeneous clinical sample of patients with low-grade glioma, a rare disease characterized by gradual lesion progression and significant capacity for network reorganization. Consequently, the generalizability of our results to populations experiencing acute neurological conditions, such as ischemic stroke, remains limited. Future research directly comparing patient groups with slow-progressing versus sudden-onset lesions could provide valuable insights into how lesion timing shapes brain-behaviour relationships — an important yet scarcely investigated topic. Second, our estimation of structural disconnection relied on a normative white matter atlas. While diffuse low grade gliomas typically show slow infiltration growth with limited mass effect, especially the postoperative context examined here, interindividual variability in white matter anatomy and residual tract displacement cannot be entirely excluded. Such deviations from normative anatomy may have resulted in imprecise estimation of disconnections in some patients. Third, our multilayer network analyses depend on predefined network nodes and correlation-based thresholding; consequently, both the choice of cortical parcellation and the applied thresholds may influence sensitivity to subtle but clinically relevant lesion–symptom associations or introducing artefactual connectivity patterns with limited statistical power. Complementary analytical methods, including machine learning or probabilistic network modelling, could address these limitations and enhance the sensitivity, robustness, and predictive accuracy of clinical outcomes (e.g., individualized patient predictions); however, such techniques typically necessitate larger sample sizes for reliable implementation. Fourth, although our neuropsychological battery was comprehensive, it may not fully represent the complexity and diversity of cognitive functions supported by the right hemisphere, potentially contributing to the observed hemispheric asymmetries in interpretability. Fifth, although the neuropsychological layer captured longitudinal change through pre- to postoperative delta scores, the anatomical layers were derived from postoperative resection maps rather than repeated anatomical measurements across time. This choice was intentional, because in diffuse low-grade glioma, preoperative tumor infiltration cannot be directly equated with structural disconnection, as infiltrated pathways may remain at least partially functional before surgery. Lastly, longitudinal studies extending beyond the third-month post-surgical period would offer crucial insights into the temporal dynamics of network reorganization and recovery trajectories, clarifying how brain–behaviour mappings evolve over time.

In conclusion, our findings provide compelling evidence that brain–behaviour relationships in patients with low-grade glioma are best understood through a distributed, network-based framework rather than through traditional, localized lesion models. By integrating cortical lesions, structural disconnections, and cognitive outcomes within a multilayer network architecture, we demonstrate that cognitive impairments are primarily shaped by disruptions in structural connectivity - especially in domain-general white matter tracts that serve as integrative hubs across cognitive domains. This approach challenges long-standing assumptions of modularity in lesion–symptom mapping and offers a neurobiologically grounded account of how focal brain lesions give rise to diverse cognitive deficits. Beyond advancing theoretical models of structure–function relationships in lesion data, our results hold translational promise for the development of individualized, network-informed rehabilitation strategies. For instance, whereas most current rehabilitation strategies remain largely symptom-centric, integrating individual data into a unified neuropsychological – cortical damage –disconnection fingerprint, within which interdependencies across domains are jointly represented, may help elucidate the neural basis of deficit chronicity and recovery trajectories. Such a system-level perspective could, in turn, support the orientation of rehabilitation strategies toward the affected neural systems rather than toward isolated symptoms.

## CRediT authorship contribution statement

**Roxane Nave:** Writing – original draft, Visualization, Validation, Methodology, Formal analysis, Data curation, Conceptualization. **Sam Ng:** Methodology, Data curation. **Sylvie Moritz-Gasser:** Data curation. **Lorelei Berger:** Writing – review & editing, Data curation. **Emmanuel Mandonnet:** Writing – review & editing. **Hugues Duffau:** Writing – review & editing, Resources. **Guillaume Herbet:** Writing – original draft, Visualization, Validation, Supervision, Methodology, Formal analysis, Conceptualization.

## Funding

Roxane Nave is supported by the ANOF (Association de Neuro-oncologie Fonctionnelle).

## Declaration of competing interest

The authors declare that they have no known competing financial interests or personal relationships that could have appeared to influence the work reported in this paper.

## Data Availability

The authors do not have permission to share data.
