# Supplementary material for: Beyond one-to-one mappings: Modelling distributed lesion-symptom relationships with multilayer networks
Source: Neuroimage Clin. 2026 Aug 26;51:104052. doi: 10.1016/j.nicl.2026.104052 (PMC13551937; doi:10.1016/j.nicl.2026.104052)
Supplement: Supplementary file 1 [file mmc1.docx]

**Appendix A**: Graph-based measures and their interpretation in the present study

| **Analysis / Measure** | **Definition (as implemented here)** | **Clinical–anatomical interpretation** |
| --- | --- | --- |
| **Graph** | A mathematical representation of the dataset in which nodes correspond to neuropsychological tasks (NT, performance values), cortical parcels (CD, resection load), or white matter tracts (SD, structural disconnection severity), and edges represent Spearman correlations across patients. | Provides a unified framework to jointly model cognitive performance, cortical damage, and structural disconnection, and enabling the identification if recurring anatomo-functional motifs through graph-based analyses. |
| **Multilayer network** | A graph composed of three interconnected layers: neuropsychological tasks (NT), structural disconnections (SD), and cortical damage (CD), linked through a supra-adjacency matrix. | Enables the simultaneous integration of cortical and white matter effects on cognition, capturing complex cross-domains interactions that cannot be assessed when each modality is analyzed in isolation. |
| **Connectivity** | Pairwise Spearman correlation coefficients between nodes, computed within layers and across layers, reflecting co-occurrence patterns across patients. | Quantifies statistical dependencies between cognitive deficits and anatomical damage, providing an index of structure-function coupling and its modulation by tumour resection. |
| **Network density** | Proportion of suprathreshold edges (\|R\| ≥ threshold) relative to all possible connections in the network, computed after matching node counts across layers by subsampling. | Serves as a global index of interdependence between cognitive performance, cortical damage, and structural disconnection, allowing comparison of domain coupling (e.g., NT–SD vs. NT–CD) and hemispheric differences while controlling for network size. |
| **Community detection** | Data-driven identification of groups of nodes that are more strongly interconnected with each other than with the rest of the network. | Reveals coherent brain–behaviour motifs linking specific cognitive tasks with sets of cortical regions and white matter tracts that tend to be affected together. |
| **Louvain algorithm** | Modularity-optimizing community detection algorithm applied to weighted multilayer networks. | Allows stable identification of clinically interpretable subnetworks without prespecifying the number or composition of modules. |
| **Degree centrality (weighted)** | Sum of the weights of all edges directly connected to a node. | For NT nodes, reflects how many anatomical structures are directly associated with a given cognitive deficit, suggesting more focal or more extensive lesion–deficit relationships. |
| **Closeness centrality (weighted)** | Inverse of the average shortest-path distance from a node to all other nodes, with edge lengths defined as 1 − r. | For NT nodes, indexes sensitivity to distributed and indirect resection effects, capturing cumulative network-level vulnerability rather than focal damage alone. |
| **Spatial embedding (UMAP / t-SNE)** | Nonlinear dimensionality reduction methods projecting nodes into a low-dimensional space based on similarity of their multilayer connectivity profiles. | Provides geometric cross-validation of community structure and facilitates visual interpretation of brain–behaviour motifs across layers. |
| **Mapping pattern classification** | Categorization of NT–anatomy relationships into one-to-one, one-to-many, many-to-one, many-to-many, or none, based on suprathreshold correlations. | Enables explicit characterization of different lesion–behaviour mapping regimes, moving beyond one-to-one relationships toward distributed network dependence. |
