# Supplementary material for: Beyond one-to-one mappings: Modelling distributed lesion-symptom relationships with multilayer networks
Source: Neuroimage Clin. 2026 Aug 26;51:104052. doi: 10.1016/j.nicl.2026.104052 (PMC13551937; doi:10.1016/j.nicl.2026.104052)
Supplement: Supplementary file 2 [file mmc2.docx]

**Supplementary Information**

**-------**

**Beyond one-to-one mappings: Modeling distributed lesion-symptom relationships with multilayer graphs**

Roxane Nave^1^, Sam, Ng^1,2^, Sylvie Moritz-Gasser^1,2,3^, Lorelei Berger^3^, Emmanuel Mandonnet^4,5,6^, Hugues Duffau^1,2^, Guillaume Herbet^2,3,7,8^

^1^Institute of Functional Genomics, University of Montpellier, INSERM, CNRS, 141 rue de la Cardonille, 34091 Montpellier, France.

^2^Department of Neurosurgery, Gui de Chauliac Hospital, Montpellier University Medical Center, 80 Av Augustin Fliche, 34295 Montpellier, France.

^3^Praxiling laboratory, UMR 5267, CNRS, Paul Valéry – Montpellier 3 University, rue de Mende, 34090 Montpellier, France.

^4^Department of neurosurgery, Lariboisière Hospital, Paris, France

^5^Frontlab, Paris Brain Institute, CNRS UMR 7225, INSERM U1127, Paris, France

^6^Université de Paris Cité, Paris, France

^7^University of Montpellier, Department of Medicine, campus ADV, 641, avenue du Doyen Gaston Guiraud, 34090 Montpellier, France.

^8^Institut Universitaire de France, Paris.

1- **Supplementary Figures**

**Supplementary Figure 1:** Complete distribution of lesion metrics for (A) cortical damage (CD), quantified as the percentage of resection within cortical parcels defined by the Schaeffer’s atlas and (B) structural disconnection (SD), quantified as the percentage of streamlines disrupted within white matter tracts defined by the HCP-1065 white matter atlas. Bars indicate the mean percentage of damage across patients for each cortical parcel or white matter tract, while dots represent individual patient values. Shaded regions highlight the subset of cortical parcels and white-matter tracts retained for subsequent analyses, according to the inclusion criteria described in the Methods section.

**Supplementary Figure 2:** Mean neuropsychological performances across the five imputations for patients with left-hemisphere glioma (top panel) versus right-hemisphere glioma (bottom panel). For each neuropsychological task and each assessment time (pre- versus post-surgery) the mean and standard deviation were computed across imputations, reflecting variability across imputation iterations rather than inter-individual performance variability (see also Supplementary Table 3 and 4 for detailed descriptive statistics).

**Supplementary Figure 3:** Delta distributions by task and hemisphere across imputations. This figure shows violin distributions with individual datapoints of the delta values (i.e. difference between the post-surgery and the pre-surgery assessment) for each task, displayed separately for each hemisphere and across imputations. Task-wise *y*-axis scaling is shared across hemispheres to facilitate comparisons. Panels with no available data are left intentionally empty.

**Supplementary Figure 4:** (A) 2D between-layer projections for the left hemisphere. Subpanels illustrate specific cross-layer interactions: NT vs. SD (left), CD vs. SD (middle), and NT vs. CD (right). Nodes are colored by their respective layers, and edges represent significant positive correlations. (B) Correlation distribution for the left hemisphere. Histograms depict the distribution of R values for within-layer (left) and between-layer (right) correlations, with color coding for each layer combination. (C) 2D between-layer projections for the right hemisphere, structured identically to (A), illustrating cross-layer connectivity between NT and SD (left), CD and SD (middle), and NT and CD (right). (D) Correlation distribution for the right hemisphere, structured identically to (B), depicting the distribution of R values for within-layer and between-layer correlations across the three layers.

**Supplementary Figure 5:** Hierarchical structures of the three layers using dendrograms. Top, left hemisphere; bottom, right hemisphere. NT = Neuropsychological Task layer; SD = Structural Disconnection layer; CD = Cortical Damage (CD) layer.

**Supplementary Figure 6:** Results from node-controlled density analysis (see also Supplementary Table 14 for source data). In this figure are plotted mean ± SD density values controlled for node count.

2- **Supplementary Tables**

**Supplementary Table 1**: Glossary of acronyms and abbreviations

|  | **Acronym** | **Definition** |
| --- | --- | --- |
| **Neuropsychological tasks** | BELLS | Bells cancellation test |
|  | CODES | Coding subtest (processing speed; e.g. WAIS Coding) |
|  | DO80 | Dénomination Orale–80 test |
|  | DS_B | Digit Span Backward |
|  | DS_F | Digit Span Forward |
|  | FIG_C | Rey or Taylor Complex Figure Copy |
|  | FIG_R | Rey–Osterrieth or Taylor Complex Figure Recall |
|  | FLU_P | Phonemic (letter) verbal fluency |
|  | FLU_A | Semantic (category) verbal fluency |
|  | LB | Line Bisection |
|  | PPTT | Pyramids and Palm Trees Test |
|  | RLRI_E | Rappel libre–rappel indicé, Encoding |
|  | RLRI_FR | Rappel libre–rappel indicé, Free Recall |
|  | RLRI_TR | Rappel libre–rappel indicé, Total Recall |
|  | STROOP_D | Stroop Test, Denomination |
|  | STROOP_ID | Stroop interference (difference score) |
|  | STROOP_R | Stroop Test, Reading |
|  | TMT_A | Trail Making Test, Part A |
|  | TMT_BA | Trail Making Test, Part B–A (set-shifting cost) |
| **Cortical nodes** | ContA_PFCl_2 | Control Network, lateral prefrontal cortex node |
|  | ContB_Temp_2 | Control Network, temporal node |
|  | DMN_A_PFCd_2 | Default Mode Network (Anterior), dorsal prefrontal cortex node |
|  | DMN_B_PFCd_2 | Default Mode Network (Posterior), dorsal prefrontal cortex node |
|  | DMN_B_PFCv_2 | Default Mode Network (Posterior), ventral prefrontal cortex node |
|  | DMN_B_PFCv_3 | Default Mode Network (Posterior), ventral prefrontal cortex node |
|  | DMN_B_Temp_2 | Default Mode Network (Posterior), temporal node |
|  | LimbA_TP_2 | Limbic Network (Anterior), temporal pole node |
|  | LimbB_OFC_2 | Limbic Network (Posterior), orbitofrontal cortex node |
|  | SMB_Aud_2 | Somatomotor Network, auditory-related node |
|  | SVA_A_FrMed_2 | Salience/Ventral Attention Network (Anterior), medial frontal node |
|  | SVA_A_Ins_2 | Salience/Ventral Attention Network (Anterior), insula node |
|  | SVA_A_Ins_3 | Salience/Ventral Attention Network (Anterior), insula node |
|  | SVA_B_PFCmp_2 | Salience/Ventral Attention Network (Posterior), medial prefrontal cortex node |
|  | TP_2 | Temporo-Parietal Network, node 2 |
| **White matter tracts** | AC | Anterior Commissure |
|  | AF | Arcuate Fasciculus |
|  | CC_A | Corpus Callosum, Anterior |
|  | CC_MidA | Corpus Callosum, Mid-Anterior |
|  | CC_P | Corpus Callosum, Posterior |
|  | C_FP | Fronto–Parietal Cingulum |
|  | C_FPH | Fronto–Parietal–Hippocampal Cingulum |
|  | C_PH | Parahippocampal Cingulum |
|  | C_PHP | Parahippocampal–Parietal Cingulum |
|  | CPT_F | Corticopontine Tract, Frontal component |
|  | CS_A | Corticostriatal Tract, Anterior |
|  | CS_S | Corticostriatal Tract, Superior |
|  | EMC | Extreme Capsule |
|  | F | Fornix |
|  | FAT | Frontal Aslant Tract |
|  | IFOF | Inferior Fronto-Occipital Fasciculus |
|  | ILF | Inferior Longitudinal Fasciculus |
|  | MdLF | Middle Longitudinal Fasciculus |
|  | RST | Reticulospinal Tract |
| **Other** | CD | Cortical Damage |
|  | DLGG | Diffuse Low-Grade Glioma |
|  | DES | Direct Electrical Stimulation |
|  | LQT | Lesion Quantification Toolkit |
|  | MICE | Multiple Imputation by Chained Equations |
|  | MNI | Montreal Neurological Institute |
|  | MRI | Magnetic Resonance Imaging |
|  | NMI | Normal Mutual Information |
|  | NT | Neuropsychological Task |
|  | PCA | Principal Component Analysis |
|  | PMM | Predictive Mean Matching |
|  | SD | Structural Disconnection |
|  | SPM | Statistical Parametric Mapping |
|  | t-SNE | t-distributed Stochastic Neighbor Embedding |
|  | UMAP | Uniform Manifold Approximation and Projection |

**Supplementary Table 2**: Battery of cognitive tasks used in the study

| **Domain** | **Task** | **Processes** | **Task description** |
| --- | --- | --- | --- |
| **Executive Functions** | Trail Making Test^1^ Part B minus part A (**TMT_B-A**) | Cognitive flexibility; divided attention; visuomotor speed | Participants are instructed to alternately switch between numbers and letters. connecting them in ascending order as rapidly as possible |
|  | STROOP^1^ Interference (**STROOP_ID**) | Inhibition of competing responses; selective attention; cognitive flexibility | In the incongruent condition. colour words are printed in an inconsistent colour ink. Participants are instructed to name the colour of the ink rather than reading the actual word. |
|  | Forward span. Backward span **(DS_F/DS_B**) | Immediate memory recall; verbal memory span; working memory | Participants are instructed to immediately recall a series of digits provided orally by the clinician. either in a forward or backward sequence. |
|  | Phonological fluency^1^ (**FLU_P**) | Cognitive flexibility; phonological thought | Participants are asked to generate words starting with the letter 'P' within a time limit of 2 minutes. |
|  | Categorical fluency^1^ (**FLU_A**) | Cognitive flexibility; semantic memory | Participants are asked to generate words belonging to the category ‘animals’ within a time limit of 2 minutes. |
| **Psychomotor Speed and Attention** | Trail Making Test (**TMT_A**) | Visuomotor speed; attention | Participants are requested to connect numbers in ascending order as swiftly as possible. |
|  | WAIS-IV Coding subtest^2^ (**CODES**) | Psychomotor speed; visual scanning; sustained attention; visuomotor coordination | Participants transcribe symbols corresponding to numbers using a reference key as quickly and accurately as possible with a fixed time limit. |
|  | STROOP naming (**STROOP_D**) | Psycho-motor speed; selective attention; | In the congruent condition. participants are tasked with naming colour patches (congruous stimuli) as quickly as possible. |
|  | STROOP reading (**STROOP_R**) | Psycho-motor speed; selective attention | In the congruent condition. participants are instructed to read names of colors as quickly as possible. |
| **Visuospatial function** | Bell Cancellation test (**BELLS**) ^6^ | Visual exploration; spatial attention | This is a target cancellation task where participants are required to encircle bells among distractors. |
|  | Line bisection (**LB**) | Visuospatial attention, spatial bias. | LB: Participants have to mark the center of a horizontal line of 8cm. |
|  | Rey-Osterreich or Taylor complex figure^3-5^ - copy (**FIG_C**) | Visuo-constructive abilities; visuospatial planning; organizational strategies | Participants are instructed to copy a complex geometric figure as accurately as possible, providing an index of visuo-construction and planning abilities. |
| **Language/semantics** | Dénomination Orale^7^ -Picture naming (**DO80**) | Picture naming (lexical retrieval) | Patients are instructed to name 80 black and white pictures |
|  | Pyramids and Palm Trees Test (**PPTT**) | Nonverbal semantic access; semantic memory | A single target picture is presented alongside two other pictures. and the patient is tasked with matching one of the two with the target picture based on a semantic link |
| **Verbal Episodic Memory** | Rappel Libre/Rappel Indicé Task^9^, Encoding (**RLRI_E**) | Verbal learning; verbal episodic memory | Participants are tasked with learning a list of 16 words through a structured process. This includes three immediate successive recalls. divided into two parts: the first involves free recall of the items within a limited time. followed by a total recall where categorical cues are provided for non-retrieved items from the free recall part. Additionally, there is one delayed recall after 20 minutes. followed by a total delayed recall |
|  | Free Recall (Trials 1-3 and delayed) (**RLRI_FR**) |  |  |
|  | Total Recall (Trials 1-3 and delayed) (**RLRI_TR**) |  |  |
| **Non-Verbal Episodic Memory** | Rey-Osterrieth/Taylor complex figure^3-5^ - Recall (**FIG_R**) | Visual memory; visuo-spatial integration; planning | Participants are instructed to reproduce a figure from memory without prior forewarning. both immediately (after 3 minutes) and following a delay (after 30 minutes) |

1. Godefroy O. Fonctions Exécutives et Pathologies Neurologiques et Psychiatriques : Évaluation En Pratique Clinique. Groupe de Boeck; 2008.
2. Wechsler D. Wechsler adult intelligence scale–Fourth Edition (WAIS–IV). San Antonio. TX: NCS Pearson.2008;22(498):1.
3. Rey A. L’examen psychologique dans les cas d’encéphalopathie traumatique. (Les problèmes.). Archives de psychologie. Published online 1941.
4. Osterrieth PA. Le test de copie d’une figure complexe ; contribution à l’étude de la perception et de la mémoire. Archives de Psychologie. Published online 1944.
5. Taylor LB. Localisation of cerebral lesions by psychological testing. Neurosurgery. 1969;16(CN_suppl_1):269-287.
6. Gauthier L. Dehaut F. Joanette Y. The bells test: a quantitative and qualitative test for visual neglect. International journal of clinical neuropsychology. 1989;11(2):49-54.
7. Metz-Lutz MN. Standardisation d’un test de dénomination orale: contrôle des effets de l’âge, du sexe et du niveau de scolarité chez des sujets adultes normaux. Rev Neuropsychol. 1991;1:73-95.
8. Howard D. Patterson K. The Pyramids and Palm Trees Test: A Test of Semantic Access from Words and Pictures. Pearson Assessment; 1992.
9. Van der Linden M. Coyette F. Poitrenaud J. Kalafat M. Calicis F. Wyns C. L’épreuve de rappel libre/rappel indicé à 16 items (RL/RI-16)[Memory test assessment. Free recall with an index of 16 items (RL/RI-16)]. L’évaluation des troubles de la mémoire Présentation de quatre tests de mémoire épisodique (avec leur étalonnage). Published online 2004

**Supplementary Table 3**: Sociodemographic variables

| **Task** |  | **Pre-surgery data** | | | | | **Post-surgery data** | | | | | **Total data** | | |  | | |  |
| --- | --- | --- | --- | --- | --- | --- | --- | --- | --- | --- | --- | --- | --- | --- | --- | --- | --- | --- |
|  | **n** | **mean** | **SD** | **range** | **MD (n)** | **MD (%)** | **mean** | **SD** | **range** | **MD (n)** | **MD (%)** | **MD (n)** | **MD (%)** | |  | |  |  |
| **Bell test (omissions)^b^** | 126 | 2.12 | 2.47 | [0-10] | 8 | 6.35 | 1.82 | 1.89 | [0-8] | 6 | 4.76 | 14 | 5.55 | |  | |  |  |
| **Codes** | 252 | 71.69 | 14.49 | [20-111] | 47 | 18.65 | 71.41 | 14.93 | [25-105] | 49 | 19.44 | 96 | 19.05 | |  | |  |  |
| **DO80^a^** | 126 | 78.49 | 3.07 | [50-80] | 2 | 1.59 | 78.49 | 4.73 | [48-80] | 10 | 7.94 | 12 | 4.76 | |  | |  |  |
| **Digit Span Forward** | 252 | 6.30 | 1.18 | [0-9] | 41 | 16.27 | 6.42 | 1.21 | [0-9] | 36 | 14.29 | 77 | 15.28 | |  | |  |  |
| **Digit Span Backward** | 252 | 4.95 | 1.18 | [2-8] | 42 | 16.67 | 5.02 | 1.21 | [2-8] | 37 | 14.68 | 79 | 15.67 | |  | |  |  |
| **Figure Rey/Taylor (Copy)^b^** | 126 | 35.60 | 0.91 | [31-36] | 22 | 17.46 | 35.45 | 1.23 | [27-36] | 28 | 22.22 | 50 | 19.84 | |  | |  |  |
| **Figure Rey/Taylor (Recall)^b^** | 126 | 23.17 | 6.73 | [9-35] | 38 | 30.16 | 24.93 | 5.50 | [10-36] | 44 | 34.92 | 82 | 32.54 | |  | |  |  |
| **Fluency (semantic)** | 252 | 32.37 | 9.54 | [9-67] | 5 | 1.98 | 30.85 | 10.20 | [5-62] | 5 | 1.98 | 10 | 1.99 | |  | |  |  |
| **Fluency (phonological)** | 252 | 22.38 | 7.66 | [5-54] | 3 | 1.19 | 21.98 | 8.46 | [0-52] | 3 | 1.19 | 6 | 1.19 | |  | |  |  |
| **Line bissection^b^** | 126 | -1.04 | 2.49 | [-7.6-6] | 20 | 15.87 | -0.82 | 2.82 | [-6.7-8.8] | 19 | 15.08 | 39 | 15.48 | |  | |  |  |
| **PPTT^a^** | 126 | 48.82 | 2.42 | [40-52] | 3 | 2.38 | 49.29 | 2.32 | [39-52] | 10 | 7.94 | 13 | 5.16 | |  | |  |  |
| **RL-RI (encoding)** | 252 | 15.68 | 0.65 | [13-16] | 70 | 27.78 | 15.51 | 1.29 | [3-16] | 75 | 29.76 | 145 | 28.77 | |  | |  |  |
| **RL-RI (free recall)** | 252 | 31.94 | 6.50 | [0-46] | 70 | 27.78 | 32.77 | 7.12 | [0-47] | 88 | 34.92 | 158 | 31.35 | |  | |  |  |
| **RL-RI (total recall)** | 252 | 46.00 | 3.31 | [26-48] | 71 | 28.17 | 45.66 | 3.75 | [25-48] | 89 | 35.32 | 160 | 31.75 | |  | |  |  |
| **Stroop I -D** | 252 | 43.57 | 21.18 | [1-196] | 22 | 8.73 | 41.57 | 22.54 | [1-178] | 24 | 9.52 | 46 | 9.13 | |  | |  |  |
| **Stroop naming** | 252 | 59.37 | 11.00 | [34-100] | 25 | 9.92 | 62.72 | 17.69 | [38-167] | 25 | 9.92 | 50 | 9.92 | |  | |  |  |
| **Stroop reading** | 252 | 43.52 | 8.55 | [27-97] | 26 | 10.32 | 46.14 | 11.70 | [30-160] | 26 | 10.32 | 52 | 10.32 | |  | |  |  |
| **TMT-A** | 252 | 26.81 | 9.30 | [13-64] | 22 | 8.73 | 28.37 | 13.35 | [12-163] | 19 | 7.54 | 41 | 8.14 | |  | |  |  |
| **TMT B-A** | 252 | 37.72 | 18.48 | [5-117] | 20 | 7.94 | 38.79 | 27.43 | [3-237] | 17 | 6.75 | 37 | 7.34 | |  | |  |  |
| Note: MD = Missing Data; PPTT = Pyramid and Palm Trees Test; RL-RL = rappel libre-rappel indicé; TMT = Trail Making Test (parts A and B). ^a^Only patients with left-sided tumours. ^b^Only patients with right-sided tumours. Missing data were mainly task-specific and reflected clinical and organizational constraints. Episodic memory tasks (e.g., RL–RI) were more frequently incomplete because they are cognitively demanding, involve multiple recall phases, and are time-consuming, making them particularly sensitive to fatigue, distress, and perioperative constraints. | | | | | | | | | | | | | | | |  | | |
|  | | | | | | | | |  |  |  |  | |  | |  | | |

**Supplementary Table 4:** Across imputation comparisons using non parametric ANOVA for repeated measures – Left hemisphere

| **Subtasks** | **ANOVA** | | **1** | | | **2** | | | **3** | | | **4** | | | **5** | | |
| --- | --- | --- | --- | --- | --- | --- | --- | --- | --- | --- | --- | --- | --- | --- | --- | --- | --- |
|  | ***Χ^2^*** | ***P*** | **mean** | **SD** | **med** | **mean** | **SD** | **med** | **mean** | **SD** | **med** | **mean** | **SD** | **med** | **mean** | **SD** | **med** |
| CODES_Pre | 3.12 | 0.54 | 71.2 | 12.8 | 72.0 | 70.3 | 13.1 | 70.0 | 70.7 | 13.2 | 70.0 | 71.5 | 13.2 | 73.0 | 70.9 | 13.3 | 70.0 |
| CODES_Post | 7.55 | 0.11 | 70.4 | 13.9 | 70.0 | 70.6 | 14.6 | 71.0 | 69.9 | 14.5 | 70.0 | 71.4 | 14.5 | 71.0 | 69.9 | 14.0 | 70.0 |
| DO80_Pre | 3.00 | 0.56 | 78.6 | 3.1 | 79.0 | 78.6 | 3.1 | 79.0 | 78.6 | 3.1 | 79.0 | 78.6 | 3.1 | 79.0 | 78.6 | 3.1 | 79.0 |
| DO80_Post | 9.17 | 0.06 | 77.7 | 4.6 | 79.0 | 77.6 | 4.6 | 79.0 | 77.7 | 4.6 | 79.0 | 77.7 | 4.6 | 79.0 | 77.5 | 4.7 | 79.0 |
| DS_B_Pre | 1.56 | 0.82 | 4.8 | 1.1 | 5.0 | 4.8 | 1.2 | 5.0 | 4.8 | 1.1 | 5.0 | 4.8 | 1.2 | 5.0 | 4.7 | 1.1 | 5.0 |
| DS_B_Post | 3.02 | 0.55 | 4.8 | 1.2 | 5.0 | 4.8 | 1.2 | 5.0 | 4.8 | 1.2 | 5.0 | 4.8 | 1.2 | 5.0 | 4.8 | 1.2 | 5.0 |
| DS_F_Pre | 0.16 | 1.00 | 6.3 | 1.1 | 6.0 | 6.3 | 1.2 | 6.0 | 6.3 | 1.1 | 6.0 | 6.3 | 1.1 | 6.0 | 6.3 | 1.1 | 6.0 |
| DS_F_Post | 7.18 | 0.13 | 6.4 | 1.1 | 6.0 | 6.3 | 1.0 | 6.0 | 6.3 | 1.1 | 6.0 | 6.4 | 1.1 | 6.0 | 6.4 | 1.1 | 6.0 |
| FLU_A_Pre | 4.00 | 0.41 | 31.9 | 9.4 | 31.0 | 32.2 | 9.5 | 31.0 | 31.9 | 9.3 | 31.0 | 32.0 | 9.3 | 31.0 | 32.0 | 9.3 | 31.0 |
| FLU_A_Post | 4.00 | 0.41 | 29.8 | 10.6 | 30.0 | 29.9 | 10.6 | 30.0 | 29.8 | 10.6 | 30.0 | 29.8 | 10.6 | 30.0 | 29.9 | 10.6 | 30.0 |
| FLU_P_Pre | 4.00 | 0.41 | 22.2 | 8.1 | 21.0 | 22.2 | 8.1 | 21.0 | 22.4 | 8.4 | 21.0 | 22.1 | 8.1 | 21.0 | 22.2 | 8.1 | 21.0 |
| FLU_P_Post | 4.00 | 0.41 | 20.8 | 8.5 | 20.0 | 20.8 | 8.5 | 20.0 | 20.8 | 8.5 | 20.0 | 20.8 | 8.5 | 20.0 | 20.8 | 8.5 | 20.0 |
| PPTT_Pre | 3.27 | 0.51 | 48.8 | 2.5 | 49.0 | 48.8 | 2.5 | 49.0 | 48.8 | 2.4 | 49.0 | 48.8 | 2.4 | 49.0 | 48.9 | 2.4 | 49.0 |
| PPTT_Post | 1.45 | 0.84 | 49.3 | 2.3 | 50.0 | 49.2 | 2.5 | 50.0 | 49.3 | 2.3 | 50.0 | 49.3 | 2.3 | 50.0 | 49.3 | 2.3 | 50.0 |
| RLRI_E_Pre | 2.59 | 0.63 | 15.7 | 0.6 | 16.0 | 15.7 | 0.6 | 16.0 | 15.7 | 0.6 | 16.0 | 15.7 | 0.6 | 16.0 | 15.7 | 0.6 | 16.0 |
| RLRI_E_Post | 45.51 | <0.001 | 15.2 | 1.6 | 16.0 | 15.7 | 0.6 | 16.0 | 15.2 | 1.5 | 16.0 | 15.2 | 1.5 | 16.0 | 15.3 | 1.5 | 16.0 |
| RLRI_FR_Pre | 2.81 | 0.59 | 31.8 | 6.4 | 33.0 | 32.0 | 6.6 | 33.0 | 31.9 | 6.4 | 33.0 | 32.1 | 6.3 | 33.0 | 31.8 | 6.9 | 33.0 |
| RLRI_FR_Post | 6.53 | 0.16 | 31.3 | 7.8 | 31.0 | 30.7 | 7.5 | 31.0 | 31.9 | 7.3 | 33.0 | 31.6 | 7.8 | 32.0 | 31.9 | 6.9 | 32.0 |
| RLRI_TR_Pre | 7.11 | 0.13 | 45.8 | 3.9 | 47.0 | 45.7 | 3.7 | 47.0 | 45.7 | 4.3 | 47.0 | 45.0 | 5.0 | 47.0 | 45.6 | 4.4 | 47.0 |
| RLRI_TR_Post | 4.50 | 0.34 | 44.7 | 4.3 | 46.0 | 44.9 | 4.0 | 47.0 | 44.9 | 4.7 | 47.0 | 45.0 | 4.0 | 47.0 | 44.4 | 5.1 | 46.0 |
| STROOP_D_Pre | 0.75 | 0.95 | 60.6 | 10.8 | 58.0 | 60.1 | 10.2 | 58.0 | 60.3 | 10.1 | 58.0 | 60.4 | 10.2 | 58.0 | 60.0 | 10.8 | 57.0 |
| STROOP_D_Post | 4.95 | 0.29 | 65.3 | 20.5 | 60.0 | 65.3 | 20.3 | 60.0 | 65.5 | 21.0 | 60.0 | 66.3 | 20.5 | 60.0 | 65.7 | 20.3 | 61.0 |
| STROOP_ID_Pre | 2.24 | 0.69 | 42.6 | 24.0 | 37.0 | 43.2 | 24.1 | 38.0 | 44.3 | 27.6 | 37.0 | 45.2 | 28.2 | 38.0 | 43.4 | 24.3 | 37.0 |
| STROOP_ID_Post | 1.23 | 0.87 | 43.8 | 29.4 | 38.0 | 42.3 | 28.5 | 36.0 | 44.0 | 31.0 | 36.0 | 43.1 | 28.6 | 36.0 | 42.7 | 26.5 | 38.0 |
| STROOP_R_Pre | 6.38 | 0.17 | 43.3 | 6.2 | 42.0 | 43.1 | 6.2 | 42.0 | 43.8 | 6.6 | 42.0 | 43.8 | 6.7 | 42.0 | 43.6 | 6.7 | 42.0 |
| STROOP_R_Post | 3.45 | 0.49 | 47.6 | 13.2 | 45.0 | 47.6 | 13.3 | 45.0 | 47.2 | 13.6 | 45.0 | 47.1 | 13.3 | 45.0 | 47.3 | 13.4 | 45.0 |
| TMT_A_Pre | 1.87 | 0.76 | 27.6 | 9.1 | 26.0 | 27.8 | 9.2 | 26.0 | 27.7 | 9.0 | 26.0 | 27.6 | 9.0 | 26.0 | 27.4 | 9.0 | 26.0 |
| TMT_A_Post | 2.19 | 0.70 | 29.5 | 15.3 | 26.0 | 29.5 | 15.4 | 26.0 | 29.5 | 15.3 | 27.0 | 29.8 | 15.3 | 27.0 | 29.3 | 15.3 | 26.0 |
| TMT_BA_Pre | 3.85 | 0.43 | 40.1 | 19.4 | 36.0 | 41.7 | 20.4 | 37.0 | 40.5 | 19.5 | 36.0 | 39.9 | 19.3 | 35.0 | 41.2 | 20.3 | 36.0 |
| TMT_BA_Post | 3.08 | 0.55 | 44.8 | 30.0 | 37.0 | 48.0 | 35.3 | 39.0 | 47.1 | 35.0 | 39.0 | 46.8 | 34.4 | 39.0 | 45.5 | 30.6 | 38.0 |

Note: A Friedman ANOVA. a non-parametric repeated measures test. was performed to assess whether imputed data were equivalent across imputations and assessment times. The analysis revealed a significant difference for only one task at one assessment time (RLRI_E_Post). However, despite this significance. the median values were identical across imputations. and the means were very similar.

**Supplementary Table 5:** Across imputation comparisons using non parametric ANOVA for repeated measures – Right hemisphere

| **Subtasks** | **ANOVA** | | **1** | | | **2** | | | **3** | | | **4** | | | **5** | | |
| --- | --- | --- | --- | --- | --- | --- | --- | --- | --- | --- | --- | --- | --- | --- | --- | --- | --- |
|  | ***Χ^2^*** | ***P*** | **mean** | **SD** | **med** | **mean** | **SD** | **med** | **mean** | **SD** | **med** | **mean** | **SD** | **med** | **mean** | **SD** | **med** |
| BELLS_Pre | 0.97 | 0.91 | 2.11 | 2.48 | 1 | 2.09 | 2.42 | 1 | 2.08 | 2.42 | 1 | 2.07 | 2.42 | 1 | 2.09 | 2.46 | 1 |
| BELLS_Postop | 2.68 | 0.61 | 1.85 | 1.86 | 2 | 1.85 | 1.94 | 1 | 1.89 | 1.89 | 2 | 1.83 | 1.88 | 1 | 1.86 | 1.89 | 2 |
| CODES_Pre | 5.27 | 0.26 | 73.1 | 14.7 | 74 | 73.6 | 15.1 | 74 | 72.7 | 15.4 | 73 | 72.7 | 15.1 | 73 | 73.7 | 15.7 | 74 |
| CODES_Postop | 2.03 | 0.73 | 72.1 | 15.8 | 74 | 72.2 | 15.4 | 74 | 72.6 | 15.2 | 74 | 71.4 | 15.6 | 74 | 72.6 | 15.7 | 74 |
| DS_B_Pre | 3.65 | 0.46 | 5.27 | 1.3 | 5 | 5.14 | 1.23 | 5 | 5.13 | 1.21 | 5 | 5.16 | 1.27 | 5 | 5.14 | 1.18 | 5 |
| DS_B_Postop | 7.90 | 0.10 | 5.44 | 1.24 | 5 | 5.38 | 1.22 | 5 | 5.28 | 1.21 | 5 | 5.34 | 1.18 | 5 | 5.38 | 1.21 | 5 |
| DS_F_Pre | 1.72 | 0.79 | 6.33 | 1.08 | 6 | 6.35 | 1.06 | 6 | 6.33 | 1.07 | 6 | 6.36 | 1.06 | 6 | 6.37 | 1.02 | 6 |
| DS_F_Postop | 7.30 | 0.12 | 6.72 | 1.17 | 7 | 6.62 | 1.15 | 7 | 6.59 | 1.14 | 7 | 6.63 | 1.15 | 7 | 6.6 | 1.16 | 7 |
| FIG_C_Pre | 1.90 | 0.75 | 35.6 | 0.87 | 36 | 35.6 | 0.89 | 36 | 35.6 | 0.97 | 36 | 35.6 | 0.89 | 36 | 35.6 | 0.87 | 36 |
| FIG_C_Postop | 1.13 | 0.89 | 35.4 | 1.36 | 36 | 35.5 | 1.15 | 36 | 35.5 | 1.15 | 36 | 35.5 | 1.15 | 36 | 35.4 | 1.37 | 36 |
| FIG_R_Pre | 3.52 | 0.48 | 22.8 | 7.07 | 21.5 | 23 | 7.05 | 22.5 | 22.9 | 6.68 | 21.5 | 23.7 | 6.47 | 24 | 22.7 | 7.02 | 21.5 |
| FIG_R_Postop | 14.31 | 0.01 | 24.8 | 5.41 | 25.5 | 25 | 5.59 | 25.5 | 24.7 | 5.58 | 25 | 23.9 | 5.78 | 24 | 23.8 | 6.07 | 24 |
| FLU_A_Pre | 9.19 | 0.06 | 32.7 | 9.81 | 32 | 32.7 | 9.79 | 32 | 32.7 | 9.69 | 32 | 33.2 | 10 | 32 | 32.8 | 9.77 | 32 |
| FLU_A_Postop | 0.62 | 0.96 | 31.9 | 9.81 | 31 | 31.9 | 9.68 | 31 | 31.8 | 9.64 | 31 | 31.9 | 9.64 | 31 | 31.8 | 9.69 | 31 |
| FLU_P_Pre | 1.74 | 0.78 | 22.5 | 7.21 | 22 | 22.4 | 7.24 | 22 | 22.5 | 7.21 | 22 | 22.4 | 7.24 | 22 | 22.6 | 7.26 | 22 |
| FLU_P_Postop | 6.26 | 0.18 | 23.1 | 8.28 | 22 | 23 | 8.31 | 22 | 23.3 | 8.4 | 22 | 23 | 8.32 | 22 | 23.1 | 8.27 | 22 |
| LB_Pre | 7.50 | 0.11 | -1.24 | 2.45 | -1.2 | -1.08 | 2.37 | -1.1 | -1.19 | 2.38 | -1.2 | -1.06 | 2.37 | -1.2 | -1.09 | 2.37 | -1.1 |
| LB_Postop | 3.54 | 0.47 | -1.03 | 2.74 | -1.2 | -0.89 | 2.77 | -1.1 | -1.02 | 2.75 | -1.3 | -0.99 | 2.73 | -1.2 | -1.02 | 2.69 | -1.3 |
| RLRI_E_Pre | 3.36 | 0.50 | 15.7 | 0.61 | 16 | 15.7 | 0.63 | 16 | 15.8 | 0.56 | 16 | 15.7 | 0.67 | 16 | 15.7 | 0.6 | 16 |
| RLRI_E_Postop | 5.58 | 0.23 | 15.8 | 0.43 | 16 | 15.9 | 0.39 | 16 | 15.9 | 0.46 | 16 | 15.8 | 0.49 | 16 | 15.9 | 0.46 | 16 |
| RLRI_FR_Pre | 0.43 | 0.98 | 32.5 | 5.56 | 33 | 32.4 | 5.68 | 33 | 32.7 | 5.69 | 33 | 32.5 | 6.09 | 33 | 32.5 | 5.67 | 33 |
| RLRI_FR_Postop | 1.32 | 0.86 | 34.9 | 5.72 | 36 | 34.8 | 5.56 | 36 | 34.9 | 5.7 | 36 | 35 | 5.69 | 36 | 34.6 | 5.87 | 36 |
| RLRI_TR_Pre | 12.03 | 0.02 | 46.3 | 2.55 | 47 | 46.5 | 2.27 | 47 | 46.4 | 2.36 | 47 | 46.2 | 2.36 | 47 | 46.4 | 2.25 | 47 |
| RLRI_TR_Postop | 3.85 | 0.43 | 46.4 | 2.69 | 47 | 46.6 | 2.29 | 48 | 46.5 | 2.55 | 47 | 46.3 | 3.12 | 48 | 46.6 | 2.42 | 48 |
| STROOP_D_Pre | 12.20 | 0.02 | 59 | 11.3 | 58 | 58.3 | 11.2 | 57 | 58.6 | 11.2 | 57 | 58.1 | 11.3 | 57 | 58.2 | 11.3 | 57 |
| STROOP_D_Postop | 3.38 | 0.50 | 59.8 | 14.1 | 57 | 59 | 12.7 | 57 | 58.9 | 12.7 | 57 | 58.8 | 12.8 | 57 | 59.2 | 12.9 | 57 |
| STROOP_ID_Pre | 1.76 | 0.78 | 43.3 | 16.6 | 41 | 43.9 | 16.9 | 42 | 43.8 | 17.1 | 42 | 43.6 | 16.6 | 42 | 43.5 | 16.8 | 42 |
| STROOP_ID_Postop | 6.12 | 0.19 | 39.6 | 17.2 | 36 | 40.2 | 17.2 | 37 | 39.5 | 17.2 | 36 | 39.7 | 17.2 | 36 | 39.8 | 17.1 | 36 |
| STROOP_R_Pre | 2.95 | 0.57 | 43.2 | 10 | 41 | 43.3 | 9.92 | 42 | 43.4 | 9.98 | 41 | 43.6 | 10 | 42 | 43.5 | 10.3 | 41 |
| STROOP_R_Postop | 3.94 | 0.41 | 44.5 | 8.97 | 43 | 44.9 | 9.02 | 44 | 44.5 | 9.09 | 43 | 45 | 9.52 | 43 | 44.5 | 8.96 | 43 |
| TMT_A_Pre | 6.48 | 0.17 | 25.4 | 9.14 | 23 | 25.7 | 9.18 | 24 | 26 | 9.61 | 24 | 25.4 | 9.07 | 23 | 25.3 | 9.21 | 23 |
| TMT_A_Postop | 4.74 | 0.32 | 26.8 | 10.1 | 24 | 26.8 | 10.3 | 24 | 26.8 | 10.2 | 24 | 27.2 | 10.4 | 24 | 27.3 | 10.5 | 24 |
| TMT_BA_Pre | 3.59 | 0.46 | 35.2 | 16.7 | 34 | 35.3 | 16.4 | 35 | 35.1 | 16.3 | 34 | 35.6 | 16.7 | 35 | 35.3 | 16.4 | 35 |
| TMT_BA_Postop | 4.54 | 0.34 | 33.8 | 23.1 | 29 | 32.7 | 22.3 | 29 | 33.1 | 22 | 29 | 33 | 22 | 29 | 32.8 | 22.1 | 28 |

Note: A Friedman ANOVA: a non-parametric repeated measures test. was performed to assess whether imputed data were equivalent across imputations and assessment times. The analysis revealed a significant difference for only three tasks at one assessment time (Fig_R_Post. RLRI_TR_Pre and Stroop_D_Pre). However, despite this significance. the median values and the means were very similar across imputations.

**Supplementary Table 6:** Delta measure computations

To specifically assess the effects of neurosurgery on neuropsychological performance, a delta score (Δ%) was computed for each task by comparing pre- and post-operative assessments. The formula was adapted for each test to ensure that higher Δ% values consistently reflected greater cognitive impairment, thereby facilitating interpretation of positive associations between structural damage and performance decline.

**Supplementary Table 7**: Statistical comparisons between the pre- and postoperative assessments across imputations for patients with a left-sided tumor

| **Task** | **N** | **I_ID** | **pre-surgery** | | | **post-surgery** | | | **W** | ***p*** | ***p*FDR** |
| --- | --- | --- | --- | --- | --- | --- | --- | --- | --- | --- | --- |
|  |  |  | **mean** | **SD** | **Rate** | **mean** | **SD** | **Rate** |  |  |  |
| CODES | 91 | CC | 71.1 | 13.35 | 4.4 | 69.65 | 14.54 | 7.69 | 1524 | 0.183 | 0.318 |
| CODES | 126 | i1 | 71.22 | 12.79 | 3.97 | 70.34 | 13.8 | 5.56 | 3311 | 0.403 | 0.516 |
| CODES | 126 | i2 | 70.34 | 13.06 | 5.56 | 70.53 | 14.58 | 6.35 | 3557.5 | 0.849 | 0.923 |
| CODES | 126 | i3 | 70.75 | 13.2 | 3.18 | 69.87 | 14.42 | 6.35 | 3167 | 0.285 | 0.396 |
| CODES | 126 | i4 | 71.52 | 13.13 | 3.97 | 71.39 | 14.44 | 6.35 | 3472.5 | 0.68 | 0.809 |
| CODES | 126 | i5 | 70.96 | 13.24 | 4.77 | 69.92 | 13.99 | 5.56 | 3161.5 | 0.22 | 0.337 |
| DO80 | 116 | CC | 78.53 | 3.16 | 3.45 | 77.55 | 4.73 | 10.34 | 920.5 | 0.007 | 0.029 |
| DO80 | 126 | i1 | 78.61 | 3.05 | 3.18 | 77.68 | 4.57 | 9.52 | 1070 | 0.005 | 0.013 |
| DO80 | 126 | i2 | 78.6 | 3.05 | 3.18 | 77.59 | 4.56 | 9.52 | 1124 | 0.002 | 0.008 |
| DO80 | 126 | i3 | 78.61 | 3.05 | 3.18 | 77.69 | 4.57 | 9.52 | 1047.5 | 0.005 | 0.013 |
| DO80 | 126 | i4 | 78.61 | 3.05 | 3.18 | 77.71 | 4.57 | 9.52 | 1075.5 | 0.008 | 0.02 |
| DO80 | 126 | i5 | 78.6 | 3.05 | 3.18 | 77.48 | 4.7 | 11.11 | 957.5 | 0.001 | 0.005 |
| DS_B | 102 | CC | 4.76 | 1.11 | 9.8 | 4.74 | 1.21 | 16.67 | 1354.5 | 0.851 | 0.906 |
| DS_B | 126 | i1 | 4.79 | 1.08 | 9.52 | 4.8 | 1.17 | 13.49 | 1931.5 | 0.908 | 0.946 |
| DS_B | 126 | i2 | 4.77 | 1.15 | 9.52 | 4.79 | 1.18 | 14.29 | 1970 | 0.889 | 0.939 |
| DS_B | 126 | i3 | 4.83 | 1.12 | 7.94 | 4.83 | 1.21 | 14.29 | 2125 | 0.955 | 0.973 |
| DS_B | 126 | i4 | 4.79 | 1.2 | 11.11 | 4.8 | 1.22 | 15.08 | 2322 | 0.982 | 0.982 |
| DS_B | 126 | i5 | 4.75 | 1.1 | 11.11 | 4.77 | 1.2 | 15.08 | 1984.5 | 0.79 | 0.884 |
| DS_F | 102 | CC | 6.28 | 1.14 | 6.86 | 6.35 | 1.12 | 4.9 | 759.5 | 0.579 | 0.659 |
| DS_F | 126 | i1 | 6.32 | 1.11 | 7.14 | 6.44 | 1.09 | 3.97 | 1081.5 | 0.24 | 0.34 |
| DS_F | 126 | i2 | 6.32 | 1.15 | 6.35 | 6.35 | 1.04 | 3.97 | 1372 | 0.77 | 0.875 |
| DS_F | 126 | i3 | 6.33 | 1.14 | 6.35 | 6.35 | 1.09 | 3.97 | 1492 | 0.96 | 0.973 |
| DS_F | 126 | i4 | 6.32 | 1.1 | 6.35 | 6.4 | 1.07 | 4.76 | 1257 | 0.461 | 0.576 |
| DS_F | 126 | i5 | 6.33 | 1.09 | 6.35 | 6.4 | 1.11 | 3.97 | 1110 | 0.69 | 0.809 |
| FLU_A | 124 | CC | 31.97 | 9.36 | 8.06 | 29.71 | 10.45 | 15.32 | 2469.5 | 0.002 | 0.013 |
| FLU_A | 126 | i1 | 31.87 | 9.33 | 8.73 | 29.82 | 10.58 | 15.08 | 2603.5 | 0.003 | 0.01 |
| FLU_A | 126 | i2 | 32.12 | 9.48 | 7.94 | 29.91 | 10.55 | 15.08 | 2569.5 | 0.003 | 0.01 |
| FLU_A | 126 | i3 | 31.91 | 9.3 | 7.94 | 29.84 | 10.57 | 15.08 | 2609.5 | 0.004 | 0.012 |
| FLU_A | 126 | i4 | 32.01 | 9.31 | 7.94 | 29.84 | 10.57 | 15.08 | 2596 | 0.003 | 0.01 |
| FLU_A | 126 | i5 | 31.96 | 9.29 | 7.94 | 29.92 | 10.56 | 15.08 | 2659 | 0.005 | 0.013 |
| FLU_P | 124 | CC | 22.19 | 8.11 | 8.06 | 20.69 | 8.42 | 16.13 | 2421.5 | 0.06 | 0.141 |
| FLU_P | 126 | i1 | 22.27 | 8.09 | 7.94 | 20.75 | 8.47 | 15.87 | 2496.5 | 0.052 | 0.103 |
| FLU_P | 126 | i2 | 22.28 | 8.1 | 7.94 | 20.78 | 8.45 | 15.87 | 2506.5 | 0.056 | 0.108 |
| FLU_P | 126 | i3 | 22.39 | 8.33 | 7.94 | 20.74 | 8.47 | 15.87 | 2428.5 | 0.033 | 0.069 |
| FLU_P | 126 | i4 | 22.13 | 8.09 | 8.73 | 20.69 | 8.53 | 16.67 | 2533.5 | 0.067 | 0.126 |
| FLU_P | 126 | i5 | 22.19 | 8.05 | 7.94 | 20.78 | 8.45 | 15.87 | 2549 | 0.074 | 0.132 |
| PPTT | 115 | CC | 48.9 | 2.37 | 18.26 | 49.27 | 2.32 | 13.91 | 1141 | 0.013 | 0.039 |
| PPTT | 126 | i1 | 48.78 | 2.47 | 19.84 | 49.29 | 2.33 | 14.29 | 1272.5 | 0.004 | 0.012 |
| PPTT | 126 | i2 | 48.83 | 2.46 | 19.84 | 49.21 | 2.45 | 13.49 | 1482 | 0.014 | 0.033 |
| PPTT | 126 | i3 | 48.83 | 2.4 | 19.84 | 49.32 | 2.32 | 14.29 | 1361 | 0.002 | 0.008 |
| PPTT | 126 | i4 | 48.83 | 2.39 | 19.05 | 49.33 | 2.27 | 13.49 | 1330 | 0.003 | 0.01 |
| PPTT | 126 | i5 | 48.87 | 2.42 | 19.05 | 49.28 | 2.33 | 14.29 | 1357.5 | 0.011 | 0.027 |
| RLRI_E | 83 | CC | 15.78 | 0.5 | 3.61 | 15.2 | 1.74 | 13.25 | 84 | 0 | 0.001 |
| RLRI_E | 126 | i1 | 15.71 | 0.61 | 6.35 | 15.18 | 1.61 | 15.08 | 468.5 | 0 | 0 |
| RLRI_E | 126 | i2 | 15.71 | 0.61 | 6.35 | 15.27 | 1.5 | 15.08 | 433.5 | 0 | 0 |
| RLRI_E | 126 | i3 | 15.7 | 0.61 | 6.35 | 15.25 | 1.51 | 15.08 | 370.5 | 0 | 0 |
| RLRI_E | 126 | i4 | 15.75 | 0.58 | 5.56 | 15.25 | 1.51 | 13.5 | 288 | 0 | 0 |
| RLRI_E | 126 | i5 | 15.71 | 0.59 | 5.56 | 15.28 | 1.51 | 14.29 | 367.5 | 0 | 0 |
| RLRI_FR | 70 | CC | 33.01 | 6.22 | 5.71 | 32.27 | 6.74 | 14.29 | 982.5 | 0.327 | 0.469 |
| RLRI_FR | 126 | i1 | 31.74 | 6.36 | 7.14 | 31.23 | 7.73 | 21.43 | 3382 | 0.618 | 0.748 |
| RLRI_FR | 126 | i2 | 31.98 | 6.58 | 9.52 | 30.65 | 7.45 | 22.22 | 2862.5 | 0.023 | 0.049 |
| RLRI_FR | 126 | i3 | 31.87 | 6.39 | 7.94 | 31.89 | 7.26 | 26.19 | 3451 | 0.873 | 0.935 |
| RLRI_FR | 126 | i4 | 32.03 | 6.32 | 7.94 | 31.52 | 7.77 | 26.98 | 3181.5 | 0.24 | 0.34 |
| RLRI_FR | 126 | i5 | 31.76 | 6.86 | 10.32 | 31.83 | 6.92 | 24.6 | 3427 | 0.822 | 0.907 |
| RLRI_TR | 70 | CC | 46.31 | 3.17 | 4.29 | 45.23 | 4.05 | 4.29 | 373 | 0.01 | 0.033 |
| RLRI_TR | 126 | i1 | 45.77 | 3.92 | 3.97 | 44.68 | 4.31 | 11.9 | 1526.5 | 0.002 | 0.008 |
| RLRI_TR | 126 | i2 | 45.72 | 3.69 | 3.97 | 44.89 | 4.02 | 9.52 | 1901 | 0.015 | 0.033 |
| RLRI_TR | 126 | i3 | 45.69 | 4.31 | 3.97 | 44.85 | 4.74 | 9.52 | 1703.5 | 0.015 | 0.033 |
| RLRI_TR | 126 | i4 | 45.02 | 5.01 | 3.18 | 45.02 | 3.97 | 10.32 | 2317 | 0.299 | 0.408 |
| RLRI_TR | 126 | i5 | 45.58 | 4.38 | 3.18 | 44.4 | 5.13 | 8.73 | 1681.5 | 0.008 | 0.02 |
| STROOP_D | 107 | CC | 60.21 | 10.58 | 9.35 | 66.62 | 21.32 | 24.3 | 1417 | 0 | 0.001 |
| STROOP_D | 126 | i1 | 60.57 | 10.74 | 11.11 | 65.43 | 20.4 | 23.81 | 2420.5 | 0.002 | 0.008 |
| STROOP_D | 126 | i2 | 60.07 | 10.17 | 8.73 | 65.41 | 20.29 | 22.22 | 2319 | 0.001 | 0.005 |
| STROOP_D | 126 | i3 | 60.34 | 10.1 | 10.32 | 65.63 | 20.94 | 23.02 | 2499 | 0.003 | 0.01 |
| STROOP_D | 126 | i4 | 60.43 | 10.14 | 10.32 | 66.37 | 20.43 | 25.4 | 2133.5 | 0 | 0 |
| STROOP_D | 126 | i5 | 59.97 | 10.76 | 10.32 | 65.75 | 20.25 | 21.43 | 2237 | 0 | 0 |
| STROOP_ID | 103 | CC | 41 | 16.16 | 6.8 | 43.63 | 27.72 | 10.68 | 2386 | 0.521 | 0.619 |
| STROOP_ID | 126 | i1 | 42.71 | 23.92 | 7.14 | 43.74 | 29.26 | 10.32 | 3542 | 0.406 | 0.516 |
| STROOP_ID | 126 | i2 | 43.22 | 23.97 | 8.73 | 42.27 | 28.41 | 9.52 | 3217 | 0.101 | 0.176 |
| STROOP_ID | 126 | i3 | 44.33 | 27.47 | 8.73 | 43.94 | 30.91 | 10.32 | 3224 | 0.137 | 0.219 |
| STROOP_ID | 126 | i4 | 45.21 | 28.13 | 9.52 | 43.02 | 28.5 | 9.52 | 3174 | 0.107 | 0.182 |
| STROOP_ID | 126 | i5 | 43.5 | 24.24 | 7.14 | 42.62 | 26.37 | 9.52 | 3353.5 | 0.193 | 0.302 |
| STROOP_R | 106 | CC | 43.54 | 6.38 | 10.38 | 47.82 | 14.04 | 25.47 | 979.5 | 0 | 0 |
| STROOP_R | 126 | i1 | 43.23 | 6.18 | 9.52 | 47.54 | 13.14 | 24.6 | 1328.5 | 0 | 0 |
| STROOP_R | 126 | i2 | 43.06 | 6.18 | 9.52 | 47.56 | 13.26 | 26.98 | 1357 | 0 | 0 |
| STROOP_R | 126 | i3 | 43.78 | 6.63 | 12.7 | 47.16 | 13.6 | 24.6 | 2009 | 0 | 0 |
| STROOP_R | 126 | i4 | 43.72 | 6.64 | 11.91 | 47.07 | 13.21 | 24.6 | 1902 | 0 | 0 |
| STROOP_R | 126 | i5 | 43.61 | 6.7 | 11.91 | 47.29 | 13.4 | 25.4 | 1811.5 | 0 | 0 |
| TMT_A | 112 | CC | 27.92 | 9.27 | 0 | 29.82 | 16 | 1.79 | 2436.5 | 0.525 | 0.619 |
| TMT_A | 126 | i1 | 27.67 | 9.07 | 0 | 29.44 | 15.23 | 1.59 | 3012 | 0.367 | 0.488 |
| TMT_A | 126 | i2 | 27.82 | 9.16 | 0 | 29.45 | 15.35 | 2.38 | 3254.5 | 0.703 | 0.811 |
| TMT_A | 126 | i3 | 27.76 | 8.96 | 0 | 29.48 | 15.23 | 1.59 | 3162 | 0.524 | 0.644 |
| TMT_A | 126 | i4 | 27.6 | 8.99 | 0 | 29.73 | 15.29 | 2.38 | 2960 | 0.232 | 0.34 |
| TMT_A | 126 | i5 | 27.42 | 8.98 | 0 | 29.33 | 15.23 | 1.59 | 3015 | 0.371 | 0.488 |
| TMT_BA | 108 | CC | 40.49 | 20.24 | 6.48 | 45.37 | 31.58 | 15.74 | 2496.5 | 0.171 | 0.314 |
| TMT_BA | 126 | i1 | 39.87 | 19.48 | 5.56 | 44.6 | 30 | 15.08 | 3368.5 | 0.124 | 0.207 |
| TMT_BA | 126 | i2 | 41.44 | 20.47 | 7.94 | 47.75 | 35.23 | 17.46 | 3380 | 0.131 | 0.214 |
| TMT_BA | 126 | i3 | 40.28 | 19.65 | 6.35 | 46.88 | 34.92 | 15.87 | 3263 | 0.072 | 0.132 |
| TMT_BA | 126 | i4 | 39.65 | 19.44 | 5.56 | 46.59 | 34.33 | 15.87 | 3130.5 | 0.034 | 0.069 |
| TMT_BA | 126 | i5 | 40.92 | 20.43 | 7.94 | 45.28 | 30.61 | 14.29 | 3509 | 0.231 | 0.34 |

CC, Complete Cases, I_ID, = Imputation Number; FDR = False Discovery Rate.

**Supplementary Table 8:** Statistical comparisons between the pre- and postoperative assessments across imputations for patients with a right-sided tumor

| **Task** | **N** | **I_ID** | **pre-surgery** | | | **post-surgery** | | | **Wilcoxon** | ***p*** | ***p*FDR** |
| --- | --- | --- | --- | --- | --- | --- | --- | --- | --- | --- | --- |
|  |  |  | **mean** | **SD** | **Rate** | **mean** | **SD** | **Rate** |  |  |  |
| BELLS | 116 | CC | 2.09 | 2.43 | 11.21 | 1.81 | 1.9 | 11.21 | 1366 | 0.217 | 0.358 |
| BELLS | 126 | i1 | 2.1 | 2.47 | 11.91 | 1.83 | 1.86 | 11.12 | 1773 | 0.342 | 0.486 |
| BELLS | 126 | i2 | 2.08 | 2.41 | 11.12 | 1.83 | 1.94 | 11.12 | 1639 | 0.238 | 0.405 |
| BELLS | 126 | i3 | 2.07 | 2.41 | 11.12 | 1.87 | 1.89 | 11.91 | 1848.5 | 0.418 | 0.547 |
| BELLS | 126 | i4 | 2.06 | 2.41 | 11.12 | 1.82 | 1.88 | 10.32 | 1774 | 0.343 | 0.486 |
| BELLS | 126 | i5 | 2.08 | 2.45 | 11.91 | 1.85 | 1.89 | 11.12 | 1713.5 | 0.303 | 0.486 |
| CODES | 88 | CC | 73.82 | 15.87 | 9.09 | 73.33 | 15.39 | 3.41 | 1631.5 | 0.493 | 0.619 |
| CODES | 126 | i1 | 73.18 | 14.67 | 7.15 | 72.09 | 15.71 | 3.18 | 3336.5 | 0.442 | 0.561 |
| CODES | 126 | i2 | 73.68 | 15.04 | 6.35 | 72.22 | 15.35 | 3.18 | 3184.5 | 0.19 | 0.336 |
| CODES | 126 | i3 | 72.8 | 15.35 | 7.15 | 72.56 | 15.16 | 3.18 | 3578 | 0.657 | 0.739 |
| CODES | 126 | i4 | 72.75 | 15.06 | 7.15 | 71.39 | 15.53 | 3.97 | 3104.5 | 0.217 | 0.376 |
| CODES | 126 | i5 | 73.79 | 15.64 | 7.94 | 72.56 | 15.66 | 3.18 | 3304.5 | 0.318 | 0.486 |
| DS_B | 92 | CC | 5.1 | 1.19 | 4.35 | 5.28 | 1.19 | 4.35 | 548 | 0.073 | 0.151 |
| DS_B | 126 | i1 | 5.26 | 1.3 | 4.76 | 5.43 | 1.24 | 3.97 | 1468.5 | 0.14 | 0.277 |
| DS_B | 126 | i2 | 5.13 | 1.23 | 4.76 | 5.37 | 1.22 | 3.97 | 1166 | 0.014 | 0.051 |
| DS_B | 126 | i3 | 5.12 | 1.21 | 3.97 | 5.27 | 1.21 | 5.56 | 1415.5 | 0.163 | 0.308 |
| DS_B | 126 | i4 | 5.15 | 1.27 | 5.56 | 5.33 | 1.18 | 4.76 | 1273.5 | 0.084 | 0.179 |
| DS_B | 126 | i5 | 5.13 | 1.18 | 5.56 | 5.37 | 1.21 | 3.97 | 1126 | 0.029 | 0.075 |
| DS_F | 92 | CC | 6.34 | 1.08 | 3.26 | 6.54 | 1.18 | 4.35 | 646 | 0.085 | 0.164 |
| DS_F | 126 | i1 | 6.31 | 1.1 | 3.18 | 6.7 | 1.19 | 3.18 | 1048 | 0.002 | 0.014 |
| DS_F | 126 | i2 | 6.33 | 1.07 | 2.38 | 6.6 | 1.17 | 3.97 | 1373.5 | 0.025 | 0.069 |
| DS_F | 126 | i3 | 6.31 | 1.08 | 2.38 | 6.57 | 1.16 | 3.18 | 1162.5 | 0.022 | 0.067 |
| DS_F | 126 | i4 | 6.34 | 1.08 | 2.38 | 6.61 | 1.17 | 4.76 | 1199.5 | 0.015 | 0.051 |
| DS_F | 126 | i5 | 6.35 | 1.04 | 2.38 | 6.58 | 1.18 | 3.97 | 1183 | 0.026 | 0.069 |
| FIG_C | 90 | CC | 35.68 | 0.77 | 0 | 35.47 | 1.21 | 1.11 | 168.5 | 0.067 | 0.148 |
| FIG_C | 126 | i1 | 35.61 | 0.86 | 0 | 35.42 | 1.36 | 1.59 | 430.5 | 0.15 | 0.29 |
| FIG_C | 126 | i2 | 35.62 | 0.88 | 0 | 35.47 | 1.15 | 0.8 | 425 | 0.134 | 0.271 |
| FIG_C | 126 | i3 | 35.58 | 0.97 | 0 | 35.46 | 1.15 | 0.8 | 550.5 | 0.283 | 0.463 |
| FIG_C | 126 | i4 | 35.61 | 0.89 | 0 | 35.49 | 1.14 | 0.8 | 497 | 0.341 | 0.486 |
| FIG_C | 126 | i5 | 35.61 | 0.86 | 0 | 35.41 | 1.37 | 1.59 | 444 | 0.134 | 0.271 |
| FIG_R | 70 | CC | 23.03 | 6.46 | 15.71 | 24.99 | 5.44 | 7.14 | 783.5 | 0.007 | 0.029 |
| FIG_R | 126 | i1 | 22.8 | 7.05 | 22.8 | 24.82 | 5.4 | 9.53 | 2672 | 0.003 | 0.018 |
| FIG_R | 126 | i2 | 23.04 | 7.03 | 23.05 | 25.02 | 5.58 | 8.74 | 2693 | 0.005 | 0.025 |
| FIG_R | 126 | i3 | 22.89 | 6.67 | 22.89 | 24.78 | 5.57 | 7.94 | 2846 | 0.005 | 0.025 |
| FIG_R | 126 | i4 | 23.71 | 6.46 | 23.71 | 23.95 | 5.77 | 11.91 | 3477 | 0.581 | 0.667 |
| FIG_R | 126 | i5 | 22.73 | 7 | 22.74 | 23.83 | 6.07 | 14.29 | 3214 | 0.075 | 0.163 |
| FLU_A | 121 | CC | 32.83 | 9.82 | 6.61 | 31.87 | 9.75 | 12.4 | 2721.5 | 0.323 | 0.469 |
| FLU_A | 126 | i1 | 32.8 | 9.8 | 6.35 | 31.92 | 9.8 | 12.7 | 3000.5 | 0.35 | 0.488 |
| FLU_A | 126 | i2 | 32.81 | 9.78 | 6.35 | 31.93 | 9.67 | 11.91 | 3043.5 | 0.415 | 0.547 |
| FLU_A | 126 | i3 | 32.74 | 9.68 | 6.35 | 31.91 | 9.63 | 11.91 | 3031.5 | 0.396 | 0.536 |
| FLU_A | 126 | i4 | 33.22 | 10.03 | 6.35 | 31.96 | 9.63 | 11.91 | 2861.5 | 0.186 | 0.336 |
| FLU_A | 126 | i5 | 32.86 | 9.76 | 6.35 | 31.89 | 9.68 | 11.91 | 2921 | 0.313 | 0.486 |
| FLU_P | 123 | CC | 22.56 | 7.26 | 9.76 | 23.28 | 8.27 | 8.94 | 3189.5 | 0.388 | 0.534 |
| FLU_P | 126 | i1 | 22.52 | 7.22 | 9.52 | 23.13 | 8.26 | 9.52 | 3399.5 | 0.451 | 0.561 |
| FLU_P | 126 | i2 | 22.45 | 7.25 | 10.32 | 23.07 | 8.29 | 9.52 | 3365.5 | 0.488 | 0.576 |
| FLU_P | 126 | i3 | 22.56 | 7.22 | 9.52 | 23.32 | 8.38 | 9.52 | 3317 | 0.333 | 0.486 |
| FLU_P | 126 | i4 | 22.46 | 7.25 | 10.32 | 23.09 | 8.3 | 10.32 | 3355 | 0.471 | 0.567 |
| FLU_P | 126 | i5 | 22.63 | 7.27 | 9.52 | 23.17 | 8.25 | 9.52 | 3338 | 0.444 | 0.561 |
| LB | 104 | CC | -1.08 | 2.48 | 6.73 | -0.83 | 2.83 | 13.46 | 2068 | 0.431 | 0.569 |
| LB | 126 | i1 | -1.24 | 2.44 | 6.35 | -1.04 | 2.73 | 11.91 | 3133 | 0.474 | 0.567 |
| LB | 126 | i2 | -1.08 | 2.37 | 6.35 | -0.89 | 2.76 | 12.7 | 3177 | 0.455 | 0.561 |
| LB | 126 | i3 | -1.19 | 2.37 | 6.35 | -1.02 | 2.74 | 12.7 | 3211.5 | 0.514 | 0.598 |
| LB | 126 | i4 | -1.06 | 2.36 | 6.35 | -0.99 | 2.72 | 11.91 | 3333.5 | 0.997 | 0.997 |
| LB | 126 | i5 | -1.09 | 2.36 | 6.35 | -1.02 | 2.67 | 11.91 | 3333.5 | 0.87 | 0.924 |
| RLRI_E | 69 | CC | 15.64 | 0.66 | 7.25 | 15.86 | 0.46 | 1.45 | 51 | 0.008 | 0.029 |
| RLRI_E | 126 | i1 | 15.69 | 0.65 | 7.15 | 15.83 | 0.43 | 0.8 | 226.5 | 0.025 | 0.069 |
| RLRI_E | 126 | i2 | 15.68 | 0.67 | 7.94 | 15.87 | 0.4 | 0.8 | 157.5 | 0.002 | 0.014 |
| RLRI_E | 126 | i3 | 15.73 | 0.61 | 5.56 | 15.86 | 0.47 | 1.59 | 180 | 0.031 | 0.078 |
| RLRI_E | 126 | i4 | 15.66 | 0.71 | 7.15 | 15.82 | 0.5 | 1.59 | 263 | 0.02 | 0.063 |
| RLRI_E | 126 | i5 | 15.71 | 0.65 | 7.15 | 15.86 | 0.47 | 1.59 | 152 | 0.015 | 0.051 |
| RLRI_FR | 69 | CC | 32.68 | 5.39 | 5.8 | 34.57 | 5.79 | 5.8 | 451 | 0 | 0.002 |
| RLRI_FR | 126 | i1 | 32.48 | 5.54 | 5.56 | 34.9 | 5.7 | 5.56 | 1543.5 | 0 | 0 |
| RLRI_FR | 126 | i2 | 32.41 | 5.66 | 5.56 | 34.81 | 5.54 | 4.76 | 1749.5 | 0 | 0 |
| RLRI_FR | 126 | i3 | 32.68 | 5.67 | 5.56 | 34.92 | 5.69 | 3.97 | 1977 | 0 | 0 |
| RLRI_FR | 126 | i4 | 32.52 | 6.07 | 5.56 | 34.96 | 5.67 | 3.97 | 1698.5 | 0 | 0 |
| RLRI_FR | 126 | i5 | 32.51 | 5.65 | 7.14 | 34.57 | 5.85 | 3.97 | 1804.5 | 0 | 0 |
| RLRI_TR | 69 | CC | 46.43 | 2.42 | 7.25 | 46.48 | 2.4 | 2.9 | 402.5 | 0.918 | 0.947 |
| RLRI_TR | 126 | i1 | 46.28 | 2.54 | 4.77 | 46.4 | 2.68 | 2.39 | 1477.5 | 0.751 | 0.808 |
| RLRI_TR | 126 | i2 | 46.49 | 2.26 | 4.77 | 46.62 | 2.29 | 2.39 | 1379.5 | 0.661 | 0.739 |
| RLRI_TR | 126 | i3 | 46.44 | 2.36 | 4.77 | 46.46 | 2.54 | 2.39 | 1646 | 0.945 | 0.98 |
| RLRI_TR | 126 | i4 | 46.23 | 2.35 | 5.56 | 46.25 | 3.11 | 2.39 | 1520 | 0.397 | 0.536 |
| RLRI_TR | 126 | i5 | 46.44 | 2.24 | 4.77 | 46.63 | 2.42 | 2.39 | 1314 | 0.334 | 0.486 |
| STROOP_D | 113 | CC | 58.33 | 11.54 | 7.08 | 58.53 | 12.48 | 10.62 | 2717.5 | 0.709 | 0.78 |
| STROOP_D | 126 | i1 | 58.98 | 11.31 | 7.14 | 59.75 | 14 | 12.7 | 3448 | 0.746 | 0.808 |
| STROOP_D | 126 | i2 | 58.29 | 11.14 | 6.35 | 59.02 | 12.63 | 11.91 | 3553 | 0.964 | 0.987 |
| STROOP_D | 126 | i3 | 58.66 | 11.14 | 6.35 | 58.9 | 12.67 | 11.11 | 3449 | 0.748 | 0.808 |
| STROOP_D | 126 | i4 | 58.12 | 11.29 | 6.35 | 58.83 | 12.76 | 11.91 | 3483 | 0.941 | 0.98 |
| STROOP_D | 126 | i5 | 58.19 | 11.24 | 6.35 | 59.21 | 12.85 | 11.91 | 3560.5 | 0.98 | 0.992 |
| STROOP_ID | 115 | CC | 43.47 | 16.09 | 3.48 | 40.07 | 17.68 | 6.96 | 1879.5 | 0.001 | 0.007 |
| STROOP_ID | 126 | i1 | 43.62 | 16.95 | 4.76 | 39.63 | 17.15 | 6.35 | 2347 | 0.001 | 0.008 |
| STROOP_ID | 126 | i2 | 44.1 | 16.97 | 3.97 | 40.15 | 17.17 | 7.14 | 2266.5 | 0.001 | 0.008 |
| STROOP_ID | 126 | i3 | 43.87 | 17.06 | 3.97 | 39.52 | 17.18 | 6.35 | 2239.5 | 0 | 0 |
| STROOP_ID | 126 | i4 | 43.45 | 16.62 | 3.97 | 39.64 | 17.12 | 6.35 | 2285.5 | 0.001 | 0.008 |
| STROOP_ID | 126 | i5 | 43.52 | 16.69 | 3.97 | 39.75 | 17.08 | 6.35 | 2342 | 0.001 | 0.008 |
| STROOP_R | 112 | CC | 43.64 | 10.33 | 15.18 | 44.65 | 9.09 | 19.64 | 2092.5 | 0.027 | 0.069 |
| STROOP_R | 126 | i1 | 43.18 | 9.96 | 13.49 | 44.49 | 8.94 | 19.05 | 2565.5 | 0.008 | 0.034 |
| STROOP_R | 126 | i2 | 43.28 | 9.88 | 13.49 | 44.94 | 8.99 | 19.84 | 2460.5 | 0.003 | 0.018 |
| STROOP_R | 126 | i3 | 43.39 | 9.94 | 14.29 | 44.51 | 9.07 | 19.05 | 2726 | 0.025 | 0.069 |
| STROOP_R | 126 | i4 | 43.56 | 10 | 14.29 | 45.03 | 9.49 | 21.43 | 2655.5 | 0.015 | 0.051 |
| STROOP_R | 126 | i5 | 43.49 | 10.23 | 15.08 | 44.5 | 8.94 | 19.05 | 2798 | 0.04 | 0.097 |
| TMT_A | 116 | CC | 25.91 | 9.25 | 1.72 | 26.9 | 10.34 | 1.72 | 2513.5 | 0.243 | 0.381 |
| TMT_A | 126 | i1 | 25.37 | 9.11 | 1.59 | 26.73 | 10.09 | 1.59 | 2782 | 0.068 | 0.152 |
| TMT_A | 126 | i2 | 25.75 | 9.15 | 1.59 | 26.82 | 10.23 | 1.59 | 2966.5 | 0.187 | 0.336 |
| TMT_A | 126 | i3 | 26 | 9.57 | 1.59 | 26.77 | 10.16 | 1.59 | 3044 | 0.267 | 0.445 |
| TMT_A | 126 | i4 | 25.46 | 9.04 | 1.59 | 27.18 | 10.36 | 1.59 | 2743.5 | 0.054 | 0.124 |
| TMT_A | 126 | i5 | 25.31 | 9.17 | 1.59 | 27.3 | 10.46 | 1.59 | 2653.5 | 0.041 | 0.097 |
| TMT_BA | 122 | CC | 35.05 | 15.97 | 3.28 | 33.11 | 22.22 | 2.46 | 2554 | 0.015 | 0.04 |
| TMT_BA | 126 | i1 | 35.43 | 16.74 | 4.76 | 33.69 | 22.99 | 3.18 | 2782.5 | 0.019 | 0.062 |
| TMT_BA | 126 | i2 | 35.45 | 16.41 | 3.97 | 32.6 | 22.18 | 2.38 | 2598.5 | 0.005 | 0.025 |
| TMT_BA | 126 | i3 | 35.32 | 16.37 | 3.97 | 33.04 | 21.98 | 2.38 | 2719.5 | 0.012 | 0.049 |
| TMT_BA | 126 | i4 | 35.78 | 16.79 | 4.76 | 32.94 | 21.9 | 2.38 | 2636.5 | 0.006 | 0.028 |
| TMT_BA | 126 | i5 | 35.45 | 16.45 | 3.97 | 32.71 | 22.03 | 2.38 | 2646 | 0.007 | 0.031 |

CC, Complete Cases, I_ID, = Imputation Number; FDR = False Discovery Rate.

**Supplementary Table 9:** Retained cortical parcels and white matter tracts

| **Left** | | **Right** | |
| --- | --- | --- | --- |
| **SD** | **CD** | **SD** | **CD** |
| AC | SVA_A_Ins_2 | AC | SMB_Aud_2 |
| AF | SVA_A_Ins_3 | AF | SVA_A_Ins_2 |
| C_FP | SVA_B_PFCmp_2 | C_FP | SVA_A_FrMed_2 |
| C_PH | LimbB_OFC_2 | C_FPH | SVA_B_PFCmp_2 |
| CC_A | LimbA_TP_2 | C_PH | LimbB_OFC_2 |
| CC_MidA | ContA_PFCl_2 | C_PHP | LimbA_TP_2 |
| CC_P | DMN_B_Temp_2 | CC_A | ContA_PFCl_2 |
| CPT_F | DMN_B_PFCd_2 | CC_MidA | ContB_Temp_2 |
| CS_A | DMN_B_PFCv_2 | CC_P | DMN_A_PFCd_2 |
| CS_S | DMN_B_PFCv_3 | CPT_F | DMN_B_PFCd_2 |
| EMC |  | CS_A | DMN_B_PFCv_2 |
| F |  | CS_S | DMN_B_PFCv_3 |
| FAT |  | EMC | TP_2 |
| IFOF |  | F |  |
| ILF |  | FAT |  |
| MdLF |  | IFOF |  |
| RST |  | ILF |  |
| SLF1 |  | RST |  |
| SLF2 |  | SLF1 |  |
| TR_A |  | SLF2 |  |
| TR_S |  | SLF3 |  |
| UF |  | TR_A |  |
|  |  | TR_S |  |
|  |  | UF |  |

SD = Structural Disconnection nodes; CD = Cortical Damage nodes. See the Methods section for details about the selection procedure. Refer to Supplementary Table 1 for complete definition of acronyms.

**Supplementary Table 10**: Descriptive statistics of graph connectivity

| **Hemi** | **Type** | **Layer** | **median** | **mean** | **std** | **min** | **max** |
| --- | --- | --- | --- | --- | --- | --- | --- |
| Left | Single Layer | NT | 0.0728 | 0.0826 | 0.1168 | -0.108 | 0.5476 |
| Left | Single Layer | SD | 0.193 | 0.1414 | 0.4513 | -0.709 | 0.988 |
| Left | Single Layer | CD | 0.219 | 0.2396 | 0.4265 | -0.48 | 0.942 |
| Right | Single Layer | NT | 0.0316 | 0.0409 | 0.0942 | -0.231 | 0.4682 |
| Right | Single Layer | SD | 0.2135 | 0.1659 | 0.4105 | -0.637 | 0.979 |
| Right | Single Layer | CD | 0.166 | 0.1638 | 0.5085 | -0.579 | 0.928 |
| Left | Layer Pairs | NT-SD | 0.0461 | 0.0403 | 0.1308 | -0.395 | 0.402 |
| Left | Layer Pairs | NT-CD | 0.0281 | 0.0173 | 0.118 | -0.265 | 0.281 |
| Left | Layer Pairs | SD-CD | 0.1975 | 0.1916 | 0.4053 | -0.62 | 0.866 |
| Right | Layer Pairs | NT-SD | 0.0193 | 0.026 | 0.0868 | -0.1654 | 0.3098 |
| Right | Layer Pairs | NT-CD | 0.0072 | 0.0114 | 0.0857 | -0.192 | 0.2368 |
| Right | Layer Pairs | SD-CD | 0.2225 | 0.1802 | 0.4219 | -0.728 | 0.899 |
| Left | All layers | NT-SD-CD | 0.0692 | 0.1019 | 0.313 | -0.709 | 0.988 |
| Right | All layers | NT-SD-CD | 0.0402 | 0.0933 | 0.3083 | -0.728 | 0.979 |

**Supplementary Table 11**: Within and between hemisphere connectivity comparisons

| **Comparison type** | **Hemi** | **Comparison** | **Mean_diff** | ***p*** | ***p*FDR** |
| --- | --- | --- | --- | --- | --- |
| Within hemisphere | Left | NT-SD vs NT-CD | 0.023 | 0.066 | 0.066 |
| Within hemisphere | Right | NT-SD vs NT-CD | 0.0146 | 0.0478 | 0.066 |
| Between Hemisphere | Left vs Right | NT | 0.0417 | 0.003 | 0.009 |
| Between Hemisphere | Left vs Right | NT-SD | 0.0142 | 0.0854 | 0.1281 |
| Between Hemisphere | Left vs Right | NT-CD | 0.0059 | 0.5547 | 0.5547 |

Note: Statistical comparisons were performed with permutation testing (10000 permutations).

**Supplementary Table 12**: Network density for each single layer

| **Hemisphere** | **Layer** | **Nodes** | **Possible Connections** | **Actual Connections** | **Density** |
| --- | --- | --- | --- | --- | --- |
| Left | NT | 15 | 105 | 23 | 0.2190 |
| Left | SD | 22 | 231 | 191 | 0.8268 |
| Left | CD | 10 | 45 | 31 | 0.6889 |
| Right | NT | 17 | 136 | 8 | 0.0588 |
| Right | SD | 24 | 276 | 223 | 0.8080 |
| Right | CD | 13 | 78 | 69 | 0.8846 |

Note: the formula to compute all possible connections for a single layer is: *n*(*n*-1)/2 where *n* is the number of nodes. Network density = actual connections above threshold (|R| = 0.175)/total possible connections.

**Supplementary Table 13**: Network density for each pair of layers and full network

| **Hemisphere** | **Layer Pair** | **Nodes L1** | **Nodes L2** | **Possible Con** | **Actual Con** | **Density** |
| --- | --- | --- | --- | --- | --- | --- |
| Left | NT-SD | 15 | 22 | 330 | 57 | 0.1727 |
| Left | NT-CD | 15 | 10 | 150 | 21 | 0.1400 |
| Left | SD-CD | 22 | 10 | 220 | 171 | 0.7773 |
| Left | Full Network | 48 | 48 | 1128 | 494 | 0.4379 |
| Right | NT-SD | 17 | 24 | 408 | 18 | 0.0441 |
| Right | NT-CD | 17 | 13 | 221 | 10 | 0.0452 |
| Right | SD-CD | 24 | 13 | 312 | 243 | 0.7788 |
| Right | Full Network | 55 | 55 | 1485 | 571 | 0.384 |

Note: the formula to compute all possible connections for two different layers is *n*1**n*2 where *n*1 is the number of nodes for layer 1 (L1) and *n*2 the number of nodes for layer 2 (L2). For the full network, the formula is (*n*_nodes*(*n*_nodes-1))/2. Network density = actual connections above threshold (|R| = 0.175)/total possible connections.

**Supplementary Table 14**: Node-controlled density analysis

| **Thres** | **Hem** | **Type** | **Name** | **Mean_Density** | **SD_Density** | ***p*** | ***p*FDR** |
| --- | --- | --- | --- | --- | --- | --- | --- |
| 0,147 | Left | Layer | CD | 0,778 | 0,000 |  |  |
| 0,147 | Left | Layer | NT | 0,253 | 0,067 |  |  |
| 0,147 | Left | Layer | SD | 0,861 | 0,063 |  |  |
| 0,147 | Left | Pair | NT-CD | 0,245 | 0,039 |  |  |
| 0,147 | Left | Pair | NT-SD | 0,288 | 0,024 |  |  |
| 0,147 | Left | Pair | SD-CD | 0,804 | 0,028 |  |  |
| 0,147 | Left | Full | Full Network | 0,504 | 0,024 |  |  |
| 0,147 | Left | LayerComp | NT_vs_CD | -0,524 |  | 0,000 | 0,000 |
| 0,147 | Left | LayerComp | NT_vs_SD | -0,608 |  | 0,000 | 0,000 |
| 0,147 | Left | LayerComp | SD_vs_CD | 0,083 |  | 0,179 | 0,210 |
| 0,147 | Left | PairComp | NT-CD_vs_SD-CD | -0,559 |  | 0,000 | 0,000 |
| 0,147 | Left | PairComp | NT-SD_vs_NT-CD | 0,043 |  | 0,381 | 0,431 |
| 0,147 | Left | PairComp | NT-SD_vs_SD-CD | -0,516 |  | 0,000 | 0,000 |
| 0,147 | Left_vs_Right | PairBetweenHemi | NT-CD | 0,160 |  | 0,000 | 0,001 |
| 0,147 | Left_vs_Right | PairBetweenHemi | NT-SD | 0,190 |  | 0,000 | 0,001 |
| 0,147 | Left_vs_Right | PairBetweenHemi | SD-CD | -0,006 |  | 0,913 | 0,969 |
| 0,147 | Left_vs_Right | LayerBetweenHemi | CD | -0,145 |  | 0,027 | 0,052 |
| 0,147 | Left_vs_Right | LayerBetweenHemi | NT | 0,115 |  | 0,031 | 0,054 |
| 0,147 | Left_vs_Right | LayerBetweenHemi | SD | 0,020 |  | 0,530 | 0,640 |
| 0,147 | Left_vs_Right | FullBetweenHemi | Full Network | 0,077 |  | 0,000 | 0,001 |
| 0,147 | Right | Layer | CD | 0,923 | 0,000 |  |  |
| 0,147 | Right | Layer | NT | 0,139 | 0,036 |  |  |
| 0,147 | Right | Layer | SD | 0,841 | 0,038 |  |  |
| 0,147 | Right | Pair | NT-CD | 0,085 | 0,016 |  |  |
| 0,147 | Right | Pair | NT-SD | 0,098 | 0,010 |  |  |
| 0,147 | Right | Pair | SD-CD | 0,810 | 0,025 |  |  |
| 0,147 | Right | Full | Full Network | 0,428 | 0,010 |  |  |
| 0,147 | Right | LayerComp | NT_vs_CD | -0,784 |  | 0,000 | 0,000 |
| 0,147 | Right | LayerComp | NT_vs_SD | -0,702 |  | 0,000 | 0,000 |
| 0,147 | Right | LayerComp | SD_vs_CD | -0,082 |  | 0,063 | 0,082 |
| 0,147 | Right | PairComp | NT-CD_vs_SD-CD | -0,724 |  | 0,000 | 0,000 |
| 0,147 | Right | PairComp | NT-SD_vs_NT-CD | 0,013 |  | 0,666 | 0,714 |
| 0,147 | Right | PairComp | NT-SD_vs_SD-CD | -0,711 |  | 0,000 | 0,000 |
| 0,175 | Left | Layer | CD | 0,689 | 0,000 |  |  |
| 0,175 | Left | Layer | NT | 0,216 | 0,063 |  |  |
| 0,175 | Left | Layer | SD | 0,826 | 0,071 |  |  |
| 0,175 | Left | Pair | NT-CD | 0,139 | 0,032 |  |  |
| 0,175 | Left | Pair | NT-SD | 0,172 | 0,018 |  |  |
| 0,175 | Left | Pair | SD-CD | 0,777 | 0,032 |  |  |
| 0,175 | Left | Full | Full Network | 0,430 | 0,022 |  |  |
| 0,175 | Left | LayerComp | NT_vs_CD | -0,473 |  | 0,000 | 0,000 |
| 0,175 | Left | LayerComp | NT_vs_SD | -0,611 |  | 0,000 | 0,000 |
| 0,175 | Left | LayerComp | SD_vs_CD | 0,138 |  | 0,036 | 0,049 |
| 0,175 | Left | PairComp | NT-CD_vs_SD-CD | -0,638 |  | 0,000 | 0,000 |
| 0,175 | Left | PairComp | NT-SD_vs_NT-CD | 0,034 |  | 0,420 | 0,467 |
| 0,175 | Left | PairComp | NT-SD_vs_SD-CD | -0,605 |  | 0,000 | 0,000 |
| 0,175 | Left_vs_Right | PairBetweenHemi | NT-CD | 0,094 |  | 0,002 | 0,009 |
| 0,175 | Left_vs_Right | PairBetweenHemi | NT-SD | 0,128 |  | 0,000 | 0,001 |
| 0,175 | Left_vs_Right | PairBetweenHemi | SD-CD | -0,001 |  | 1,000 | 1,000 |
| 0,175 | Left_vs_Right | LayerBetweenHemi | CD | -0,196 |  | 0,009 | 0,027 |
| 0,175 | Left_vs_Right | LayerBetweenHemi | NT | 0,157 |  | 0,000 | 0,002 |
| 0,175 | Left_vs_Right | LayerBetweenHemi | SD | 0,018 |  | 0,649 | 0,733 |
| 0,175 | Left_vs_Right | FullBetweenHemi | Full Network | 0,048 |  | 0,004 | 0,014 |
| 0,175 | Right | Layer | CD | 0,885 | 0,000 |  |  |
| 0,175 | Right | Layer | NT | 0,058 | 0,019 |  |  |
| 0,175 | Right | Layer | SD | 0,809 | 0,043 |  |  |
| 0,175 | Right | Pair | NT-CD | 0,045 | 0,013 |  |  |
| 0,175 | Right | Pair | NT-SD | 0,044 | 0,006 |  |  |
| 0,175 | Right | Pair | SD-CD | 0,778 | 0,029 |  |  |
| 0,175 | Right | Full | Full Network | 0,382 | 0,009 |  |  |
| 0,175 | Right | LayerComp | NT_vs_CD | -0,826 |  | 0,000 | 0,000 |
| 0,175 | Right | LayerComp | NT_vs_SD | -0,750 |  | 0,000 | 0,000 |
| 0,175 | Right | LayerComp | SD_vs_CD | -0,076 |  | 0,129 | 0,155 |
| 0,175 | Right | PairComp | NT-CD_vs_SD-CD | -0,733 |  | 0,000 | 0,000 |
| 0,175 | Right | PairComp | NT-SD_vs_NT-CD | 0,000 |  | 1,000 | 1,000 |
| 0,175 | Right | PairComp | NT-SD_vs_SD-CD | -0,733 |  | 0,000 | 0,000 |
| 0,229 | Left | Layer | CD | 0,644 | 0,000 |  |  |
| 0,229 | Left | Layer | NT | 0,093 | 0,044 |  |  |
| 0,229 | Left | Layer | SD | 0,771 | 0,088 |  |  |
| 0,229 | Left | Pair | NT-CD | 0,053 | 0,020 |  |  |
| 0,229 | Left | Pair | NT-SD | 0,075 | 0,009 |  |  |
| 0,229 | Left | Pair | SD-CD | 0,699 | 0,033 |  |  |
| 0,229 | Left | Full | Full Network | 0,347 | 0,018 |  |  |
| 0,229 | Left | LayerComp | NT_vs_CD | -0,551 |  | 0,000 | 0,000 |
| 0,229 | Left | LayerComp | NT_vs_SD | -0,678 |  | 0,000 | 0,000 |
| 0,229 | Left | LayerComp | SD_vs_CD | 0,126 |  | 0,086 | 0,108 |
| 0,229 | Left | PairComp | NT-CD_vs_SD-CD | -0,647 |  | 0,000 | 0,000 |
| 0,229 | Left | PairComp | NT-SD_vs_NT-CD | 0,023 |  | 0,432 | 0,471 |
| 0,229 | Left | PairComp | NT-SD_vs_SD-CD | -0,624 |  | 0,000 | 0,000 |
| 0,229 | Left_vs_Right | PairBetweenHemi | NT-CD | 0,048 |  | 0,004 | 0,014 |
| 0,229 | Left_vs_Right | PairBetweenHemi | NT-SD | 0,068 |  | 0,000 | 0,001 |
| 0,229 | Left_vs_Right | PairBetweenHemi | SD-CD | -0,007 |  | 0,846 | 0,926 |
| 0,229 | Left_vs_Right | LayerBetweenHemi | CD | -0,189 |  | 0,026 | 0,052 |
| 0,229 | Left_vs_Right | LayerBetweenHemi | NT | 0,064 |  | 0,047 | 0,075 |
| 0,229 | Left_vs_Right | LayerBetweenHemi | SD | 0,062 |  | 0,105 | 0,146 |
| 0,229 | Left_vs_Right | FullBetweenHemi | Full Network | 0,018 |  | 0,100 | 0,146 |
| 0,229 | Right | Layer | CD | 0,833 | 0,000 |  |  |
| 0,229 | Right | Layer | NT | 0,029 | 0,010 |  |  |
| 0,229 | Right | Layer | SD | 0,709 | 0,052 |  |  |
| 0,229 | Right | Pair | NT-CD | 0,005 | 0,003 |  |  |
| 0,229 | Right | Pair | NT-SD | 0,007 | 0,002 |  |  |
| 0,229 | Right | Pair | SD-CD | 0,706 | 0,035 |  |  |
| 0,229 | Right | Full | Full Network | 0,329 | 0,009 |  |  |
| 0,229 | Right | LayerComp | NT_vs_CD | -0,804 |  | 0,000 | 0,000 |
| 0,229 | Right | LayerComp | NT_vs_SD | -0,680 |  | 0,000 | 0,000 |
| 0,229 | Right | LayerComp | SD_vs_CD | -0,125 |  | 0,029 | 0,040 |
| 0,229 | Right | PairComp | NT-CD_vs_SD-CD | -0,702 |  | 0,000 | 0,000 |
| 0,229 | Right | PairComp | NT-SD_vs_NT-CD | 0,003 |  | 1,000 | 1,000 |
| 0,229 | Right | PairComp | NT-SD_vs_SD-CD | -0,699 |  | 0,000 | 0,000 |
| 0,250 | Left | Layer | CD | 0,622 | 0,000 |  |  |
| 0,250 | Left | Layer | NT | 0,084 | 0,039 |  |  |
| 0,250 | Left | Layer | SD | 0,763 | 0,088 |  |  |
| 0,250 | Left | Pair | NT-CD | 0,033 | 0,016 |  |  |
| 0,250 | Left | Pair | NT-SD | 0,060 | 0,009 |  |  |
| 0,250 | Left | Pair | SD-CD | 0,659 | 0,034 |  |  |
| 0,250 | Left | Full | Full Network | 0,325 | 0,017 |  |  |
| 0,250 | Left | LayerComp | NT_vs_CD | -0,539 |  | 0,000 | 0,000 |
| 0,250 | Left | LayerComp | NT_vs_SD | -0,679 |  | 0,000 | 0,000 |
| 0,250 | Left | LayerComp | SD_vs_CD | 0,140 |  | 0,065 | 0,083 |
| 0,250 | Left | PairComp | NT-CD_vs_SD-CD | -0,626 |  | 0,000 | 0,000 |
| 0,250 | Left | PairComp | NT-SD_vs_NT-CD | 0,027 |  | 0,267 | 0,308 |
| 0,250 | Left | PairComp | NT-SD_vs_SD-CD | -0,598 |  | 0,000 | 0,000 |
| 0,250 | Left_vs_Right | PairBetweenHemi | NT-CD | 0,033 |  | 0,011 | 0,029 |
| 0,250 | Left_vs_Right | PairBetweenHemi | NT-SD | 0,058 |  | 0,000 | 0,001 |
| 0,250 | Left_vs_Right | PairBetweenHemi | SD-CD | -0,022 |  | 0,575 | 0,671 |
| 0,250 | Left_vs_Right | LayerBetweenHemi | CD | -0,185 |  | 0,031 | 0,054 |
| 0,250 | Left_vs_Right | LayerBetweenHemi | NT | 0,069 |  | 0,013 | 0,032 |
| 0,250 | Left_vs_Right | LayerBetweenHemi | SD | 0,105 |  | 0,015 | 0,033 |
| 0,250 | Left_vs_Right | FullBetweenHemi | Full Network | 0,014 |  | 0,084 | 0,128 |
| 0,250 | Right | Layer | CD | 0,808 | 0,000 |  |  |
| 0,250 | Right | Layer | NT | 0,014 | 0,008 |  |  |
| 0,250 | Right | Layer | SD | 0,658 | 0,061 |  |  |
| 0,250 | Right | Pair | NT-CD | 0,000 | 0,000 |  |  |
| 0,250 | Right | Pair | NT-SD | 0,003 | 0,002 |  |  |
| 0,250 | Right | Pair | SD-CD | 0,681 | 0,036 |  |  |
| 0,250 | Right | Full | Full Network | 0,312 | 0,011 |  |  |
| 0,250 | Right | LayerComp | NT_vs_CD | -0,793 |  | 0,000 | 0,000 |
| 0,250 | Right | LayerComp | NT_vs_SD | -0,644 |  | 0,000 | 0,000 |
| 0,250 | Right | LayerComp | SD_vs_CD | -0,150 |  | 0,013 | 0,019 |
| 0,250 | Right | PairComp | NT-CD_vs_SD-CD | -0,681 |  | 0,000 | 0,000 |
| 0,250 | Right | PairComp | NT-SD_vs_NT-CD | 0,003 |  | 1,000 | 1,000 |
| 0,250 | Right | PairComp | NT-SD_vs_SD-CD | -0,678 |  | 0,000 | 0,000 |
| 0,290 | Left | Layer | CD | 0,578 | 0,000 |  |  |
| 0,290 | Left | Layer | NT | 0,047 | 0,027 |  |  |
| 0,290 | Left | Layer | SD | 0,705 | 0,092 |  |  |
| 0,290 | Left | Pair | NT-CD | 0,000 | 0,000 |  |  |
| 0,290 | Left | Pair | NT-SD | 0,036 | 0,007 |  |  |
| 0,290 | Left | Pair | SD-CD | 0,590 | 0,038 |  |  |
| 0,290 | Left | Full | Full Network | 0,282 | 0,016 |  |  |
| 0,290 | Left | LayerComp | NT_vs_CD | -0,531 |  | 0,000 | 0,000 |
| 0,290 | Left | LayerComp | NT_vs_SD | -0,659 |  | 0,000 | 0,000 |
| 0,290 | Left | LayerComp | SD_vs_CD | 0,128 |  | 0,116 | 0,142 |
| 0,290 | Left | PairComp | NT-CD_vs_SD-CD | -0,590 |  | 0,000 | 0,000 |
| 0,290 | Left | PairComp | NT-SD_vs_NT-CD | 0,036 |  | 0,022 | 0,031 |
| 0,290 | Left | PairComp | NT-SD_vs_SD-CD | -0,553 |  | 0,000 | 0,000 |
| 0,290 | Left_vs_Right | PairBetweenHemi | NT-CD | 0,000 |  | 1,000 | 1,000 |
| 0,290 | Left_vs_Right | PairBetweenHemi | NT-SD | 0,034 |  | 0,001 | 0,003 |
| 0,290 | Left_vs_Right | PairBetweenHemi | SD-CD | -0,039 |  | 0,374 | 0,485 |
| 0,290 | Left_vs_Right | LayerBetweenHemi | CD | -0,179 |  | 0,044 | 0,074 |
| 0,290 | Left_vs_Right | LayerBetweenHemi | NT | 0,032 |  | 0,241 | 0,325 |
| 0,290 | Left_vs_Right | LayerBetweenHemi | SD | 0,102 |  | 0,015 | 0,033 |
| 0,290 | Left_vs_Right | FullBetweenHemi | Full Network | -0,007 |  | 0,457 | 0,571 |
| 0,290 | Right | Layer | CD | 0,756 | 0,000 |  |  |
| 0,290 | Right | Layer | NT | 0,014 | 0,008 |  |  |
| 0,290 | Right | Layer | SD | 0,603 | 0,064 |  |  |
| 0,290 | Right | Pair | NT-CD | 0,000 | 0,000 |  |  |
| 0,290 | Right | Pair | NT-SD | 0,003 | 0,002 |  |  |
| 0,290 | Right | Pair | SD-CD | 0,629 | 0,037 |  |  |
| 0,290 | Right | Full | Full Network | 0,289 | 0,011 |  |  |
| 0,290 | Right | LayerComp | NT_vs_CD | -0,742 |  | 0,000 | 0,000 |
| 0,290 | Right | LayerComp | NT_vs_SD | -0,589 |  | 0,000 | 0,000 |
| 0,290 | Right | LayerComp | SD_vs_CD | -0,153 |  | 0,017 | 0,024 |
| 0,290 | Right | PairComp | NT-CD_vs_SD-CD | -0,629 |  | 0,000 | 0,000 |
| 0,290 | Right | PairComp | NT-SD_vs_NT-CD | 0,003 |  | 1,000 | 1,000 |
| 0,290 | Right | PairComp | NT-SD_vs_SD-CD | -0,626 |  | 0,000 | 0,000 |

**Abbreviations**: Thres = threshold. See Supplementary Figure 6 for density plot.

**Supplementary Table 15**: Community membership

| **Hemi** | **Community ID** | **Node number** | **Members** |
| --- | --- | --- | --- |
| Left | 1 | 21 | CC_MidA, CODES, CPT_F, CS_S, C_FP, DMN_B_PFCd_2, DS_B, DS_F, FAT, FLU_A, FLU_P, RST, SLF1, SLF2, STROOP_D, STROOP_ID, STROOP_R, SVA_B_PFCmp_2, TMT_A, TMT_BA, TR_S |
| Left | 2 | 16 | AC, AF, CC_P, C_PH, DMN_B_Temp_2, DO80, EMC, F, ILF, LimbA_TP_2, MdLF, RLRI_E, RLRI_FR, RLRI_TR, SVA_A_Ins_2, UF |
| Left | 3 | 10 | CC_A, CS_A, ContA_PFCl_2, DMN_B_PFCv_2, DMN_B_PFCv_3, IFOF, LimbB_OFC_2, PPTT, SVA_A_Ins_3, TR_A |
| Right | 8 | 25 | BELLS, CC_A, CC_MidA, CODES, CPT_F, CS_A, CS_S, C_FP, C_FPH, DMN_A_PFCd_2, DMN_B_PFCd_2, FAT, FLU_A, FLU_P, LB, RST, SLF1, SLF2, STROOP_D, STROOP_R, SVA_A_FrMed_2, SVA_B_PFCmp_2, TMT_BA, TR_A, TR_S |
| Right | 5 | 21 | AC, AF, CC_P, C_PH, C_PHP, ContA_PFCl_2, ContB_Temp_2, DMN_B_PFCv_2, DMN_B_PFCv_3, EMC, F, FIG_R, IFOF, ILF, LimbA_TP_2, LimbB_OFC_2, SLF3, SMB_Aud_2, SVA_A_Ins_2, TP_2, UF |
| Right | 3 | 3 | DS_B, DS_F, RLRI_E |
| Right | 4 | 1 | FIG_C |
| Right | 6 | 1 | RLRI_FR |
| Right | 7 | 1 | RLRI_TR |
| Right | 1 | 1 | STROOP_ID |
| Right | 2 | 1 | TMT_A |

Note: Communities are sorted by node number.

**Supplementary Table 16:** Silhouette coefficients for all t-SNE and UMAP parameter configurations used in the geometric cross-validation of Louvain communities.

| **t-SNE** | | |  | **UMAP** | | | |
| --- | --- | --- | --- | --- | --- | --- | --- |
| **Hemisphere** | **Perplexity** | **Silhouette** |  | **Hemisphere** | **n_neighbors** | **min_dist** | **Silhouette** |
| Left | 5 | 0.442 |  | Left | 5 | 0.1 | 0.369 |
| Left | 10 | 0.469 |  | Left | 5 | 0.5 | 0.213 |
| Left | 15 | 0.459 |  | Left | 10 | 0.1 | 0.541 |
| Left | 20 | 0.468 |  | Left | 10 | 0.5 | 0.453 |
| Left | 25 | 0.429 |  | Left | 15 | 0.1 | 0.506 |
| Left | 30 | 0.441 |  | Left | 15 | 0.5 | 0.443 |
| Left | 35 | 0.437 |  | Left | 20 | 0.1 | 0.479 |
| Left | 40 | 0.438 |  | Left | 20 | 0.5 | 0.43 |
| Left | 45 | 0.456 |  | Left | 25 | 0.1 | 0.474 |
| Right | 5 | -0.013 |  | Left | 25 | 0.5 | 0.424 |
| Right | 10 | 0.102 |  | Left | 30 | 0.1 | 0.485 |
| Right | 15 | 0.194 |  | Left | 30 | 0.5 | 0.417 |
| Right | 20 | 0.18 |  | Right | 5 | 0.1 | -0.111 |
| Right | 25 | 0.164 |  | Right | 5 | 0.5 | -0.08 |
| Right | 30 | 0.169 |  | Right | 10 | 0.1 | 0.09 |
| Right | 35 | 0.161 |  | Right | 10 | 0.5 | 0.039 |
| Right | 40 | 0.151 |  | Right | 15 | 0.1 | 0.119 |
| Right | 45 | 0.154 |  | Right | 15 | 0.5 | 0.065 |
| Right | 50 | 0.163 |  | Right | 20 | 0.1 | 0.143 |
|  |  |  |  | Right | 20 | 0.5 | 0.054 |
|  |  |  |  | Right | 25 | 0.1 | 0.078 |
|  |  |  |  | Right | 25 | 0.5 | 0.05 |
|  |  |  |  | Right | 30 | 0.1 | 0.088 |
|  |  |  |  | Right | 30 | 0.5 | 0.066 |

Description: For each hemisphere, multilayer node features were embedded into two dimensions using a range of t-SNE and UMAP settings. Silhouette coefficients were computed using Louvain community assignments as labels, providing a descriptive index of how well the low-dimensional geometry preserved the separation of these communities. Values close to +1 indicate strong community separation, values near zero reflect overlapping or graded boundaries, and negative values indicate misalignment. Left-hemisphere communities showed consistently positive and relatively high silhouette values, whereas right-hemisphere values were lower but stable across parameters, consistent with a more distributed organisation of the multilayer feature space.

­­­

**Supplementary Table 17**: Stability analysis using Normalized Mutual Information (MMI)

| **Hemisphere** | **mean_nmi** | **std_nmi** | **min_nmi** | **max_nmi** | **num_comparisons** |
| --- | --- | --- | --- | --- | --- |
| Left | 0.80 | 0.17 | 0.39 | 1 | 4950 |
| Right | 0.91 | 0.11 | 0.69 | 1 | 4950 |

**Supplementary Table 18**: Descriptive statistics for weighted degree centrality (strength)

| **Hemi** | **Type** | **Layer** | **median** | **mean** | **std** | **min** | **max** |
| --- | --- | --- | --- | --- | --- | --- | --- |
| Left | Layer Pairs | NT*i*-SD | 0.4614 | 0.6879 | 0.8964 | 0 | 3.7364 |
| Left | Layer Pairs | NT*i*-CD | 0 | 0.1674 | 0.3202 | 0 | 0.9842 |
| Right | Layer Pairs | NT*i*-SD | 0 | 0.2245 | 0.4512 | 0 | 1.4550 |
| Right | Layer Pairs | NTi-CD | 0 | 0.0931 | 0.2027 | 0 | 0.7830 |
| Left | All layers | NT*i*-SD-CD | 0.4640 | 0.8552 | 1.1489 | 0 | 4.7206 |
| Right | All layers | NT*i*-SD-CD | 0 | 0.3176 | 0.5571 | 0 | 1.6918 |

Note: Given the expected positive relationship between NT and brain damage, a threshold of R ≥ 0.175 was used to define two nodes as connected. *i* means *individual*

**Supplementary Table 19**: Descriptive statistics for weighted closeness centrality

| **Hemi** | **Type** | **Layer** | **median** | | **mean** | | **std** | | **min** | | **max** | |  |
| --- | --- | --- | --- | --- | --- | --- | --- | --- | --- | --- | --- | --- | --- |
| Left | Layer Pairs | NT*i*-SD | | 0.6735 | | 0.5301 | | 0.3300 | | 0 | | 0.9177 | |
| Left | Layer Pairs | NT*i*-CD | | 0 | | 0.2688 | | 0.3848 | | 0 | | 0.9613 | |
| Right | Layer Pairs | NT*i*-SD | | 0 | | 0.2078 | | 0.3252 | | 0 | | 0.8084 | |
| Right | Layer Pairs | NT*i*-CD | | 0 | | 0.1267 | | 0.2331 | | 0 | | 0.6470 | |
| Left | All layers | NT*i*-SD-CD | | 0.6738 | | 0.2892 | | 0.3500 | | 0 | | 0.9546 | |
| Right | All layers | NT*i*-SD-CD | | 0 | | 0.2892 | | 0.3252 | | 0 | | 0.8084 | |

Note: Given the expected positive relationship between NT and brain damage, a threshold of R ≥ 0.175 was used to define two nodes as connected. *i* means *individual*

**Supplementary Table 20**: Results from weighted degree centrality analyses

| **Hemisphere** | **Contrast** | **Mean difference** | ***p*** | ***p*_FDR_** |
| --- | --- | --- | --- | --- |
| Left | NTi-SD vs NTi-CD | 0.5205 | 0.0017 | 0.0051 |
| Left | NTi-SD vs NTi-SD-CD | -0.1674 | 0.0618 | 0.1029 |
| Left | NTi-CD vs NTi-SD-CD | -0.6879 | 0.0007 | 0.0042 |
| Right | NTi-SD vs NTi-CD | 0.1314 | 0.3671 | 0.3672 |
| Right | NTi-SD vs NTi-SD-CD | -0.0931 | 0.1264 | 0.1518 |
| Right | NTi-CD vs NTi-SD-CD | -0.2245 | 0.0630 | 0.1029 |
| Left vs Right | NTi-SD | 0.4634 | 0.0578 | 0.1236 |
| Left vs Right | NTi-CD | 0.0743 | 0.455 | 0.4554 |
| Left vs Right | NTi-SD-CD | 0.5377 | 0.0823 | 0.1236 |

Note: p-values are derived from a 10,000-permutation procedure.

**Supplementary Table 21**: Results from weighted closeness centrality analyses

| **Hemisphere** | **Contrast** | **Mean difference** | ***p*** | ***p*_FDR_** |
| --- | --- | --- | --- | --- |
| Left | NTi-SD vs NTi-CD | 0.2613 | 0.0973 | 0.147 |
| Left | NTi-SD vs NTi-SD-CD | -0.1005 | 0.0983 | 0.147 |
| Left | NTi-CD vs NTi-SD-CD | -0.3617 | 0.0078 | 0.0414 |
| Right | NTi-SD vs NTi-CD | 0.0811 | 0.4016 | 0.4016 |
| Right | NTi-SD vs NTi-SD-CD | -0.0814 | 0.2038 | 0.2447 |
| Right | NTi-CD vs NTi-SD-CD | -0.1625 | 0.0172 | 0.0519 |
| Left vs Right | NTi-SD | 0.3223 | 0.0138 | 0.0207 |
| Left vs Right | NTi-CD | 0.1422 | 0.2239 | 0.2194 |
| Left vs Right | NTi-SD-CD | 0.3414 | 0.0077 | 0.0191 |

Note: p-values are derived from a 10,000-permutation procedure.

**Supplementary Table 22**: Mapping relationships between individual NT nodes and CD and NT layers (threshold ≥ 0.229. p ≤ 0.01)

| **Hemi** | **NT tasks** | **count** | | **Mapping type** | | **Connections** | |
| --- | --- | --- | --- | --- | --- | --- | --- |
|  |  | **SD** | **CD** | **SD** | **CD** | **SD** | **CD** |
| Left | CODES | 1 | 0 | many-to-one | none | SLF1 |  |
| Left | DO80 | 3 | 0 | many-to-many | none | C_PH, F, ILF |  |
| Left | DS_F | 1 | 0 | many-to-one | none | TR_S |  |
| Left | DS_B | 0 | 0 | none | none |  |  |
| Left | FLU_A | 0 | 0 | none | none |  |  |
| Left | FLU_P | 10 | 3 | many-to-many | one-to-many | CC_A, CC_MidA, CPT_F, CS_A, CS_S, FAT, RST, SLF1, TR_A, TR_S | SVA_B_PFCmp_2, ContA_PFCl_2, DMN_B_PFCd_2 |
| Left | PPTT | 0 | 0 | none | none |  |  |
| Left | RLRI_E | 0 | 0 | none | none |  |  |
| Left | RLRI_FR | 0 | 0 | none | none |  |  |
| Left | RLRI_TR | 2 | 2 | many-to-many | one-to-many | AC, ILF | LimbA_TP_2, DMN_B_Temp_2 |
| Left | STROOP_D | 2 | 0 | many-to-many | none | CS_S, TR_S |  |
| Left | STROOP_R | 1 | 0 | many-to-one | none | CS_S |  |
| Left | STROOP_ID | 0 | 0 | none | none |  |  |
| Left | TMT_A | 0 | 0 | none | none |  |  |
| Left | TMT_BA | 1 | 0 | one-to-one | none | SLF2 |  |
| Right | LB | 0 | 0 | none | none |  |  |
| Right | BELLS | 1 | 0 | one-to-one | none | C_FPH |  |
| Right | CODES | 0 | 0 | none | none |  |  |
| Right | DS_F | 0 | 0 | none | none |  |  |
| Right | DS_B | 0 | 0 | none | none |  |  |
| Right | FIG_C | 0 | 0 | none | none |  |  |
| Right | FIG_R | 0 | 0 | none | none |  |  |
| Right | FLU_A | 1 | 1 | one-to-one | one-to-one | RST | ContA_PFCl_2 |
| Right | FLU_P | 0 | 0 | none | none |  |  |
| Right | RLRI_E | 0 | 0 | none | none |  |  |
| Right | RLRI_FR | 0 | 0 | none | none |  |  |
| Right | RLRI_TR | 0 | 0 | none | none |  |  |
| Right | STROOP_D | 1 | 0 | one-to-one | none | CPT_F |  |
| Right | STROOP_R | 0 | 0 | none | none |  |  |
| Right | STROOP_ID | 0 | 0 | none | none |  |  |
| Right | TMT_A | 0 | 0 | none | none |  |  |
| Right | TMT_BA | 0 | 0 | none | none |  |  |

**Supplementary Table 23**: SD target connections (threshold ≥ 0.175, *p* ≤ 0.05)

| **SD_Node** | **NT_Connections** | **Hemisphere** |
| --- | --- | --- |
| AC | 1 | Left |
| AF | 1 | Left |
| C_FP | 2 | Left |
| C_PH | 2 | Left |
| CC_A | 1 | Left |
| CC_MidA | 1 | Left |
| CC_P | 1 | Left |
| CPT_F | 1 | Left |
| CS_A | 1 | Left |
| CS_S | 5 | Left |
| EMC | 0 | Left |
| F | 1 | Left |
| FAT | 1 | Left |
| IFOF | 1 | Left |
| ILF | 2 | Left |
| MdLF | 0 | Left |
| RST | 1 | Left |
| SLF1 | 5 | Left |
| SLF2 | 5 | Left |
| TR_A | 1 | Left |
| TR_S | 7 | Left |
| UF | 1 | Left |
| AC | 0 | Right |
| AF | 0 | Right |
| C_FP | 1 | Right |
| C_FPH | 2 | Right |
| C_PH | 0 | Right |
| C_PHP | 0 | Right |
| CC_A | 1 | Right |
| CC_MidA | 1 | Right |
| CC_P | 0 | Right |
| CPT_F | 2 | Right |
| CS_A | 1 | Right |
| CS_S | 2 | Right |
| EMC | 0 | Right |
| F | 0 | Right |
| FAT | 1 | Right |
| IFOF | 0 | Right |
| ILF | 0 | Right |
| RST | 2 | Right |
| SLF1 | 1 | Right |
| SLF2 | 1 | Right |
| SLF3 | 0 | Right |
| TR_A | 1 | Right |
| TR_S | 2 | Right |
| UF | 0 | Right |

**Supplementary Table 24**: CD target connections (threshold ≥ 0.175, *p* ≤ 0.05)

| **CD_Node** | **NT_Connections** | **Hemisphere** |
| --- | --- | --- |
| SVA_A_Ins_2 | 1 | Left |
| SVA_A_Ins_3 | 0 | Left |
| SVA_B_PFCmp_2 | 1 | Left |
| LimbB_OFC_2 | 1 | Left |
| LimbA_TP_2 | 1 | Left |
| ContA_PFCl_2 | 3 | Left |
| DMN_B_Temp_2 | 1 | Left |
| DMN_B_PFCd_2 | 1 | Left |
| DMN_B_PFCv_2 | 1 | Left |
| DMN_B_PFCv_3 | 1 | Left |
| SMB_Aud_2 | 1 | Right |
| SVA_A_Ins_2 | 0 | Right |
| SVA_A_FrMed_2 | 1 | Right |
| SVA_B_PFCmp_2 | 1 | Right |
| LimbB_OFC_2 | 0 | Right |
| LimbA_TP_2 | 0 | Right |
| ContA_PFCl_2 | 2 | Right |
| ContB_Temp_2 | 0 | Right |
| DMN_A_PFCd_2 | 1 | Right |
| DMN_B_PFCd_2 | 1 | Right |
| DMN_B_PFCv_2 | 0 | Right |
| DMN_B_PFCv_3 | 1 | Right |
| TP_2 | 0 | Right |

**Supplementary Table 25**: SD target connections (threshold ≥ 0.229, p ≤ 0.01)

| **SD_Node** | **NT_Connections** | **Hemisphere** |
| --- | --- | --- |
| AC | 1 | Left |
| AF | 0 | Left |
| C_FP | 0 | Left |
| C_PH | 1 | Left |
| CC_A | 1 | Left |
| CC_MidA | 1 | Left |
| CC_P | 0 | Left |
| CPT_F | 1 | Left |
| CS_A | 1 | Left |
| CS_S | 3 | Left |
| EMC | 0 | Left |
| F | 1 | Left |
| FAT | 1 | Left |
| IFOF | 0 | Left |
| ILF | 2 | Left |
| MdLF | 0 | Left |
| RST | 1 | Left |
| SLF1 | 2 | Left |
| SLF2 | 1 | Left |
| TR_A | 1 | Left |
| TR_S | 3 | Left |
| UF | 0 | Left |
| AC | 0 | Right |
| AF | 0 | Right |
| C_FP | 0 | Right |
| C_FPH | 1 | Right |
| C_PH | 0 | Right |
| C_PHP | 0 | Right |
| CC_A | 0 | Right |
| CC_MidA | 0 | Right |
| CC_P | 0 | Right |
| CPT_F | 1 | Right |
| CS_A | 0 | Right |
| CS_S | 0 | Right |
| EMC | 0 | Right |
| F | 0 | Right |
| FAT | 0 | Right |
| IFOF | 0 | Right |
| ILF | 0 | Right |
| RST | 1 | Right |
| SLF1 | 0 | Right |
| SLF2 | 0 | Right |
| SLF3 | 0 | Right |
| TR_A | 0 | Right |
| TR_S | 0 | Right |
| UF | 0 | Right |

**Supplementary Table 26**: CD target connections (threshold ≥ 0.29, *p* ≤ 0.01)

| **CD_Node** | **NT_Connections** | **Hemisphere** |
| --- | --- | --- |
| SVA_A_Ins_2 | 0 | Left |
| SVA_A_Ins_3 | 0 | Left |
| SVA_B_PFCmp_2 | 1 | Left |
| LimbB_OFC_2 | 0 | Left |
| LimbA_TP_2 | 1 | Left |
| ContA_PFCl_2 | 1 | Left |
| DMN_B_Temp_2 | 1 | Left |
| DMN_B_PFCd_2 | 1 | Left |
| DMN_B_PFCv_2 | 0 | Left |
| DMN_B_PFCv_3 | 0 | Left |
| SMB_Aud_2 | 0 | Right |
| SVA_A_Ins_2 | 0 | Right |
| SVA_A_FrMed_2 | 0 | Right |
| SVA_B_PFCmp_2 | 0 | Right |
| LimbB_OFC_2 | 0 | Right |
| LimbA_TP_2 | 0 | Right |
| ContA_PFCl_2 | 1 | Right |
| ContB_Temp_2 | 0 | Right |
| DMN_A_PFCd_2 | 0 | Right |
| DMN_B_PFCd_2 | 0 | Right |
| DMN_B_PFCv_2 | 0 | Right |
| DMN_B_PFCv_3 | 0 | Right |
| TP_2 | 0 | Right |
